# Body-responsive shape-memory polymers for biomedical applications

**DOI:** 10.1016/j.bioactmat.2025.12.054

**Published:** 2026-01-29

**Authors:** Ebrahim Tajik, Nima Reihani, Vahid Karamzadeh, Guosheng Tang, Hossein Ravanbakhsh

**Affiliations:** a Department of Biomedical Engineering, The University of Akron, Akron, OH, 44325, USA; b Department of Physical and Macromolecular Chemistry, Faculty of Science, Charles University, 128 40 Prague 2, Czech Republic; c Division of Engineering in Medicine, Department of Medicine, Brigham and Women's Hospital, Harvard Medical School, Cambridge, MA, 02139, USA

**Keywords:** Shape memory polymers, Physiological stimuli, Smart materials, Tissue engineering, Minimally invasive procedures

## Abstract

Shape memory polymers (SMPs) have emerged as versatile and adaptive materials in healthcare, offering transformative solutions for tissue repair and biomedical device interfaces. Their ability to undergo controlled shape changes in response to external stimuli has driven significant interest in developing smart implants for minimally invasive procedures. Precise material design and engineering that leverage physiological conditions, such as body temperature and bodily fluids, can unlock their potential for biomedical applications. This review focuses explicitly on SMPs activated by physiological stimuli, referred to here as “body-responsive” SMPs. By categorizing SMPs into temperature-responsive, water-responsive, and dual-responsive variants, their shape memory behavior is analyzed, with an emphasis on how the structural design governs the body-responsiveness of the SMPs. Current biomedical applications, including tissue engineering, vascular interventions, bioelectronic devices, and targeted drug delivery, are also highlighted to demonstrate the practical relevance and versatility of body-responsive SMPs. Additionally, emerging fabrication technologies are discussed to provide insight into current scalable production methods suitable for SMPs. Finally, challenges in the design and performance of SMPs are explored, and a vision for future advancements is presented, outlining a roadmap for translating SMPs into biomedical applications within clinical settings.

## Graphical abstract

**Figure:**
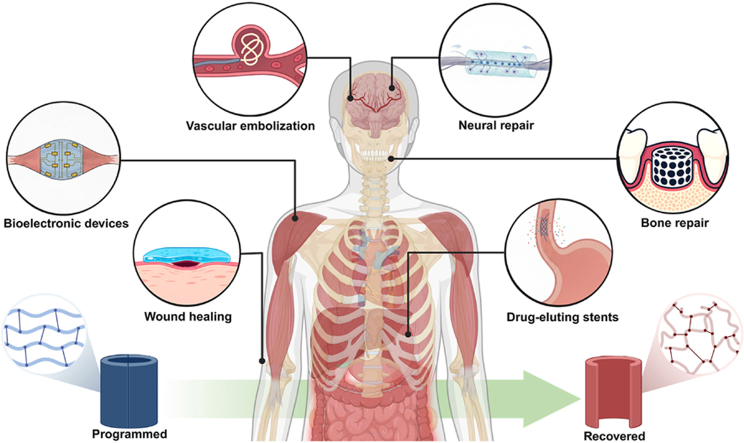

**Table:** Acronyms List

Acronyms	Description	Acronyms	Description	Acronyms	Description
**SMP**	Shape memory polymer	**TAPUA**	Trifunctional aliphatic polyurethane acrylate	**PVLCL**	Poly(valerolactone-co-caprolactone)
**SMB**	Shape memory behavior	**EA**	Ethyl acrylate	**PUU**	Polyurethane urea
**FDA**	Food and drug administration	**BA**	Butyl acrylate	**DEG**	Diethylene glycol
**PLA**	Poly lactic acid	**MMA**	Methyl methacrylate	**PCLUSe**	PCL- and selenocystamine-based polyurethane
**PCL**	Poly capro-lactone	**GMA**	Glycidyl methacrylate	**PEGMA**	Polyethylene glycol methacrylate
**TMP**	Temperature-responsive shape memory polymer	**BPE**	Bisphenol A	**PPAzSeb**	Poly(propylene azelate-co-propylene sebacate)
**T_trans_**	Transition temperature	**HBA**	4-hydroxybutyl acrylate	**CS**	Chitosan
**T_g_**	Glass transition temperature	**D230**	Poly(propylene glycol) bis(2-aminopropyl ether)	**CNC**	Cellulose nanocrystals
**T_m_**	Melting temperature	**PGS**	Polyglycerol sebacate	**WRAP**	Water-responsive shape-adaptive polymer
**WMP**	Water-responsive shape memory polymer	**PGD**	Polyglycerol dodecanoate	**PVA**	Polyvinyl alcohol
**R_f_**	Fixity ratio	**DDA**	Dodecanedioic acid	**PBF**	Polybutanetetrol fumarate
**R_r_**	Recovery ratio	**PPS**	Polypropylene sebacate	**PFOT**	Poly(fumaric acid-co-octadiene diepoxide-co-terephthalic acid)
**3D**	Three-dimensional	**PGSA**	Polyglycerol sebacate acrylate	**CNF**	Cellulose nanofibrils
**PDLLA**	Poly (D, L-lactide)	**PGDA**	Polyglycerol dodecanoate acrylate	**QCS**	Quaternized chitosan
**PTMC**	Polytrimethylene carbonate	**HEMA**	Hydroxyethyl methacrylate	**BIN**	N,N-bis(2-hydroxyethyl)isonicotinamide
**DLLA**	D, L-lactide	**PGA**	Poly (L-glutamic acid)	**PEGDA**	Polyethylene glycol diacrylate
**TMC**	Trimethyl carbonate	**PPDLDA**	Poly (ω-pentadecalactone) diacrylate	**EDTA**	Ethylenediaminetetraacetic acid
**Sn(Oct)_2_**	tin octanoate	**PEG**	Polyethylene glycol	**PTHF**	Polytetrahydrofuran
**AESO**	Acrylated epoxidized soybean oil	**PGCL**	Poly (glycolide-co-caprolactone)	**1D**	One-dimensional
**4D**	Four-dimensional	**PLLA**	Poly (L-lactide)	**VP**	Vat polymerization
**PU**	Polyurethane	**PDLA**	Poly (D-lactide)	**DLP**	Digital light processing
**MW**	Molecular weight	**PLCL**	Poly (lactide-co-caprolactone)	**LCD**	Liquid crystal display
**HPED**	N, N, N′, N′-tetrakis(2-hydroxypropyl) ethylenediamine	**PLGA**	Poly (lactide-co-glycolic)	**CLIP**	Continuous liquid interface production
**TEA**	Triethanolamine	**BDO**	Butanediol	**HARP**	High-area rapid printing
**HDI**	1,6-diisocyanatehexane	**ISO**	Isosorbide	**SDF-1α**	Stromal cell-derived factor 1α
**DDFD**	4,4-dimethyldihydrofuran-2,3-dione	**MDI**	4,4-methylenediphenyl diisocyanate	**PFPE**	Perfluoropolyether
**tBA**	tert-butyl acrylate	**IU**	Imidazoline urea	**H&E**	Hematoxylin and eosin
**BMA**	Benzyl methacrylate	**TA**	Tannic acid	**GO**	Graphene oxide
**2-EHA**	2-ethylhexyl acrylate	**POSS**	Polyhedral oligomeric silsesquioxane	**RGO**	Reduced graphene oxide
**ACMO**	4-acryloylmorpholine	**PDMS**	Polydimethylsiloxane	**TRL**	Technology readiness level
**NdFeB**	Neodymium iron boron	**VL**	δ-valerolactone	**NaCl**	Sodium chloride

## 1 Introduction

**Fig. 1:**
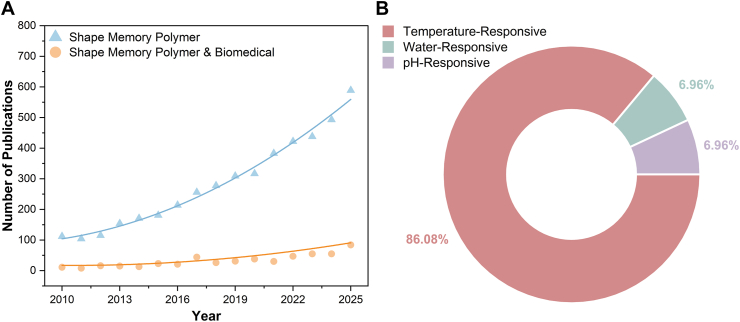
Infographic on the current literature on SMPs. A) Increasing publication trend from 2010 to 2025 using the keywords “Shape memory polymer” and “Shape memory polymer and biomedical”. B) Breakdown of physiological stimuli referenced in published SMP studies, identified using “Shape memory polymer” combined with each stimulus keyword. Data were extracted from Scopus as of November 2025.

Shape memory polymers (SMPs) are a subclass of smart materials that undergo temporary macroscopic shape changes in response to an external stimulus and return to their original shape once the stimulus is removed [[1], [2], [3]]. Depending on their programming, SMPs can respond to a variety of triggers, including physical stimuli (such as temperature [4,5], water [6], light [7,8], and magnetic/electrical fields [9,10]), chemical stimuli (such as pH [11,12] and ion transfer [13,14]), or biological stimuli [15,16]. Each of these stimuli initiates a distinct molecular mechanism that leads to shape change [17]. This unique feature positions SMPs as promising candidates for various applications, including flexible electronics [18,19], soft robotics [20,21], smart textiles [[22], [23], [24]], and aeronautical structures, as evidenced by the growing number of publications (Fig. 1A) [[25], [26], [27], [28]]. Given their potential in minimally invasive procedures, a similar upward trend is observed in the study of SMPs for different biomedical applications [[29], [30], [31], [32]], such as tissue regeneration, medical device fabrication (e.g., stents, implants, sutures), and drug delivery systems [[33], [34], [35], [36], [37]].

**Fig. 2:**
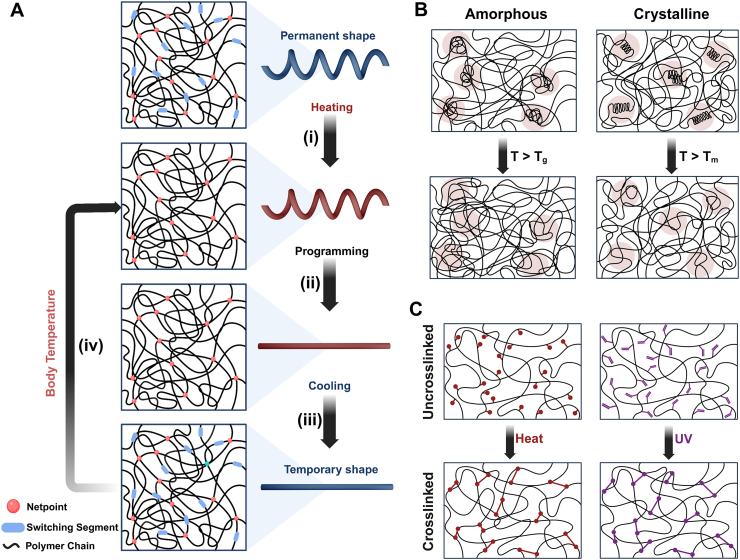
Illustration of body-responsive TMPs elements and their behavior. Schematic of A) General mechanism of body-responsive TMPs from programming to recovery: (i) switching segments reorganize upon heating above body temperature, (ii) applied force programs the structure into a desired shape, (iii) the switching segments reform upon cooling below body temperature, and (iv) the structure regains its shape upon reheating. B) Amorphous and crystalline switching segments (highlighted in pink circles) that switch, upon heating above their transition temperature (*T*_*g*_ or *T*_*m*_*)*, and C) Formation of chemical netpoints between existing functional groups via heat or exposure to UV light.

The first polymer exhibiting shape memory behavior (SMB) that was used for medical applications was a methacrylic-based resin developed for dental restoration in 1941 [38,39]. This innovation led to SMPs that received approval for medical use from the U.S. Food and Drug Administration (FDA). Notably, devices such as the self-tightening suture DYNACORD™ [40], the soft tissue anchor Eclipse™ [41], and the orthopedic suture anchor Morphix® [42], have been used for bone regeneration. Due to their unique structure, these shape-memory devices can regain their permanent shape upon implantation while withstanding mechanical loading cycles [43]. One of the most recent technologies approved by the FDA is IMPEDE-FX, a device constructed from polyurethane foam as an embolization plug to treat blood abnormalities [44,45].

Primarily, SMPs are composed of two distinct segments: a switching segment and a stationary segment. The former is responsible for the shape change and reversibility, while the latter maintains the overall integrity of the structure [3,33]. Depending on their design, stationary segments can have chemical or physical crosslinks, which provide stability to the polymer network. The switching segments, however, may vary depending on the stimulus that controls reversibility. Examples of such segments include crystalline domain formation in a semicrystalline polymer network, molecular interactions, and percolating networks [37]. The shape change, therefore, is attributed to physical and chemical interactions between molecular chains or segments [6]. Thus, SMB is not an inherent property limited to a specific family of polymers but rather a structure-dependent property that can be engineered across a wide range of polymers [3,17]. The structural versatility of SMPs allows for precise tailoring to achieve target characteristics, such as mechanical stability, biodegradability, and biocompatibility [46,47].

Shape changes in SMPs can be triggered by the body's physiological environment, such as temperature, water, and pH. Synthetic polymers, such as polyglycolic acid, polylactic acid (PLA), polycaprolactone (PCL), and different derivatives of polyesters and polyurethanes, including their copolymers, have been extensively utilized as body-responsive SMPs [[48], [49], [50], [51], [52]]. Several studies have been conducted on the chemical modification of polymers to tune their SMB for human body conditions [[53], [54], [55]]. Engineering the polymer structure and functional moieties based on the target application has the potential to adjust SMB, as showcased by the development of chemically modified PCL-based stents capable of self-expanding within the arteries [4].

Temperature-responsive shape memory polymers (TMPs) are a well-studied subclass of SMPs (Fig. 1B) triggered by temperature changes [3,37]. TMPs can undergo shape changes at their transition temperature (*T*_*trans*_), which can correspond to either the glass transition temperature (*T*_*g*_) for amorphous regions or the melting temperature (*T*_*m*_) for crystalline regions. Heating above *T*_*trans*_ causes the polymer chains to transition from a rigid to an elastic state, allowing their microstructure to deform under mechanical loading [2,3,37]. The polymer structure can then be fixed by decreasing the temperature below *T*_*trans*_ while the mechanical loading is still in place. Exposure to the temperature at or above *T*_*trans*_ can cause the TMP to revert to its original shape. This principle underlies the design of TMPs for biomedical applications, where devices with a *T*_*trans*_ near body temperature (37 °C) can expand upon implantation in the body.

A major obstacle in developing TMPs for biomedical implants is their relatively high *T*_*trans*_, which limits their application for in vivo implantation [6,[56], [57], [58]]. Therefore, alternative physiological stimulants have been considered to resolve this issue. The abundance of water, as a mild stimulant, in the human body spurred the development of water-responsive SMPs (WMPs), making them a feasible option for in vivo applications. A widely adopted, but not exclusive, structural design for WMPs involves integrating hydrophilic and hydrophobic blocks in a network to serve as the switching and stationary segments, respectively [6,58]. Due to the structural difference between these segments, WMPs can also undergo shape changes. Similar to TMPs, the shape-change process in WMPs is reversible, meaning that the polymer can shift between its programmed and permanent shapes by absorbing or releasing water.

Several review papers available in the literature have addressed the fundamentals and biomedical-related research on SMPs [[59], [60], [61]]. There are also focused surveys available on areas such as tissue repair [3,37], multifunctional SMPs [62], SMP fibers [63,64], and SMP composites [65]. However, the existing reviews emphasize the broad stimuli categories, and scant attention has been paid to the SMPs that are exclusively activated by physiological conditions, without any need for external manipulation. To date, no review frames SMPs using criteria explicitly tied to physiological triggers, e.g., body temperature and water, positioning “body-responsive” SMPs as distinct and timely lenses to accommodate that.

Herein, we classify body-responsive SMPs into temperature-, water-, and dual-responsive SMPs, rendering them suitable for biomedical applications. We briefly review the molecular mechanisms governing the shape-changing behavior of the categories. We discuss SMB in parallel with the polymer composition to highlight the role of the polymer's chemical structure in determining SMB. Various fabrication methods, along with the current or potential applications of these polymers, are then elaborated. Furthermore, we discuss the challenges in developing and implementing SMP constructs in biomedical applications, while also outlining future directions in the field. Our objective is to curate and critically analyze a collection of tunable body-responsive SMPs, facilitating future advancements in biomedical engineering.

## 2 Temperature-responsive SMPs

Fundamentally, temperature-responsive SMPs (TMPs) exhibit entropy-driven behavior originating from the randomly coiled structure of the polymer chains [52,61]. Thermoviscoelastic theory can model TMPs by defining molecular chains as intertwined springs in a high-entropy state [29,37]. As the temperature rises, these chains become mobile and exhibit thermoviscoelasticity. Applying an external force orients the chains, causing a shape change that can be “programmed” by decreasing the temperature. Upon reheating, the stored elastic energy enables the TMP to “recover” its original shape. Phase transition theory can also be used to describe this behavior, where stationary regions and temperature-sensitive switching segments influence both programming and recovery processes in TMPs [29,52]. Fig. 2A shows the schematic mechanism of shape change in body-responsive TMPs from programming to recovery. To quantify SMB, the shape fixity ratio (*R*_*f*_) and shape recovery ratio (*R*_*r*_) are measured, as these represent the ability of the SMP to maintain the temporary shape and recover to its original shape, respectively [[66], [67], [68]]. *R*_*f*_ reflects the capability of switching segments to stabilize the temporary shape during programming, while *R*_*r*_ indicates the efficiency of the shape recovery. Commonly, these parameters are quantified using parameters such as linear and angular deformation, measured instrumentally or visually, using equations (1), (2)).

(1) $$R_{f}\left(N\right)=\frac{X_{u}\left(N\right)}{X_{m}}\times 100$$

(2) $$R_{r}\;\left(N\right)=\frac{X_{u}\left(N\right)-X_{p}\left(N\right)}{X_{u}\left(N\right)}\times 100$$

Where *N* represents the shape-memory loading cycle, *X*_*m*_ is the maximum deformation applied during programming above *T*_*trans*_ in a given cycle, *X*_*u*_ is the retained deformation in the stress-free state after unloading below *T*_*trans*_, and *X*_*p*_ corresponds to the small residual deformation after reheating above *T*_*trans*_. Stimuli-dependent mechanical differences between segments influence these parameters, which are crucial in assessing the SMP's performance.

Switching segments influence the programming and recovery processes in TMPs and dictate the transition temperature, *T*_*trans*_ [[69], [70], [71]]. Consequently, altering the molecular structures of switching segments is a straightforward strategy to create body-responsive TMPs by setting the *T*_*trans*_ at the body temperature. The glass transition temperature, *T*_*g*_, can be modulated by polar groups, chain length, and side groups on the polymer chain. Generally, any modification that reduces chain mobility and increases entanglements (the intertwined coiled microstructures originating from long polymer chains) and chain stiffness results in a higher *T*_*g*_. As shown in Fig. 2B, at *T < T*_*g*_, entanglements drive the shape-memory response. In addition to entanglement-driven mechanisms, polymer chains can self-organize into a three-dimensional (3D) crystalline structure by aligning themselves based on the chains' chemical composition. To form a crystallite in a polymer structure, the polymer chains need regularity to facilitate alignment [72,73]. Any chemical hindrance or structural irregularity can decrease the crystallinity percentage and reduce *T*_*m*_ [74,75]. The stationary segments also impact the TMP's mechanical properties, mainly affecting the structure's ability to maintain its programmed shape [76,77]. The characteristics of the netpoints (such as the type, length, and density) formed via chemical and physical crosslinking can also help adjust *R*_*f*_ and *R*_*r*_.

### 2.1 Chemically crosslinked body-responsive TMPs

During chemical crosslinking, polymer chains link at multiple points, creating a 3D covalently bonded structure, termed a *polymer network* [78]. These linkages are mainly formed via thermal or UV crosslinking of a linear or branched polymer between existing groups on the chain. Examples include the reaction between carboxylic acid and hydroxyl groups in an ester or photocrosslinking of acrylate structures (Fig. 2C) [79]. The intended application and the desired material properties dictate the selection of a crosslinking mechanism. The following subsections are organized based on the chemically-crosslinked TMPS and their *T*_*trans*_ (transition temperature), as the point where shape changes occur. This temperature depends on the *T*_*g*_ (glass transition temperature) and the *T*_*m*_ (melting temperature) of the amorphous and crystalline switching segments, respectively. Engineering these segments that consequently change their transition temperature allows for the design of body-responsive TMPs tailored to body condition (37 °C).

#### 2.1.1 Chemically crosslinked amorphous TMPs (T_trans_ = T_g_)

**Fig. 3:**
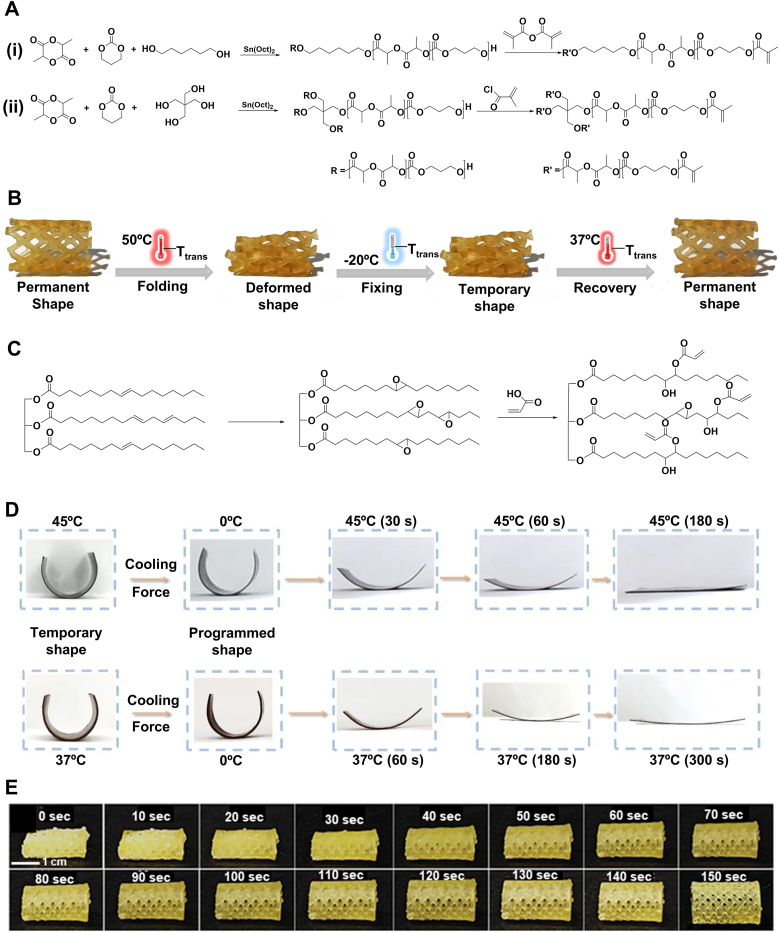
Lactide- and plant oil-based TMP synthesis and their shape memory performance. A) Synthetic route of (i) linear and (ii) four-armed photocrosslinkable poly (LLA-co-TMC) copolymer. B) Shape programming and recovery of a 3D-printed poly (LLA-co-TMC) copolymer network as a drug-eluting stent with 80 % PLLA. Folding and fixing are above and below *T*_*trans*_ (37 °C), respectively. Reproduced under terms of the CC-BY license [1]. Copyright 2023, The Authors, published by Elsevier. C) Epoxidation and acrylation reaction of soybean oil. D) Shape recovery of AESO sheets at two different temperatures. Reproduced with permission [2]. Copyright 2023, Wiley-VCH. E) Shape recovery behavior and time of the 4D-printed olive oil-based stent at room temperature. Reproduced with permission [3]. Copyright 2025, Wiley-VCH.

*Polylactide Copolymer Networks*: Poly (D, L-Lactide) (PDLLA) and polytrimethylene carbonate (PTMC) are both amorphous polymers with *T*_*trans*_ of 54 °C and −15 °C, respectively [80,81]. Previous studies have shown that copolymer networks formed by combining these polymers exhibit SMB. However, the high crosslinking density of these networks results in brittleness and poor mechanical properties, limiting their suitability for shape recovery. To enhance the toughness and flexibility of these copolymer networks, researchers copolymerized D, L-lactide (DLLA) and trimethyl carbonate (TMC) via ring-opening polymerization using 1,6-hexanediol as the initiator and tin octanoate (Sn(Oct)_2_) as the catalyst [80]. To achieve photo-crosslinking properties, the copolymer was then acrylated using methacrylic anhydride (Fig. 3A(i)). Reducing the ratio of DLLA to TMC resulted in a decrease in *T*_*trans*_ from 37 °C to 10 °C [[80], [81], [82], [83]]. This trend is justified by the polar interaction originating from the carbon–oxygen bonds in the polymer [80,82]. Reducing the DLLA content was found to decrease these interactions and facilitate polymer chain movements. The chains, therefore, required less energy to move, leading to lower *T*_*trans*_. The network created using this copolymer exhibited a higher *T*_*trans*_ than its physically crosslinked counterpart. The chemical netpoints enhanced the polymer's robustness by restricting chain mobility. Such a structure requires more energy to change shape; therefore, it has a higher *T*_*trans*_. Adjusting molar ratios ultimately leads to a mechanically strong polymer network with a *T*_*trans*_ of 30 °C for a 60:40 DLLA:TMC molar ratio.

In another study, a copolymer of DLLA and TMC was synthesized using pentaerythritol as an initiator, as schemed in Fig. 3A(ii) [84]. This approach introduced two additional functional groups, which reacted with methacryloyl chloride to form a UV-crosslinkable copolymer. By employing a high-functionality initiator, the resulting random four-arm copolymer exhibited a lower viscosity, making it suitable for vat photopolymerization 3D printing at elevated temperatures [84]. The copolymer with 80 % DLLA showed a *T*_*trans*_ value of about 32 °C, which is compatible with the self-recovery requirement in the body. Owing to the chemical netpoints formed by methacrylate groups, the 3D-printed structure could be programmed at 50 °C, fixed at 20 °C, and retrieved its permanent shape at body temperature, as shown in Fig. 3B. Notably, the higher percentage of DLLA led to a *T*_*trans*_ closer to body temperature. Compared to the TMPs fabricated using a bifunctional initiator, this network has a lower *T*_*trans*_ for the same molar ratio of DLLA-TMC [80,84]. The reduction in *T*_*trans*_ originated from substituting bifunctional for tetrafunctional initiators, which created more free volume between the polymer chains. The increased free volume facilitates greater flexibility in the polymer chains, as they require less energy for movement.

*Soybean Oil*: Plant-based oils form another category of chemically crosslinked amorphous materials employed as TMPs. Unsaturated plant-based oils undergo extensive epoxidation, converting carbon-carbon double bonds into highly reactive epoxide groups [85,86]. Soybean oil, in particular, can be epoxidized using organic and inorganic peroxides in the presence of a metal catalyst, with fumaric acid or acetic acid [87]. The resulting epoxy groups can then be substituted by acrylic acid under catalytic conditions, as illustrated in Fig. 3C. Epoxidized soybean oil and acylated epoxidized soybean oil (AESO) have been widely used in surface coating applications. Still, their potential for biomedical applications was first explored in 2016 [87,88]. Their studies demonstrated that the crosslinked AESO has a *T*_*trans*_ of 20 °C, which shows its capability for shape recovery at body temperature [88,89]. The acrylate groups formed netpoints, where fatty acid residues such as stearic, oleic, and linoleic acid functioned as pendant groups and switching segments. The temporary shape was fixed at −18 °C, and upon heating to 37 °C, the scaffold recovered within 1 min, highlighting its potential for rapid shape recovery in biomedical applications.

AESO was further used as a matrix for polydopamine nanoparticles grafted by 3-trimethoxysilyl propyl methacrylate to formulate an ink for four-dimensional (4D) printing [90]. The methacrylate groups on the surface of the nanoparticles enabled them to form a covalent bond with AESO, maintaining the overall shape of the construct after 4D printing. Meanwhile, the un-crosslinked parts of the AESO chains act as switching segments. As shown in Fig. 3D, the shape change was studied by fabricating a planar sheet of AESO, then rolling it, and fixing the shape at 0 °C. The rolled sheet regained its original shape within 180 s at 45 °C and within 300 s at 37 °C, demonstrating a five-fold longer recovery time as compared to pristine AESO at 37 °C [88,90]. This delay is justified by nanoparticles in AESO, which limit the movement of chains in the polymer bulk and increase the *T*_*trans*_. Further quantitative analysis on SMB is needed to elucidate the mechanisms governing the change in the *T*_*trans*_. In a recent study, researchers modified olive oil through epoxidation and acrylation, presenting a new oil-based SMP for tissue engineering scaffolds [91]. The acrylated olive oil showed a *T*_*trans*_ of 25.5 °C with a decreasing trend by adding acrylic acid to tune the hydrophobicity. The 4D-printed stent, composed of 25 % acrylic acid and acrylated olive oil, exhibited a transition temperature of 19.8 °C. The stent recovers at room temperature from subzero conditions (Fig. 3E) at 2.5 min, demonstrating potential for coronary artery interventions.

*Polyurethane Foams*: Shape memory polyurethanes (PUs) are of great interest due to their ease of processing, low material cost, adjustable *T*_*trans*_, and biocompatibility [92]. PUs can be synthesized via a straightforward addition reaction between an isocyanate and an alcohol containing at least two functional groups [93]. PU foams have been widely used in various biomedical applications, specifically for minimally invasive surgeries [92,94]. However, most developed PU foams rely on physical crosslinking, which may impose some limitations on their applications. For example, storing PU foams under compression at temperatures above their *T*_*trans*_ results in an irreversible deformation due to chain relaxation, termed “secondary shape forming”. One of the early studies overcame this problem by developing a chemically crosslinked foam using low molecular weight (MW) monomers [94]. They used N, N, N′, N′-tetrakis(2-hydroxypropyl) ethylenediamine (HPED), triethanolamine (TEA), and 1,6-diisocyanatehexane (HDI), forming an isocyanate premix. This premix reacts with polyol premix, including catalysts and surfactants, in a stoichiometric balance for at least one week. By varying the HPED and TEA ratios, they successfully tuned the *T*_*trans*_ and selectively set it within the range of 44–69 °C.

**Fig. 4:**
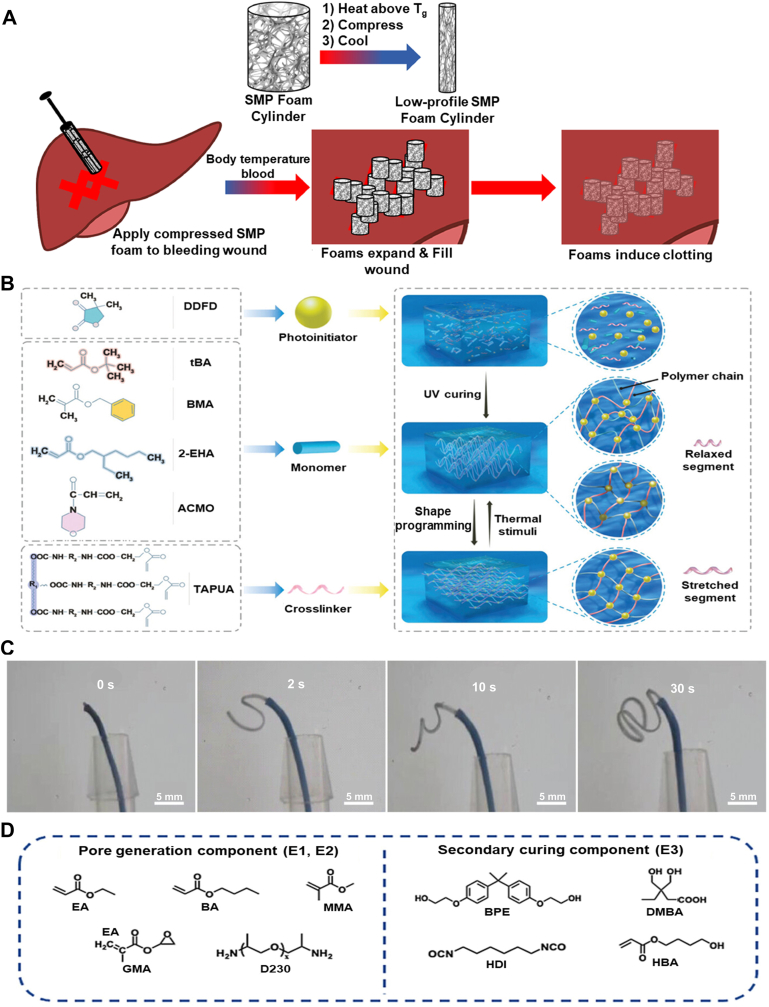
Chemically crosslinked TMPs with amorphous switching segments. A) The schematic of shape memory PU foam delivery shows the programming step by heating above their *T*_*g*_ (=*T*_*trans*_) and the subsequent cooling step to fix the temporary shape. Upon implanting compressed foams into the wound site, they expand to their primary shape, filling the wound and causing clot formation. Reproduced by permission [4]. Copyright 2022, Elsevier. B) Organogel components for temperature-triggered network design. The stretched network in programming can revert to its original shape (i.e., relaxed segments) at body temperature. Reproduced with permission [5]. Copyright 2023, Wiley-VCH. C) Shape change of organogel micro coils from a straight line to an omega-shaped structure within 30 s at 37 °C. Reproduced with permission [5]. Copyright 2023, Wiley-VCH. D) Three emulsion components for thermal and UV curing form networks with tunable properties by varying their concentrations. Reproduced with permission [6]. Copyright 2024, Elsevier.

PU foams have been successfully implemented in treating peripheral venous disorders, particularly for vascular occlusions [95]. Their high surface area enables rapid vessel occlusion while maintaining structural stability under flow conditions. These foams have also demonstrated promising performance in treating cerebral aneurysms, as they are biocompatible and facilitate rapid healing [96,97]. The initial clot formation within the foams facilitates the healing process, providing rapid endothelialization while minimizing inflammatory responses. An additional coating of n-vinylpyrrolidone and polyethylene glycol diacrylate hydrogel further enhanced the blood uptake of this foam [98]. This characteristic is also utilized in hemostatic wound dressings, where rapid absorption of blood and wound fluid is critical for effective healing. Upon exposure to physiological fluids at 37 °C, these foams were able to expand by over 1200 % within 15 min, showing excellent capability for treating deep and narrow wounds. In another study, PU foams could control hemorrhages from lethal wounds [48]. The resulting PU foam exhibits *T*_*trans*_ equal to 53 °C, which allows it to maintain its shape in high temperatures in a war zone. As illustrated in Fig. 4A, the foam is programmed to a compressed shape above its *T*_*trans*_ and is fixed by cooling. Upon contact with an injury site, it absorbs blood, reducing the *T*_*trans*_ to 25 °C, which enables it to expand and stop the bleeding at the injury site. This process occurs due to the disruption of hydrogen bonding, which leads to higher flexibility and free volume for polymer chains to move around. It is important to note that these PU foams are not considered WMPs, as their primary shape change is temperature-driven, despite their ability to interact with physiological fluid.

*Polyacrylate Networks*: Free radical photopolymerization is a widely employed technique for fabricating polymeric networks, with acrylate-based monomers playing a crucial role in this process [99]. Building on this strategy, a photocurable organogel formulation was developed using a photoinitiator, monomers, and a crosslinker [100]. The precursor solution was made using 4,4-dimethyldihydrofuran-2,3-dione (DDFD), tert-butyl acrylate (tBA), benzyl methacrylate (BMA), 2-ethylhexyl acrylate (2-EHA), 4-acryloylmorpholine (ACMO), and a trifunctional aliphatic polyurethane acrylate (TAPUA) as a crosslinker, as shown in Fig. 4B. This crosslinker was chosen among the different functional aliphatic PU acylates to tune the *T*_*trans*_ to match body temperature. A solution consisting of these components is then injected into a 3D-printed mold to fabricate micro-coils used as an aneurysm embolization device. The acrylate groups form the chemical netpoints, allowing the structure to maintain its integrity during deformation. After shape programming, the organogel network maintained a temporary shape with all segments stretched, as illustrated in Fig. 4B. Once triggered at body temperature, the network recovers to its permanent shape due to the stored entropy in the polymer chains. The shape recovery rate for this organogel is relatively high, demonstrating elastic behavior. The fabricated straight-line structure was transformed into an omega-shaped structure in less than 30 s, as evidenced in Fig. 4C. In another study, the same material system was used with the addition of magnetic particles to achieve dual-responsive behavior [101]. In this strategy, neodymium magnets enable the device to be implanted through a catheter and navigate tortuous vessels for embolization treatment. The device can be moved within the body and reach the treatment site by applying a magnetic field after implantation. Such dual-responsive functionality, which combines thermal shape recovery and magnetic movement actuation, expands the horizon of minimally invasive surgical techniques and offers enhanced precision and accessibility to internal anatomical sites via magnetic-guided systems.

In another study, polyacrylate chains were utilized to develop a TMP through a two-stage crosslinking strategy, resulting in a porous structure tailored for clinical applications [102]. Fig. 4D shows two emulsions, E1 and E2, each utilizing multiple acylate chains. These emulsions were composed of ethyl acrylate (EA), butyl acrylate (BA), methyl methacrylate (MMA), and glycidyl methacrylate (GMA). The PU emulsion, E3, was prepared based on the reaction between HDI and ethoxylated bisphenol A (BPE), acrylated with 4-hydroxybutyl acrylate (HBA) and two other emulsions, to form a mixture of emulsions capable of thermal and photocuring. Upon freezing and in the presence of poly(propylene glycol) bis(2-aminopropyl ether) (D230), the mixture formed networks through a reaction between epoxy and amino groups. A porous SMP can be obtained and shaped into a temporary form following a thermal treatment. This structure can be post-regulated by UV crosslinking of the double bonds. This step offers significant advancements in personalized medical treatment. A notable application of this technology is in earplugs, where conventional designs often cause pressure-induced discomfort or damage to the ear canal. By leveraging this two-stage crosslinking strategy, a compressed shape can be inserted into the ear, expanding to fit the unique anatomical structure before undergoing UV curing to achieve a second, permanent shape. This personalized fit effectively reduces pressure on the ear canal, enhancing comfort and functionality. The ratio between hard and soft acrylate components can be tuned to adjust *T*_*trans*_ to match body temperature, allowing precise control over mechanical properties.

#### 2.1.2 Chemically crosslinked semicrystalline TMPs (T_trans_ = T_m_)

*Glycerol-based Polyesters*: Polyesters are characterized by ester linkage formed via condensation reactions between alcohols and carboxylic acids [103]. The choice of monomers ultimately determines the outcome, allowing researchers to fine-tune the properties to meet their needs [103,104]. Among various polyester-based materials, glycerol-derived polyesters have gained significant attention due to their biocompatibility and minimal effect on metabolic pathways [104,105]. Among these, polyglycerol sebacate (PGS) and polyglycerol dodecanoate (PGD) have shown promising results for use as a TMP in minimally invasive surgery, with significant biocompatibility and tunable degradation properties [106,107]. Also, sebacic acid and dodecanedioic acid (DDA) are both resorbable upon degradation in vivo, making them great candidates for tissue engineering applications [105,107].

**Fig. 5:**
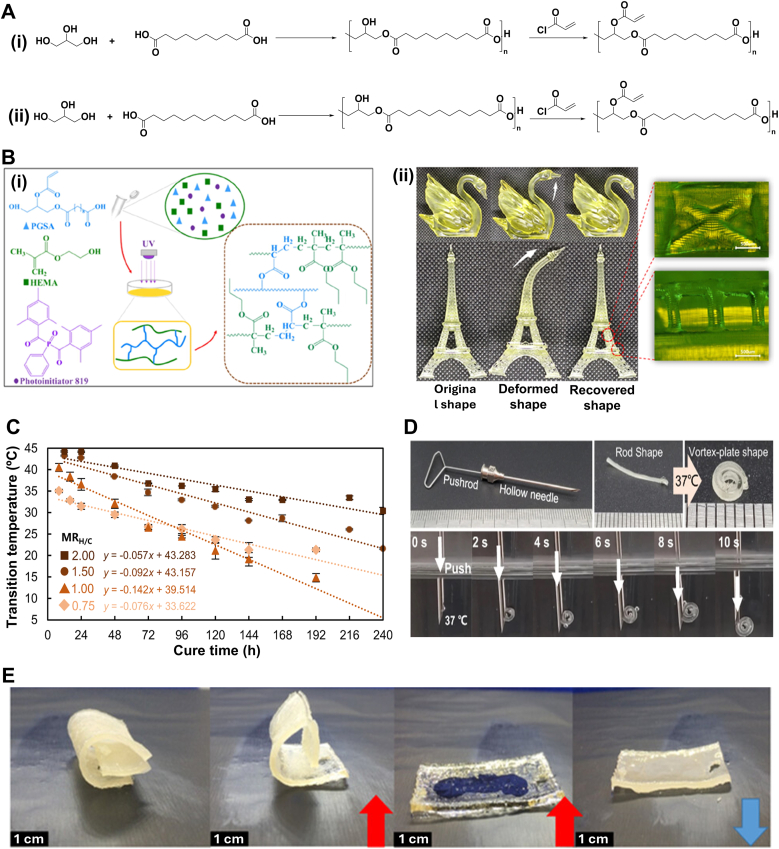
Glycerol-based TMPs synthesis and shape memory behavior. A) Schematic of the synthetic route for (i) PGS and (ii) PGD following the acrylation reaction using acryloyl chloride. B) PGSA-HEMA (i) ink formulation and schematic structures before and after printing, and (ii) its shape memory behavior at 37 °C. Reproduced with permission [7]. Copyright 2023, Elsevier. C) *T*_*trans*_ versus cure time for different MR_H/C_ of PGD obtained from differential scanning calorimetry. Reproduced under terms of the CC-BY license [8]. Copyright 2023, The Authors, published by Springer Nature. D) Shape recovery of programmed PGD from rod shape to vortex-plate shape at 37 °C with MR_H/C_ = 1.5 and a curing time of 72 h. Reproduced under terms of the CC-BY license [8]. Copyright 2023, The Authors, published by Springer Nature. E) Shape change for a programmed PGDA sample upon heating to its *T*_*trans*_. Reproduced with permission [9]. Copyright 2021, Wiley-VCH.

Both PGS and PGD are elastomeric polyesters with strain-stiffening behavior, making them suitable for soft tissue implants [68,107]. Their synthesis mechanisms are similar, requiring high-temperature processing under an inert atmosphere or catalytic-assisted reactions at lower temperatures, as shown in Fig. 5A [37,107]. Depending on the initial monomer ratios, the existing functional groups on the polymer chains can form chemical netpoints under vacuum conditions at elevated temperatures [105,107,108]. Furthermore, their thermomechanical properties can be fine-tuned via copolymerization or by introducing functional moieties to the structure [107,109]. PGS has a low *T*_*trans*_ (between −10 °C and 10 °C), which creates problems during storage and recovery, since the temperature is below body temperature [110]. To address this issue, stearic acid, a fatty acid derivative, has been incorporated into PSG to match *T*_*trans*_ with body temperature, showing a recovery rate of 85 % at 37 °C [110]. Despite this improvement, further research is necessary to comprehensively evaluate the effects of this modification on both mechanical and biological properties. As the shape memory property originates from the crystalline structure of sebacic acid, using bifunctional alcohol instead of glycerol leads to a linear polymer with higher regularity and an increased *T*_*trans*_, which is attributed to the formation of more crystallites [111]. Using this approach, polypropylene sebacate (PPS) was synthesized using 1,3-propanediol [111]. Blending PPS (as switching segments) into the PGS results in an elastic material system. While the *T*_*trans*_ of this structure is 45 °C, introducing Kartogenin to the chemical makeup can reduce it to about body temperature, with *R*_*r*_ and *R*_*f*_ values of 98 %. Theoretically, different molar ratios of alcohols can engineer a body-responsive TMP.

A recent study used a mixture of PGS acrylate (PGSA) and hydroxyethyl methacrylate (HEMA) to increase *T*_*trans*_ [112]. Firstly, the synthesized PGS was modified using acryloyl chloride to substitute the remaining hydroxyl groups with a UV crosslinkable moiety (Fig. 5A(i)). Subsequently, HEMA was introduced to incorporate hard segments within the soft segments of the PGSA, resulting in a higher *T*_*trans*_ (≈37 °C) suitable for body-responsive devices. As illustrated in Fig. 5B(i-ii), HEMA has been well incorporated into the structure, resulting in increased *R*_*f*_ and *R*_*r*_ (>90 %). Introducing HEMA hard segments to PGSA reduced its elasticity, which demonstrates the tunability of mechanical properties for the target applications. Due to the pendant hydroxyl group of HEMA, the networks exhibited a higher degradation rate and hydrophilicity, demonstrating their potential as scaffolds for tissue engineering. Although multiple studies on PGS-based biomaterials have yielded significant results, the SMB of this polymer has not been well studied to date [107]. Adding similar materials (such as stearyl and lauryl acrylates [113,114]) could yield new TMPs designs for biomedical applications.

The other glycerol-based polyester, PGD, was first synthesized in 2009 for tissue engineering applications [105]. By extending the chain length of the carboxylic acid to ten carbons using DDA instead of sebacic acid in PGS, an elastomeric network was synthesized with a *T*_*trans*_ of 30–34 °C via controlling the crosslinking density. The un-crosslinked PGD has a *T*_*trans*_ of 45 °C [105,108,115]. It has been reported that adjusting the molar ratio of glycerol to DDA (MR_H/C_) in the range of 0.75–2 and controlling the curing time from 24 to 240 h leads to achieving a *T*_*trans*_ ranging 15–45 °C, as shown by differential scanning calorimetry (Fig. 5C) [49]. Increasing the curing time led to a decreased *T*_*trans*_, which is attributed to a larger number of netpoints between the chains [49,108,116]. The greater number of netpoints constrained the polymer chains’ mobility, resulting in less crystalline domain formation. The structure with an MR_H/C_ of 1.5 and a 72-h curing time, shown in Fig. 5D, exhibited rapid recovery at body temperature [49]. Moreover, this TMP offers a nonlinear elastic behavior at body temperature, making it a great candidate for soft tissue repair. The increase in the crosslinking density also improves the nonlinear behavior of the body-responsive TMP without any adverse effects on its biocompatibility [117].

Despite the promising properties of PGD, thermal curing of PGD hinders its application in clinical settings, as the harsh synthesis conditions limit the incorporation of therapeutics into the material system [118,119]. To circumvent this issue, researchers employed acrylation chemistry, a widely used modification strategy, to graft acrylate groups onto the chain, thereby creating a photocrosslinkable polymer [115,118]. Similar to the acrylation of PGS, acryloyl chloride is utilized to substitute the existing hydroxyl groups in PGD with acrylates to form acrylated PGD (PGDA, Fig. 5A(ii)) [115]. The acrylation percentage subsequently affects the photocrosslinking kinetics, crosslinking density, and mechanical properties. This resulted in a *T*_*trans*_ range from 20 °C to 37 °C, enabling shape recovery within body temperature, as shown in Fig. 5E [115,118]. The difference between the *T*_*trans*_ range for PGD and PGDA originates from the acrylate side groups, which impede crystal formation at the molecular level in PGDA by disrupting chain regularity [118]. In general, acrylation can potentially compromise biocompatibility by introducing toxic groups to the polymer; thus, setting a threshold for the acrylation percentage is crucial for biomedical applications.

*Polycaprolactone*: In the polyester-based TMPs category, PCL has been extensively studied due to its excellent biocompatibility and viscoelastic properties [50,103]. PCL can be formed either directly or indirectly via the polycondensation of 6-hydroxyhexanoic acid or the ring-opening polymerization of ε-caprolactone, primarily in the presence of Sn(Oct)_2_ as the catalyst and an alcohol as the initiator [103,120]. While polycondensation is a more straightforward process, it has been less prevalent due to its tendency to produce a lower MW and a higher dispersity index [120].

**Fig. 6:**
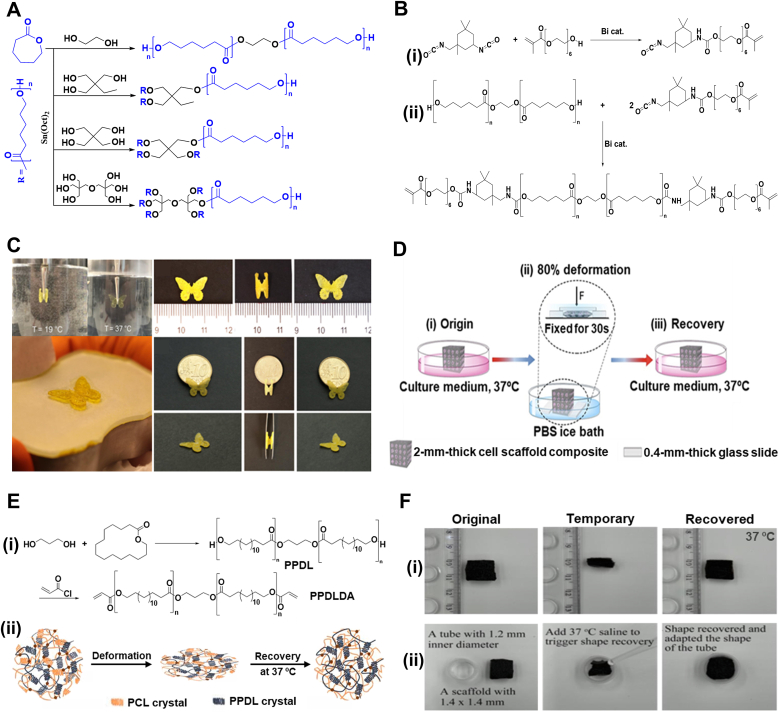
Synthesis and shape memory performance of PCL-based chemically crosslinked TMPs. A) Schematic of the synthetic route for linear and nonlinear PCL synthesis. B) Reaction mechanism of (i) the reaction between isophorone diisocyanate and polyethyleneglycol monoacrylate to obtain an acrylate-isocyanate functionalized reagent, and (ii) a diacrylate PCL obtained from the reaction between PCL diol (obtained through a ring-opening polymerization of ε-caprolactone using ethylene glycol as an initiator) and acrylate-isocyanate reagent, both in toluene with bismuth neodecanoate acting as a catalyst. C) Shape recovery of a 4D-printed butterfly made of acrylated PCL at 37 °C. No shape change was observed at 19 °C. Reproduced with permission [10]. Copyright 2023, Elsevier. D) The schematic of a cell-laden PGA and PCL porous network at (i) original shape, during (ii) programming in an ice bath with 80 % deformation, and (iii) recovery at 37 °C. Reproduced with permission [11]. Copyright 2018, Royal Society of Chemistry. E) PPDL (i) synthesis route using propanediol and ω-pentadecalactone and its incorporation into PGA-g-PCL for enhanced mechanical properties through reinforcement of PPDL and shape recovery at 37 °C through PCL crystals as switching segments. Reproduced under terms of the CC-BY license [12]. Copyright 2023, The Authors, published by KeAi Chinese Roots Global Impact. F) PPDL-reinforced network (i) shape recovery and (ii) adaptability upon triggering at 37 °C in air and saline, respectively. Reproduced under terms of the CC-BY license [12]. Copyright 2023, The Authors, published by KeAi Chinese Roots Global Impact.

Biodegradable PCL-based TMPs have been studied for various biomedical applications ranging from sutures to stents [50,53]. However, the high *T*_*trans*_ introduced by crystalline regions raised concerns about utilizing them as body-responsive TMPs. This problem has been addressed by developing different synthetic approaches. MW adjustment is one straightforward approach to tuning the shape memory performance of PCL [53,55]. It has been reported that the higher MW of PCL leads to a higher crystallinity percentage, which, in turn, exhibits better shape memory performance but at a higher *T*_*trans*_ than body temperature [53,121]. Linear acrylated PCLs, with an MW of 5000–10000 g mol^−1^, showed a *T*_*trans*_ of about 20 °C above body temperature, making them non-responsive to physiological temperature [50]. Utilizing external heating to trigger the shape change would damage the surrounding tissues, rendering these TMPs an unviable option for body-responsive implants. Lower MW PCLs also exhibited a similar trend with *T*_*trans*_ between 40 °C and 60 °C [121]. Tuning the *T*_*trans*_ through MW seems an unfeasible approach; thus, manipulating the polymer architecture was considered as an alternative [50,122,123]. As previously discussed, regularity eases the alignment of polymer chains to form crystalline regions. Decreasing the PCL linearity can disrupt segments alignment and crystal formation, resulting in lower *T*_*trans*_. This result can be achieved by changing the initiators from bifunctional reagents to multifunctional ones to form non-linear structures, as illustrated in Fig. 6A [50,124]. The induced arms form a star-PCL with a higher free volume between the chains, thereby easing their movements and reducing the alignment. This free volume can also improve the PCL's biodegradability rate, which would eliminate the need to remove PCL-based devices [50,122]. Due to the length of the arms, the four-armed PCL, regardless of MW, has shown more significant changes in *T*_*trans*_ compared to the linear PCL [50]. While multi-armed PCLs have demonstrated outstanding potential in balancing the crystallinity and SMB, further research on higher-order branched PCL architectures remains limited, highlighting an area for future work in body-responsive TMPs.

**Fig. 7:**
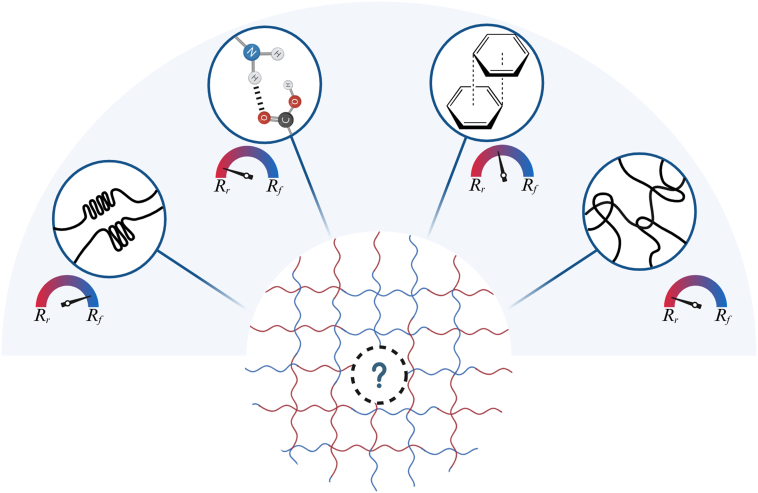
Physical crosslinks for TMP networks. Crystalline regions, hydrogen bonding, π-π stacking, and chain entanglements (left to right) are the most common physical netpoints in designing TMPs. Except for crystalline domains, they can all increase the recovery stage of the TMPs.

Because of the existing hydroxyl groups on its chains, PCL can be modified using various moieties [55]. As depicted in Fig. 6A, linear PCL was synthesized through a ring-opening reaction in the presence of ethylene glycol as an initiator under the catalysis of Sn(Oct)_2_. To achieve photocrosslinkable PCL, hydroxyl groups were further reacted with acrylate reagents synthesized by reacting isophorone diisocyanate with polyethylene glycol monoacrylate, as schemed in Fig. 6B(i–ii). The acrylated PCL with a MW of 2000 g mol^−1^ showed a *T*_*trans*_ of 35.2 °C, approximately 12 °C lower than the transition temperature of unmodified PCL. The addition of acrylate groups disrupted the formation of crystal lattices and promoted chain mobility, which in turn resulted in a lower *T*_*trans*._ As shown in Fig. 6C, the 4D-printed butterfly made of photocurable PCL could quickly revert to its original shape upon heating to 37 °C. These modifications enable PCL to integrate with other polymers, introducing a tunable crystalline region that enhances shape memory behavior in designing body-responsive SMPs.

Polypeptides are known for their biocompatibility and biodegradability in organ regeneration [121,125]. Providing shape memory scaffolds with a polypeptide-based network can, therefore, facilitate their use in biomedical applications. One approach is to covalently link poly (L-glutamic acid) (PGA) via an esterification reaction with the existing hydroxyl groups on the PCL chains. For this purpose, PGA was prepared using the ring-opening polymerization of N-carboxyanhydride in *γ*-benzyl-L-glutamate [121,126]. PCL has also been prepared by the reaction between ɛ-caprolactone and 1,3-propanediol at 120 °C [121]. PCLs with different MWs were crosslinked with PGA using varying feed ratios via a solvent particulate leaching technique to fabricate a porous structure. The *T*_*trans*_ of the fabricated structures showed that their shape memory behavior is due to the synergistic effects of the PCL's MW and its crosslinking density. Increasing the crosslinking density from 30 % to 90 % increases the crystallinity by about 50 %. This led to an increase in network *T*_*trans*_ due to the formation of more uniform crystals, as evidenced by the differential scanning calorimetry results [121]. This can also be justified by forming more netpoints that hold the crystal more compactly in their regions. The high-MW network (∼3500 g mol^−1^) with 60 % crosslinking density showed a *T*_*trans*_ value of 35 °C, suitable for body responsiveness. It exhibited excellent recovery in culture media upon 80 % deformation (Fig. 6D). In a similar study, methacrylate PCL with the same MW was grafted onto PGA to achieve a photocrosslinkable polymer [54], which could then be used in any reaction with different acrylated materials. Poly(ω-pentadecalactone) diacrylate (PPDLDA), synthesized from 1,3-propanediol and ω-pentadecalactone, was used to create a more mechanically stable structure by introducing crystalline regions that are stable at body temperature, as shown in Fig. 6E [54]. With this adjustment, the scaffold demonstrated robust mechanical properties, enabling it to be used in applications with a high number of loading cycles, such as dental implants. Different mass ratios of PGA-g-PCL and PPDLDA resulted in changes in *T*_*trans*_. This result is attributed to the easier formation of crystals when PPDLDA is incorporated in smaller amounts. The 70:30 and 50:50 mass ratios of PPDLDA to PCL showed *T*_trans_ values of 36 °C and 37 °C, respectively. As demonstrated in Fig. 6F, the scaffold exhibited self-adaptive expansion, making it suitable for filling irregular defects in the body, such as bone fractures.

### 2.2 Physically crosslinked body-responsive TMPs

Physically crosslinked TMPs are typically designed with hard and soft segments, where hard segments act as netpoints and soft segments function as switching domains. Polymers based on lactide, lactone, and urethane chemistries have been utilized to design this category of TMPs. Although chemically-crosslinked TMPs provided better mechanical properties and sharper recovery at body temperature range, their fixed covalent bonds could weaken their shape programming and hinder their reprocessing [76,77,127]. The non-covalent interactions between these segments provide the structure with movement and reversibility, potentially leading to lower cytotoxicity due to the absence of toxic moieties, such as acylates. These dynamic structures, which incorporate crystalline domains, hydrogen bonding, π-π stacking, and chain entanglements, are illustrated in Fig. 7. Except for crystalline domains, which comprise compact structures, the dynamic nature of these bonds facilitates the recovery of physically crosslinked TMPs. Generally, these movable netpoints offer a great range of motion during recovery, leading to higher *R*_*r*_. Unlike covalent netpoints, these structures are free of toxic moieties, such as acrylates, which potentially lead to lower cytotoxicity. These interactions can also be further exploited to design self-healing TMP constructs due to their ability to restore their bonding upon damage. In contrast, these domains reduce the *R*_*f*_ of the networks compared to the chemically crosslinked TMPs, as physical netpoints are weaker and more prone to netpoints movement. These physical domain interactions can be incorporated into the TMPs to achieve synergistic effects, as they are likely to be insufficient for shape fixity solely. In this section, we review the TMPs that have physical interactions as their netpoints.

#### 2.2.1 Physically crosslinked amorphous TMPs (T_trans_ = T_g_)

*Polyester/copolyester blends*: Many polymers with favorable biological properties cannot be directly used as body-responsive TMPs without modification, mainly due to high *T*_*trans*_, brittleness, or biodegradation rates [37]. For instance, PCL and PLA are two medically well-known polymers with limited biomedical applications as body-responsive TMPs. Physical blending is a straightforward approach for tuning different polymer properties, such as *T*_*trans*_ [[128], [129], [130]]. In particular, melt blending PLA with low-MW polyethylene glycol (PEG) has been used to produce filaments with a *T*_*trans*_ of 38.2 °C for 3D printing. Similarly, blending PCL with PUs can reduce the *T*_*m*_ value of PCL crystallites, enhancing the suitability of TMPs for biomedical applications. Additionally, blending of PLLA-PTMC and polyglycolic acid-PTMC showed *T*_*trans*_ tunability to body temperature between 29.43 °C and 26.74 °C, allowing body-responsiveness. However, these studies did not include quantitative analyses of *R*_*f*_ and *R*_*r*_, which are essential for evaluating the SMB of the polymer network.

Copolymerization is a promising, versatile approach to tailoring polymer properties for specific application requirements [131]. A tunable multiblock copolymer using PLLA and poly(glycolide-co-caprolactone) (PGCL) macrodiol was synthesized using Sn(Oct)_2_ as the catalyst and ethylene glycol as the initiator [131]. The PLLA polymer is the hard segment of the blend and constitutes a physical network of crystalline domains, while PGCL, which is amorphous at body temperature, is considered the soft segment. The results showed that a *T*_*trans*_ of below 40 °C is achievable by maintaining the MW of both copolymer blocks close to 2000 g mol^−1^ through monomer–initiator ratio adjustment [131]. The copolymer with a 20:80 block weight ratio had a *T*_*trans*_ value of 40.9 °C, which was caused by stiffer chains. Higher PLLA content increases the number of crystalline netpoints in the SMP network, elevating the *R*_*f*_ to 95 %. In contrast, higher PGCL content increased the amorphous content, resulting in a higher *R*_*r*_. It was also evident that repeating the programming and recovery cycle increased *R*_*f*_ and *R*_*r*_. The increased *R*_*r*_ is attributed to the residual stress remaining in the structure from previous cycles, leading to a faster response to temperature. Given its tunability and high shape fixity, this copolymer system could be envisioned for future biomedical applications, such as biodegradable self-tightening sutures, where precise mechanical adaptability and controlled degradation are essential [132].

**Fig. 8:**
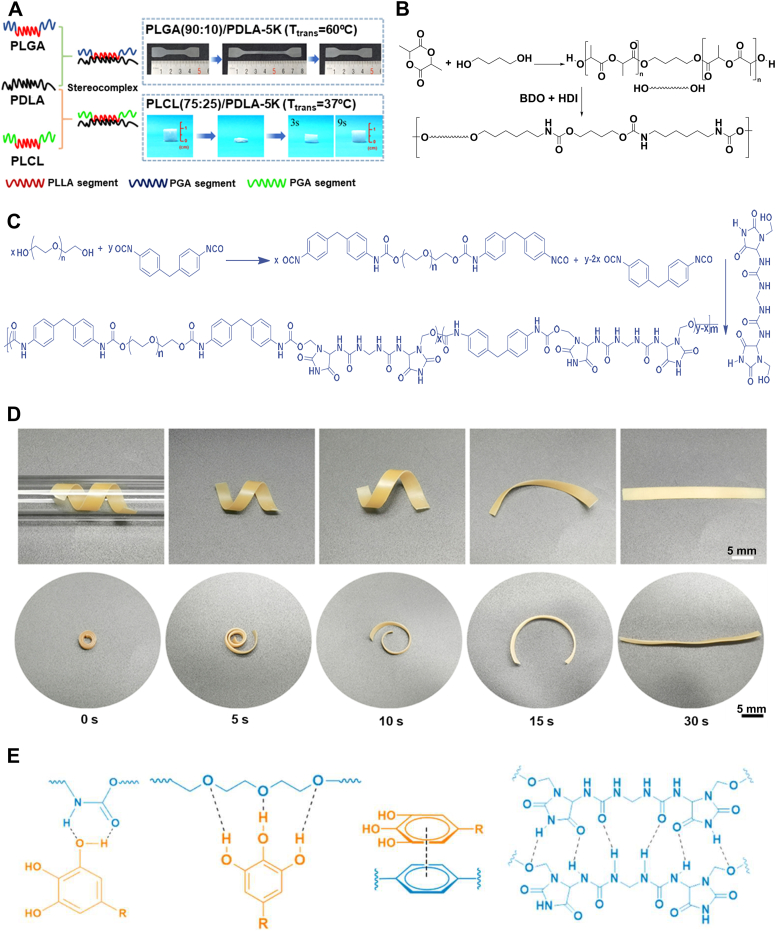
Synthesis and shape memory performance of physically crosslinked TMPs with polyester as their soft segments. A) Stereo complexity and shape recovery properties of a PDLA blend with PLGA and PLCL at distinct *T*_*trans*_. Reproduced with permission [13]. Copyright 2019, American Chemical Society. B) Schematic of the synthetic route for polylactide PU TMP using L-lactic acid and BDO forming the soft segment in the presence of Sn(Oct)_2_ to further react with HDI and BDO forming the urethane linkage. C) Schematic of the synthetic route for ultra-durable PU TMP out of PEG, MDI, and IU in the presence of dibutyltin dilaurate as a catalyst. Reproduced under terms of the CC-BY license [14]. Copyright 2023, The Authors, Published by Springer Nature. D) Photographs of shape recovery of a helix-shaped structure deployed via an 8-mm diameter glass tube to a 37 °C environment (scale bar: 5 mm). Reproduced under terms of the CC-BY license [14]. Copyright 2023, The Authors, Published by Springer Nature. E) Possible non-covalent interactions between PU, TA, and KGN. Examples of these non-covalent bonds include hydrogen bonding and π-π stacking. Reproduced under terms of the CC-BY license [14]. Copyright 2023, The Authors, Published by Springer Nature.

Designing body-responsive TMPs with the required toughness and elasticity is challenging [131,132]. For instance, PGCL/PLLA copolymers with acceptable shape recovery properties lack sufficient mechanical properties at body temperature. The addition of caprolactone as a comonomer has improved these properties [133]. A blend of poly(D-lactide) (PDLA) and poly(lactide-co-caprolactone) (PLCL) was prepared, with a 3:1 ratio of lactide to caprolactone. The low *T*_*g*_ value of PCL domains incorporated into PGCL copolymer is the main reason for reducing *T*_*trans*_ to body temperature. Additionally, blending PDLA with PGCL instead of PLLA resulted in higher shape fixity. This improvement was achieved because unique crystalline domains formed in the fixed region, described as a *stereo-complex* PLA, as shown in Fig. 8A. The higher number of netpoints after the addition of PDLA increased *R*_*r*_ to 96.5 %, compared to 92.2 % for pristine PLCL. In another copolymerization approach, a poly(lactide-co-glycolic) acid (PLGA) and PTMC copolymer was synthesized in the presence of a zirconium acetylacetonate catalyst, rather than the commonly used Sn(Oct)_2_, due to its ability to be excreted through metabolic pathways, thereby reducing the risk of cytotoxicity [134]. The PLGA-PTMC polymer's *T*_*trans*_ of 39 °C originates from its chain entanglements, showing an *R*_*r*_ of 90 % at body temperature. Decreasing the TMC content and using the zirconium catalyst improved the recovery ratio to 95 %. This finding is justified by a relatively higher density of entanglements, leading to increased latent thermal energy and faster recovery. PTMC with higher lactide content was then synthesized to fabricate a robust porous TMP scaffold for bone regeneration [135]. The higher lactide content promoted the formation of crystalline netpoints, while carbonate blocks served as switching segments, resulting in an *R*_*r*_ of 94 %. These findings demonstrate the potential of lactide-enriched PTMC networks for biomedical applications that require durable and efficient memory behavior.

*Polyurethane/Polyurea with polyester soft segment:* Polyurethanes contain isocyanate *hard* segments, which can form crystals by strong hydrogen bonding between amine groups. In contrast, their soft segments can deform due to their amorphous structure [[136], [137], [138], [139], [140]]. *Microphase separation in PUs is the reason behind the emergence of physically crosslinked PU-based TMPs.* [141] The separation is between the hard and soft segments of PU, acting as the stationary and switching segments, respectively. Although designing PU TMPs is versatile, due to an extensive catalog of monomers, their toxicity and biodegradation properties hinder their capability in the biomedical field. Additionally, lowering *T*_*trans*_, which is dictated by soft segments, leads to insufficient mechanical properties under physiological conditions. The mechanical properties can be adjusted using polyesters, such as PLA and PCL, as soft segments. Tailoring the ratio of soft to hard *segments and the* polyester segment length is a key factor in *R*_*r*_ and *R*_*f*_ of the TMPs. The PLA diols incorporated into the PU structure, which can be seen in Fig. 8B, provided a *T*_*trans*_ of 33 °C and compensated for the low mechanical properties of typical PUs created from HDI and 1,4-butanediol (BDO) [140]. It shows that while the increase in hard segment content reduces the *R*_*r*_ from 100 % to 96 % due to increased movement hindrance*,* it increases *R*_*f*_. In a similar study, the addition of urea groups increased the hydrogen bond density, which resulted in an *R*_*f*_ value of 100 % *and preserved it even* after three recovery cycles [142]. It was later reported that incorporating a *cyclic* piperazine chain extender enhances the hard segment strength due to its six-atom rigid structure [143,144]. Piperazine increased the soft segment length compared to a typical shape memory PU, improving the *R*_*r*_ from 88.7 % to 98.6 %. Moreover, the amplified physical interaction in hard segments improved the *R*_*f*_ from 84.6 % to 95.3 %, with a *T*_*trans*_ equal to 36 °C. To further enhance the mechanical properties without compromising SMB, researchers used isosorbide (ISO) as the diisocyanate chain extender, creating a more robust structure [144]. Although the resulting TMP's *T*_*trans*_ reached 43.5 °C, which is higher than that for HDI-based PU's, it could be further tuned to achieve a body-responsive TMP. This can be achieved by decreasing the ISO-to-HDI ratio, since hard segments of ISO can decrease chain mobility and lead to a higher value for *T*_*trans*_.

Developing an ultra-durable hydrogel network based on PU TMPs has significantly advanced the design of body-responsive TMPs [51]. As shown in Fig. 8C, the polymer is synthesized through the chemical reaction between 4,4-methylenediphenyl diisocyanate (MDI), imidazoline urea (IU), and PEG from a robust network capable of being reinforced by hydrogen bonding through water swelling. First, MDI and PEG react, forming a diisocyanate prepolymer. This is followed by a reaction between the hydroxyl of IU and the existing isocyanates, forming urethane linkages using dibutyltin dilaurate as a catalyst. Introducing small molecules, such as Kartogenin or tannic acid (TA), to the TMP further reinforced the structure by providing additional hydrogen bonding sites. Fig. 8D illustrates possible non-covalent interactions between TA, Kartogenin, and IU, mediated by hydrogen bonding or π-π stacking. These small molecules enhance mechanical properties and provide anti-inflammatory and antioxidant functionalities [51]. The scaffold can exhibit rapid temperature responsiveness due to the reinforced dynamic structure by multiple non-covalent interactions. As shown in Fig. 8E, the temporary helix shape was fixed at 4 °C and subsequently transferred through an 8-mm diameter glass tube. The structure was able to recover its original shape when heated to 37 °C. The same rapid shape recovery has also been observed for the coil-shaped structure, which was reported to maintain its recovery ratio at around 90 % after nine successive shape-fixing and recovery cycles.

#### 2.2.2 Physically crosslinked semicrystalline TMPs (T_trans_ = T_m_)

**Fig. 9:**
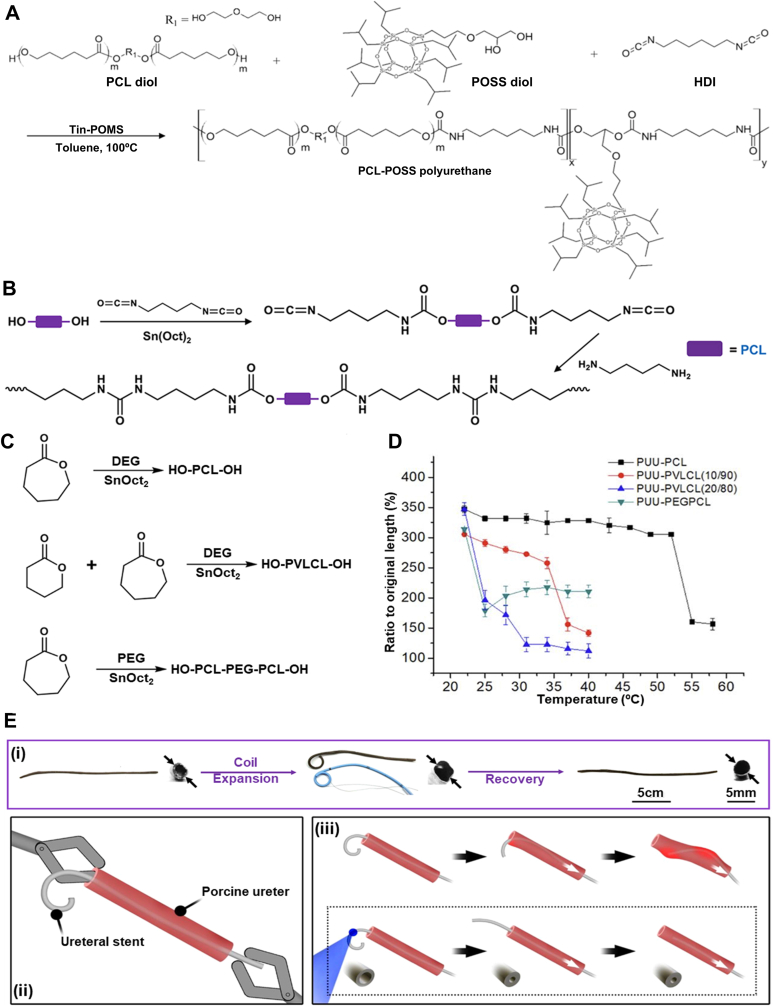
Synthesis and shape memory properties of PCL-based physically crosslinked TMPs. A) Schematic of the synthetic route for the copolymerization of PCL with POSS using HDI as a chain extender to introduce urethane linkage to the structure as physical crosslinks. Reproduced with permission [15].Copyright 2016, American Chemical Society. B) Synthetic route for creating PUU-PCL networks using 1,4-diisocyanatobutane in the presence of Sn(Oct)_2_, where PCL acts as a switching segment [16]. Copyright 2024, The Authors, Published by Springer Nature. C) Schematic of synthetic routes for different soft segments: PCL diol, PVLCL diol, and PCL-PEG-PCL diol used in PUUs synthesis [16]. Copyright 2024, The Authors, Published by Springer Nature. D) *T*_*trans*_ of PUUs copolymerized with VL and PEG to lower *T*_*trans*_ to below body temperature [16]. Copyright 2024, The Authors, Published by Springer Nature. E) Shape recovery of (i) Fe_3_O_4_-incorporated PUU-PCL coiled stent expanded via a balloon and recovered to its unfolded shape upon heating. (ii) Scheme of the stent removal procedure from an ex vivo porcine ureter model and (iii) its resistance compared with commercially available stents (top), which cause damage to the tissue. Reproduced under terms of the CC-BY license [16]. Copyright 2024, The Authors, Published by Springer Nature.

*Network with PCL as soft segment: As discussed previously, the high transition temperature for most pristine polyesters limits their usefulness in designing body-responsive SMPs.* [50,145,146] *However, in some cases like PCL and PEG, the crystalline melting temperature is low enough that it can be utilized as a T*_*trans*_ *and adjusted to the desired temperature. In addition to previous strategies, introducing bulky and hard polyhedral oligomeric silsesquioxane (POSS) segments can also fine-tune T*_*trans*_ [145]. As shown in Fig. 9A, the PCL diol (1000 g mol^−1^) is linked to hydroxyl-modified POSS with HDI in the presence of tin-POMS, forming POSS polyurethane. The addition of POSS induces disruptions in PCL crystallization, reducing the *T*_*trans*_ to the body temperature. Although POSS could decrease the *T*_*trans*_, its bulky chemical makeup hinders the recovery and decreases *R*_*r*_. In a similar study, PCL copolymerized with polydimethylsiloxane (PDMS) exhibited a *T*_*trans*_ of 37 °C [147]. It was reported that, although *T*_*trans*_ is constant for all block ratios, higher PCL leads to more crystallinity, which improves the Rf while enabling the structure to preserve its temporary shape more effectively. Despite the low cell viability, this network has shown strong potential for use as an emergency dressing for acute open wounds.

Controlling the biodegradation of PCL networks has been challenging in designing tissue-specific structures [[148], [149], [150]]. In a recent study, δ-valerolactone (VL) was incorporated into a PCL network to introduce more degradable ester bonds into the structure, while also reducing crystallinity, resulting in a higher degradation rate [151]. This strategy enables tunability for developing PCL-based constructs that respond to physiological conditions. As shown in Fig. 9B, polyurethane urea (PUU) copolymers form through the reaction of diols with 1,4-diisocyanatobutane. The reaction continues with diamine to create urea linkages. This creates segmented PUU networks whose thermomechanical properties depend on the specific diol used. The diols incorporated into this PUU structure, such as PCL, PVLCL, or PCL-PEG-PCL, are prepared through ring-opening polymerization of VL and ε-caprolactone using diethylene glycol (DEG) or PEG as the initiator and Sn(Oct)_2_ as the catalyst (Fig. 9C). These diols differ in composition, crystallinity, and hydrophilicity, which allows the resulting PUUs to exhibit tailored degradation behavior and thermal responsiveness.

As shown in Fig. 9D, the transition temperature of PUUs changes when copolymerized with PEG and VL [151]. *T*_*trans*_ was measured when the samples began shrinking upon immersion in 20 °C water. The fabricated stent showed an *R*_*r*_ of 87 % with a *T*_*trans*_ of 35 °C when the VL content of the copolymer was 10 %. Notably, the stent developed in this work was not a body-responsive implant, as it was designed as a double-J ureteral stent, facilitating its removal from the kidney with minimal patient discomfort. However, the addition of Fe_3_O_4_ nanoparticles in the PUU-PCL network resulted in a smart stent triggered with high-intensity focused ultrasound. Fig. 9E(i) shows that the single-J stent, which has a similar coil as that for the commercial counterpart, could swiftly recover to its straight shape within 3 s using indirect heating. The illustration of ex vivo stent removal from a porcine ureter is shown in Fig. 9E(ii). While removing the commercial urethral stent may cause tissue damage during removal, as depicted in Fig. 9E(iii), the novel TMP-based coil can minimize the risk of damage to the surrounding tissue. Multiple hydrogen bonds within the polymer network, formed between urea groups, serve as physical crosslinks, while PCL acts as a switching segment. This approach has potential for the design of removable biomedical implants with body responsiveness. Ultrasound-based activation strategy could also be used as a controllable actuation mechanism for shape memory implants.

**Fig. 10:**
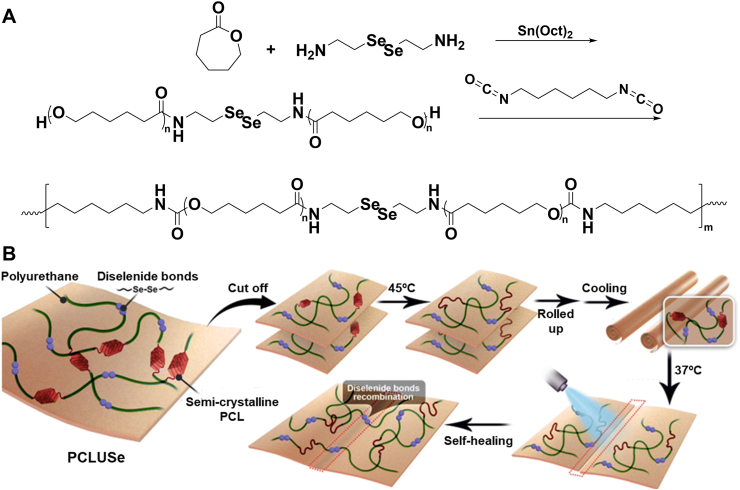
Synthesis and shape memory behavior of physically crosslinked TMPs with crystalline switching segments. A) Synthetic route for PCLUSe from ring-opening polymerization of ɛ-caprolactone initiated by selenocystamine in the presence of Sn(Oct)_2_. The prepolymer then reacts with 1,6-diisocyanatehexane, forming PCLUSe. B) Shape memory and self-healing performance of degradable PCLUSe at body temperature under 405-nm irradiation. Reproduced with permission [17]. Copyright 2023, Royal Society of Chemistry.

Self-healing moieties can be incorporated into TMPs to fabricate shape-changing implants with larger dimensions for minimally invasive procedures [152]. In a novel approach for PCL copolymers, illustrated in Fig. 10A, selenocystamine was *coupled* with caprolactone to introduce dynamic diselenide bonds into polycaprolactone. Diselenide-hydroxyl macro-PCL reacted with HDI to create PCL selenocystamine-based PU (PCLUSe). The PCLUSe polymer sheets can be cut into pieces, rolled into a temporary shape, and delivered to the body using a catheter-based technique. Upon exposure to body temperature, the implanted sheets restored their original shape and spliced together through the formation of weak diselenide bonds induced by 405-nm irradiation (Fig. 10B). The diselenide bonds reduced *T*_*trans*_ by disturbing the PCL crystallization while improving the mechanical properties. The synthesized PCLUSe showed an *R*_*r*_ of 86.5 %, which increased to 98.6 % after multiple shape recovery cycles. In contrast, the *R*_*f*_ remained constant at 99.6 % for all samples. The gradual improvement in recovery ratio through shape memory cycles was attributed to the high stress relaxation of diselenide bonds, which aligned in newly formed entanglements in the direction of stress.

**Fig. 11:**
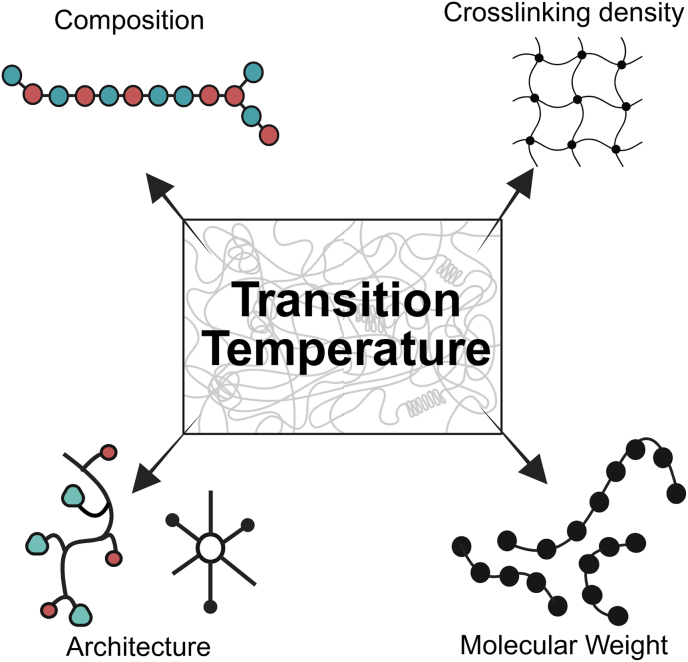
Illustration of different parameters, including composition, architecture, molecular weight, and crosslinking density, affecting *T*_*trans*_.

**Fig. 12:**
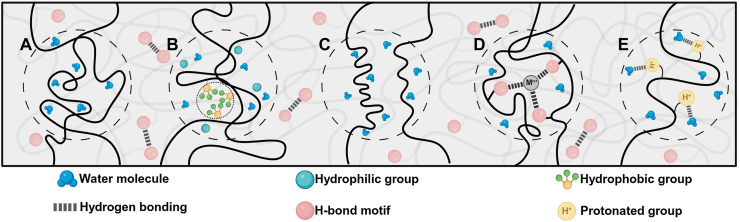
Illustration of different switching segments of WMPs under wet conditions. Upon water immersion, A) amorphous segments are plasticized by water molecules that penetrate the structure, B) hydrophobic blocks within the structure act as the stationary segment through aggregation, and C) hydrophilic crystalline domains are solvated through the penetration of water molecules. Introduction of charges in an aqueous environment using D) metal cations and E) protons to create new hydrogen bonds, enabling the structure to change shape. Cations and acidic environments within the body can facilitate this process. The H-bond motifs exist throughout the structure of the WMPs; however, due to the simplicity of the illustration, we only focused on switching segments.

*Non-PCL copolymers:* Incorporating PCL soft segments to design physically crosslinked TMPs has been widely studied. However, new copolymers have been recently developed that do not incorporate PCL but have biocompatibility and shape memory performance with a crystalline *T*_*trans*_ that matches body temperature [[153], [154], [155]]. Conjugation of polyethylene glycol methacrylate (PEGMA) to a polysulfone backbone introduced body-responsiveness to the network, owing to PEGMA crystalline regions serving as the switching segment [153]. Increasing the PEGMA content of the copolymer improves the micro-phase separation between hard and soft segments. TMPs with 73 % PEGMA show an *R*_*r*_ of 99 %, but a higher MW of PEGMA would compromise shape recovery. Using a similar approach, researchers synthesized a block copolymer TMP from tert-butyl acrylate and PEGMA [154]. The resulting TMP was body-responsive (i.e., *T*_*trans*_ = 37 °C) with an *R*_*f*_ of 98 % and an *R*_*r*_ of 98.5 %. In another study, a high-MW triblock polyurethane copolymer was produced by synthesizing a hydroxyl-terminating prepolymer through esterification of sebacic acid, azelaic acid, and 1,3-propanediol to form poly(propylene azelate-co-propylene sebacate) (PPAzSeb), connecting to an L-lactide segment [155]. The PPAzSeb blocks formed crystalline regions that acted as switching segments, while PLLA crystals are considered as physical netpoints due to their high *T*_*trans*_. The electrospun TMP can be programmed at 50 °C, cooled down to 30 °C, and then moved to −60 °C with 96 % *R*_*f*_. Upon exposure to 39 °C, the structure can revert to its original shape with a 99 % recovery ratio. Notably, this TMP showed suitable in vitro cytocompatibility when fibroblasts were seeded on it.

As discussed throughout this section, the temperature-responsiveness of TMPs can be tuned through four chemical parameters: composition, architecture, molecular weight, and crosslinking density of the TMP network (Fig. 11). The composition of the base polymer, whether copolymerization or blending, can tune the *T*_*trans*_ to the desired range, i.e., body temperature for the case of body-responsive SMPs. This could be achieved through tailoring the ratios between rigid and flexible monomers or polymers for copolymerization or blending, respectively. Molecular weight also influences *T*_*trans*_ by affecting free volume and chain entanglements; higher molecular weight results in lower free volume and longer chains, requiring more energy to break intermolecular forces, thus increasing *T*_*trans*_.

Architecture of side groups or the chains themselves may also impact the *T*_*trans*_ [50,51,124,145,147]. Bulkier side groups can hinder the crystallite formation, resulting in a lower T_trans_ in crystalline polymers or a limited entanglement in amorphous polymers. Additionally, side groups can introduce physical interactions that can affect the polymer properties. Branched or star structures decrease the crystallinity in the polymer, leading to lower *T*_*trans*_. In the amorphous polymer, changes in *T*_*trans*_ depend on the level of branching. Branching generally leads to higher junction density and reduced chain mobility, which in turn decreases its *T*_*trans*_. Crosslinking can also play a role in manipulating *T*_*trans*_, as a higher density of netpoints can increase stiffness, requiring more energy to move the molecular structures. High covalent crosslinking density generally increases the *T*_*trans*_ by restricting the segmental motion of chains. For physically crosslinked TMPs, increasing the strength or the density of netpoints can raise the transition temperature range, making it suitable for body-responsiveness. However, high crosslinking density can hinder crystallization in semicrystalline SMPs, leading to lower *T*_*trans*_. Recognizing how these factors interact encourages a comprehensive approach to TMP design.

Regarding the TMPs’ *R*_*f*_ and *R*_*r*_, the recovery for physically crosslinked TMPs is often high; however, repeated cycles at high strain or heat can gradually disrupt netpoints and disturb recovery and fixity of SMPs [61,156]. Chemically crosslinked TMPs usually do not face this issue and generally offer a higher *R*_*f*_ and *R*_*r*_. The density of these crosslinks, however, shows an inverse relation, where higher densities enhance shape fixity and deteriorate shape recovery as chain mobility decreases. This suggests that there exists an optimum point at which a TMP can be designed based on the target application. Overall, it should be emphasized that these parameters act synergistically to shape the behavior of TMPs; therefore, isolating the effect of a single parameter without considering the others is not practical when designing TMPs.

## 3 Water-responsive SMPs

Water is ubiquitous in the body, accounting for roughly 60 % of human weight [157,158]. It is present as either intracellular or extracellular fluid, making it a nontoxic and safe stimulus. Water, therefore, is considered an excellent trigger for designing body-responsive SMPs [[159], [160], [161], [162]]. Although water-responsive SMPs (WMPs) generally suffer from a limited lifespan due to poor mechanical properties and rapid degradation, they offer various capabilities, including cell encapsulation, tissue regeneration, and targeted drug delivery. Unlike TMPs, most WMPs cannot fully recover their initial dry shape; instead, they can revert to an intermediate shape [58,66]. The intermediate shapes typically have lower mechanical properties compared to those of the temporarily deformed shapes [158]. Depending on the chemical makeup of the polymer network, swelling and deswelling behaviors dictate the transition rate between each state. Water exchange results in the contraction or expansion of the materials, wherein the water gradient induces displacement. This behavior should not be confused with the time-dependent behavior of structures designed based on crosslinking mismatch that induces different swelling ratios [163]. As in WMPs, the response originates from the chemical makeup of the polymer used. The development of WMPs has been underexplored, highlighting their remarkable potential for future advancements in biomedical applications [164,165].

Hydrogen bonding is the dominant mechanism controlling the shape memory performance of WMPs [166,167]. The abundance of water stimulates competitive hydrogen bonding (H-bond) between water-susceptible motifs. The disruption of H-bonds through moisture absorption was accidently observed in 2004, when a PU-fixed film was left unattended [66,168,169]. The weakened H-bonds between N–H and C=O due to the penetration of water molecules are attributed to this shape change. Subsequent studies revealed that the presence of water increases the polymer network's entropy and decreases *T*_*g*_ by enhancing the chain mobility_,_ leading to a controlled and gradual recovery. The solvation of crystalline regions in water can also trigger water-induced recovery [58,170]. Water molecules can penetrate the hydrophilic crystalline structure, disrupting it and inducing shape recovery. The diffusion rate, which is tunable based on the application's needs, controls the recovery speed. Another emerging strategy is the incorporation of hydrophilic and hydrophobic polymer networks [58,169]. Adding a hydrophilic material, such as PEG or chitosan (CS), yields a higher recovery rate, while a hydrophobic polymer, such as PCL, serves as the stationary segment. Research on WMPs is progressing toward approaches that increase the recovery ratio while preserving the mechanical properties. As water infiltration into the structure is a time-consuming process, recovering the intermediate shape is not typically a swift process in the WMPs. H-bonds can also alter bonding sites with ions, including, but not limited to, protonation or metal cation addition [166,171,172]. Due to electrical charges, the cations and protonated groups can form new hydrogen bonding sites, thereby endowing the polymer network with pH responsiveness. The pH is another trigger for designing body-responsive SMPs, as it remains constant throughout the body with slight deviations under specific physiological conditions. Fig. 12 illustrates the molecular changes during the shape recovery process for a WMP.

### 3.1 Synthetic polymers

A feasible strategy for designing WMPs involves the incorporation of a hydrophilic polymer that serves as the switching segment into a hydrophobic segment that holds the permanent shape [6,173,174]. Among synthetic polymers, PEG is widely used due to its crystalline and hydrophilic nature, which enables it to act as a water-responsive switching domain in WMPs [6,173,175,176]. Introducing water to the semicrystalline network can disrupt the H-bonds of hydrophilic crystalline domains, leading to the swelling and softening of the structure. For example, adding PEG to PLGA led to the formation of a WMP, in which the dissolution of crystallites by hydration released the stored elastic energy in the network and actuated the shape recovery [176]. Theoretically, incorporating any hydrophilic crystalline domain capable of forming hydrogen bonding can initiate crystal solvation and shape change.

**Fig. 13:**
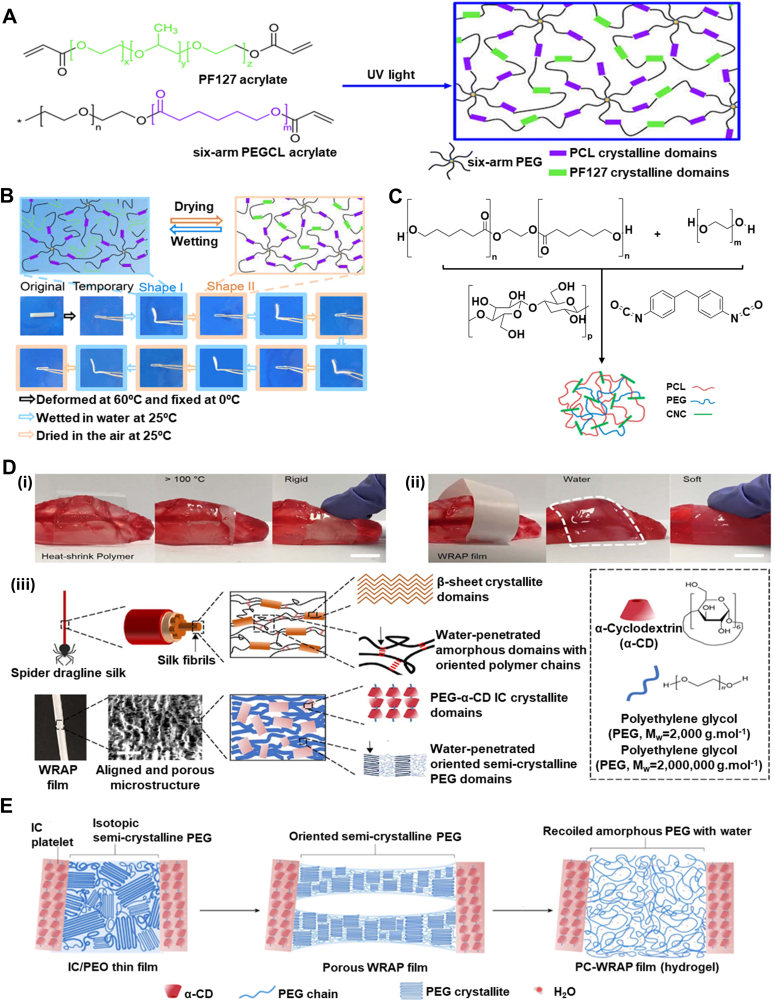
Synthesis and water-responsive shape memory behavior of PEG-based WMPs. A) Chemical structure of PEG-PCL copolymer and Pluronic® F127 as a precursor for creating WMP using PEG crystalline domains. Reproduced with permission [18]. Copyright 2022, Royal Society of Chemistry. B) Shape recovery mechanism and digital photographs of PEG-PCL copolymer with 60 % Pluronic® F127, showing the water-responsiveness due to PEG crystals. The network can be programmed at 60 °C by PCL crystallites and can undergo multiple shape changes by hydration/dehydration. Reproduced with permission [18]. Copyright 2022, Royal Society of Chemistry. C) Synthetic route of PEG-PCL copolymer connected via methylene diphenyl diisocyanate as a chain extender. CNC was grafted via the reaction between hydroxyl groups and existing isocyanates on the polymer chains. Reproduced with permission [19]. Copyright 2015, American Chemical Society. D) Photographs of (i) heat-shrinking, self-adaptive TMPs wrapping irregular agarose gel to mimic tissue. The structure contracts and wraps within seconds at 120 °C. (ii) WRAP film wrapping around by a drop of water resembling soft hydrogel on agarose gel after contraction (scale bar 1 μm). (iii) Schematic of supercontractile spider silk originating from its hierarchical structure, including water-sensitive amorphous segments crosslinked by β-sheets crystals. The oriented chains in the amorphous segments are fixed by hydrogen bonding, in which water can break the H-bonds, leading to molecular chain coiling. WRAP film is constructed based on a similar design using PEG-α-cyclodextrin inclusion complex (IC) as crystalline segments as permanent physical netpoints and water-sensitive high-MW PEG crystalline switching segments. The PEG crystalline domains can break in water, leading to the chain's recoil and contraction. Reproduced with permission [20]. Copyright 2023, Springer Nature. E) The semi-crystalline PEG segments are permanently crosslinked via IC platelets through hydrogen bonding. These segments are plastically deformed under cold drawing, forming oriented fibrillar and porous structures. Upon exposure to water, the crystallite recoils, resulting in an amorphous structure with soft properties. Reproduced with permission [20]. Copyright 2023, Springer Nature.

PCL-based TMPs with high *T*_*trans*_ can be manipulated in the scope of WMPs to create hydrophobic crystalline regions that act as physical netpoints [169,173]. As water cannot be absorbed into hydrophobic domains, the structure of WMP can maintain its permanent shape. At the same time, the hydrophilic segment (e.g., PEG) can change its shape in response to water absorption. As shown in Fig. 13A, a six-arm PEG-PCL copolymer was used in conjunction with Pluronic® F127 (an amphiphilic copolymer of PEG and polypropylene glycol) to design a WMP. The initial programming step was conducted at 60 °C through the deformation of PCL crystals. The structure became capable of reprogramming and recovering solely through hydration and dehydration cycles at 25 °C, as shown in Fig. 13B. The PEG-PCL could be further modified by grafting with cellulose nanocrystals (CNC) using methylene diphenyl diisocyanate, thereby enhancing the copolymer's mechanical properties (Fig. 13C) [177]. The shape change was dominantly governed by PEG crystal solvation, while the hydrogen bonding between CNC and water also introduced water responsiveness to the copolymer. This strategy, which involves preserving some crystals above body temperature, can enhance the mechanical properties of WMPs, as was also observed in TMPs [54]. Porosity facilitates water infiltration into the network due to a higher surface area, enhancing water responsiveness and crystal solvation in WMPs [6]. For example, the electrospun PCL-Siloxane-PEG copolymer showed better water uptake and shape memory properties than a non-porous structure made of the same material. Thus, creating macropores in the fabrication stage can, therefore, be an excellent approach to tuning the shape recovery time in WMPs.

Shape-adaptive TMPs are widely used for tissue-electronic interfaces, as they can wrap and cover tissues with irregular shapes and sizes [178]. However, the higher stiffness of TMPs compared to native tissues and the high temperature (above 100 °C) required for them to contract (Fig. 13D(i)) spurred researchers to design a water-responsive shape-adaptive polymer (WRAP) film using PEG and PEG-α-cyclodextrin inclusion. Fig. 13D(ii) shows a WRAP film wrapped around an irregularly shaped agarose gel. Upon activation by a drop of water, the WRAP film rapidly transformed into a soft hydrogel within seconds. This technique was inspired by spider silk, which exhibits excellent water-induced contraction, known as *supercontraction*. This term originated from the silk's hierarchical structure, in which oriented polymer chains within amorphous domains are stabilized by water-destructible hydrogen bonds and are crosslinked by stable β-sheet crystallites (Fig. 13D(iii)). In this design, the PEG-α-cyclodextrin domains function similarly to the β-sheet crystallites in the spider silk, while high-MW PEG (2,000,000 g mol^−1^) forms a compact, oriented semi-crystalline structure. The inclusion of complex platelets permanently crosslinks the isotropic PEG semicrystalline domains through H-bonds. Under uniaxial cold drawing, the PEG domains plastically deform to form aligned fibrillar bridges and porous structures. Simultaneously, the PEG crystallites and chains orient and are temporarily fixed by the newly formed PEG crystallites. Water causes the PEG chain to recoil and contract rapidly by destroying the PEG crystallites. After contraction, the PEG crosslinked by the inclusion complex becomes amorphous and water-rich, turning the post-contraction WRAP films into hydrogel films (Fig. 13E). Developing synthetic supercontractile materials using water poses a challenge, as excessive H-bonds restrict contraction [[178], [179], [180]]. At the same time, insufficient bonding leads to an unstable structure in ambient conditions. Achieving an optimal balance of intermolecular interactions is essential for the practical applications of WRAP films in biomedical settings. This will be particularly critical for tissue-electronic interfaces, where softness and adaptability are crucial.

**Fig. 14:**
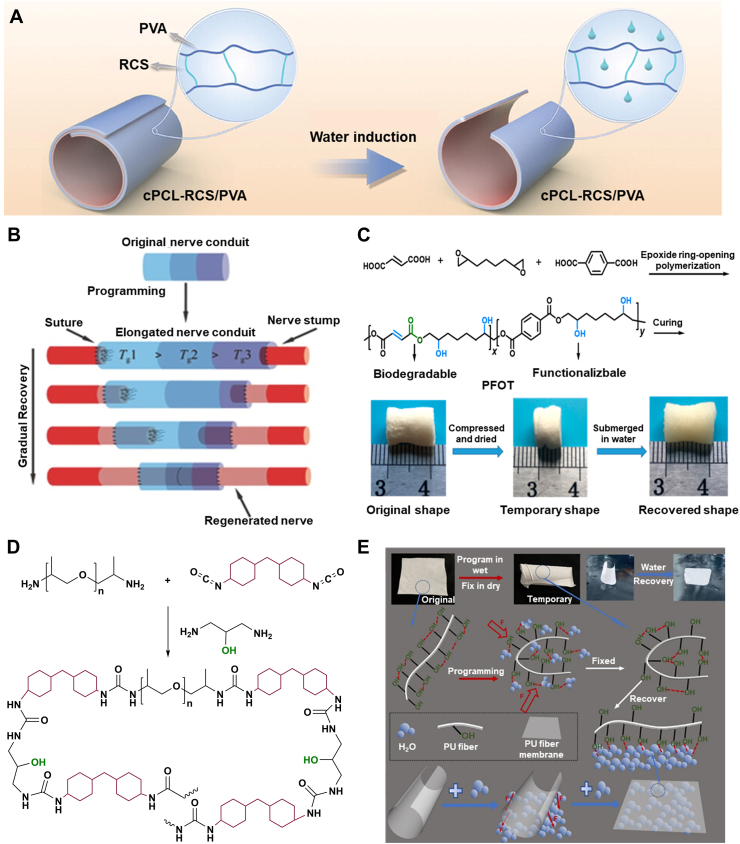
Synthesis and shape memory performance of WMPs made of synthetic polymers. A) Schematic of the water-induced shape recovery mechanism of the CS/PVA layer driven by the plasticization of entanglements between PVA and regenerated CS (RCS). Reproduced with permission [21]. Copyright 2024, Wiley-VCH. B) A smart nerve conduit made from multi-segment PLGA exhibits gradual recovery by water penetration as nerve regeneration continues. Reproduced with permission [22]. Copyright 2016, Wiley-VCH. C) Synthetic route for creating PFOT through ring-opening polymerization using terephthalic acid, octadiene diepoxide, and fumaric acid. The PFOT-based scaffold fully recovered after submerging the temporarily compressed and dried scaffold in water at 37 °C. Reproduced with permission [23]. Copyright 2019. American Chemical Society. D) Synthetic route for polyurea network formed using polyether amine, methylene-bis(4-cyclohexyl isocyanate), and 1,3-diamino-2-propanol, where aromatic groups act as physical crosslinks to maintain network integrity during programming. E) Original sheet of polyurea programmed in wet conditions, fixed by dehydration through rearrangements of hydrogen bonds. Upon exposure to water, the structure can form new bonds with hydroxyl groups, recovering to its original shape. Reproduced with permission [24]. Copyright 2024, Springer Nature.

Polyvinyl alcohol (PVA) stands out as another synthetic polymer for designing WMPs due to its biodegradability, non-toxicity, and inherent hydrophilicity [181,182]. PVA can mimic natural polymers with excellent characteristics, and it is compliant with human tissues. The crystalline domains in PVA can act as physical netpoints during programming and recovery, while amorphous regions deform upon water infiltration. The SMB is controllable by including a hydrophilic or a hydrophobic domain [[183], [184], [185], [186]]. This controllability is evidenced by the addition of silk fibroin and graphene oxide, both of which increase the available hydrogen bonding sites [184,186]. The large aspect ratio of graphene oxide and the β-sheets crystals in silk significantly enhanced the mechanical properties and SMB. Selective shape programming is also achievable by adding silica nanoparticles as a barrier to water penetration. Moreover, the addition of hydrophobic polymers can equilibrate the absorption and desorption of water, as demonstrated by the copolymerization of polyethylene dioxythiophene [185]. Although these strategies have improved the mechanical properties of PVA, they are still insufficient for biomedical applications, such as vascular grafts, as the tensile strength of human vessels ranges from 0.8 to 3.3 MPa, and the elongation at break is 49–105 % [182]. To circumvent this issue, researchers have designed a bilayer structure that includes PCL and PVA/CS as hydrophobic and hydrophilic layers, respectively. The robust entanglements between PVA and CS chains upon immersion in water weakened and began to plasticize (Fig. 14A), leading to a shape change. These entanglements act as physical netpoints in a dehydrated stage, as evidenced using crystallographic techniques, confirming that the shape change originates from alterations in the crystalline domains of PVA. In contrast, hydrophobic PCL crystallites remained intact in water. The tensile strength and elongation at break for different bilayer structures were reported to be in the ranges of 1.8–6.7 MPa and 348–765 %, respectively.

In general, water penetration into polymer networks plasticizes the amorphous regions, increasing the distance between chains and decreasing the *T*_*trans*_ [[187], [188], [189], [190]]. The polymer networks in this approach should have a distinct structural or chemical makeup that facilitates water infiltration. As a result, *T*_*trans*_ can gradually decrease based on the water content absorbed, varying based on the network's hydrophilicity. Plasticization can be manipulated for special clinical use, where the rapid shape change of TMPs can induce tissue damage [183]. Using this phenomenon, researchers used high-MW PLGAs to electrospin different scaffold segments with distinct transition temperatures [189]. These scaffold segments were physically crosslinked by chain entanglements and were utilized as a smart nerve conduit. As time passes, water penetrates the porous structure and lowers the *T*_*trans*_. Fig. 14B shows that the fabricated conduit can shrink over time as the natural nerve tissue regenerates. This behavior can also be achieved by incorporating hydrophilic groups into the polymer backbone [190,191]. Polybutanetetrol fumarate (PBF), a functional polyester synthesized from fumaric acid and butadiene diepoxide, has shown excellent water responsiveness due to hydrogen bonding and π-π interactions [190]. Although the *T*_*trans*_ of the structure is above 125 °C, water can plasticize the network and deform it into a temporary shape, which fixes upon drying. A similar strategy was used to synthesize a copolyester through the reaction between fumaric acid, terephthalic acid, and octadiene diepoxide (Fig. 14C) [191]. The synthesized copolyester, poly (fumaric acid-co-octadiene diepoxide-co-terephthalic acid) (PFOT), has hydrophilic and hydrophobic moieties responsible for shape recovery and fixity, respectively. The structure can be fixed and recovered upon exposure to dehydration/hydration cycles.

Inspired by the semi-crystalline and hierarchical hydrogen bonding of silk, researchers designed a polyurea based on the reaction of polyether amine, methylene-bis(4-cyclohexyl isocyanate), and 1,3-diamino-2-propanol, as shown in Fig. 14D [164,192]. The polyurea network is based on the stationary segments, the aromatic groups, and reversible hydrogen bonding from the urea and hydroxyl moieties. In the presence of water, new hydrogen bonds form between the water molecules and polyurea, as illustrated in Fig. 14E. After evaporation, the hydrogen bonds rearrange to form new bonds that contribute to the temporary shape. As the polyurea meets a wet surface, the diffusion of the water molecules allows the structure to unfold. The resulting WMP is a mechanically robust structure with a relatively rapid recovery time (25 s) and can potentially be used in various biomedical engineering applications, such as in wound dressings.

### 3.2 Natural polymers

Cellulose, the most abundant natural polymer, has been studied in different forms, such as CNC or nanofibrils (CNF) [165,[193], [194], [195]]. Cellulose derivatives can form interconnected percolating networks due to their abundant hydroxyl groups. The hydrogen bonding between hydroxyl moieties can be disrupted by water diffusion, leading to shape change. This phenomenon, therefore, can be employed to design WMPs. For instance, grafting CNC onto the hydrophobic isocyanate-modified PGS led to the formation of a hydrophilic network with water responsiveness [193]. Upon water immersion, the competitive hydrogen bonding between CNCs is replaced with water-CNC hydrogen bonding, softening the percolated network. The PGS-CNC wet structure can then be deformed and dried to fix its temporary shape by reorganizing the CNC network without the presence of water. Subsequent immersion in water leads to shape recovery due to the entropic recovery force originating from the structure. Water infiltration into the network may significantly deteriorate the mechanical properties of CNF, posing another restriction on the translational applications of cellulose-based WMPs [196]. For this reason, researchers have used CS as a crosslinking agent in the presence of a catalyst to enhance mechanical properties while preserving rapid water responsiveness.

**Fig. 15:**
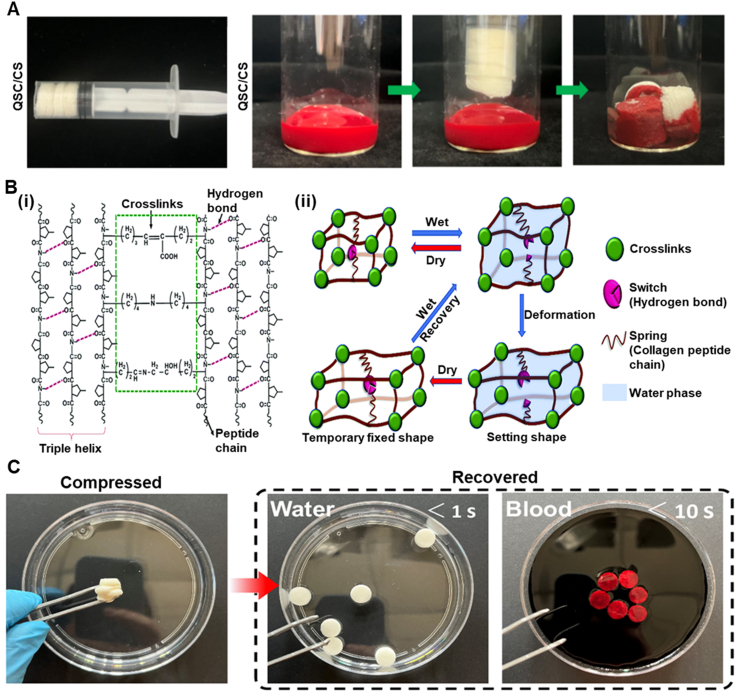
Natural polymer-based WMPs. A) Photograph of QCS/CS sponges compressed in a syringe in the dry state and their expansion upon exposure to blood. Reproduced with permission [25]. Copyright 2025, Elsevier. B) Schematic of (i) the molecular structure of collagen fibers showing chemical netpoints created from the reaction between lysine, hydroxylysine, and hydrogen bonding between peptide chains arranged in a triple helix configuration, and (ii) the proposed molecular mechanism for collagen water-responsiveness that employs hydrogen bonding in switching segments that contribute to shape changes during hydration/dehydration cycles. Chemical netpoints are stable under wet conditions and help the structure maintain its integrity. Reproduced with permission [26]. Copyright 2018, Royal Society of Chemistry. C) Photographs of a silk magnesium oxide WMP, showing a rapid shape recovery (within 10 s) triggered by water and blood. Reproduced under terms of the CC-BY license [27]. Copyright 2024, The Authors, published by Springer Nature.

Considerable attention has recently been given to CS as a natural polymer due to its biodegradability, biocompatibility, and wound-healing properties [[197], [198], [199]]. The extensive hydroxyl and amine groups on CS can be used for hydrogen bonding sites suitable for water-induced shape change. This polymer showed great miscibility with glycerol and quaternized CS (QCS) when used for designing WMPs. Hydrogen bonding between CS chains and chitosan-water acts as physical crosslinks and switching segments, respectively. The alteration in hydrogen bonding sites led to shape change, and higher glycerol content introduced better flexibility and shape memory performance, justified by more H-bond motifs. A similar trend has been shown with CS/QSC sponges used for wound hemostasis and wound healing [199]. In dried form, the interaction between these polymers preserves the shape. Upon blood uptake, the structure can retrieve its shape, as shown in Fig. 15A. Due to the semi-crystalline structure of CS, the shape memory behavior of CS/QSC sponges can also be attributed to the penetration of water into the amorphous domain as the switching segment. In contrast, crystalline segments serve as anchorage points to maintain structural integrity.

Collagen is a structural protein and a well-known natural polymer mostly found in biological tissues, and it forms almost 80 % of the dry weight of human skin [[200], [201], [202]]. The abundance of collagen in the skin provides it with exceptional mechanical strength, supports its complex hierarchical structure, and plays a vital role as a protective barrier against pathogens. Collagen is widely utilized in biomedical engineering to address various clinical challenges, including the repair of skin, tendon, and cartilage [[200], [201], [202], [203]]. It has been demonstrated that the numerous hydrogen bonds and crosslinks between peptides act as switching segments and stationary segments, respectively, as shown in Fig. 15B(i) and 15B(ii) [201]. The netpoints, originating from the reaction between lysine and hydroxylysine, stabilize the structure under wet conditions, while the interpeptide hydrogen bonds can be reversibly cleaved in water through unzipping the helix structures. The overall entropy provided by both segments contributes to the shape recovery of collagen-based WMPs. Temperatures above 50 °C could also disrupt interpeptide hydrogen bonds, but such temperatures may irreversibly denature the collagen. As a water-responsive protein, collagen's applications extend beyond pure networks, allowing it to be incorporated into other polymers as an additive to form composite biomaterials [201,204]. For instance, the collagen-PU composite network exhibits tuned water responsiveness, as evidenced by increased water uptake and reduced R_f_ compared to pure PU. It has also been shown that collagen fibers can be treated with tea polyphenols to control their hydration, flexibility, and shape memory properties [203]. Collagen fibers can interact with benzene rings in the tea-polyphenol, forming a gyro-mimicking linkage without damaging the collagen structures, and that can resist mechanical fracture under external forces. This unique behavior shows great potential for collagen in skin rejuvenation strategies and tissue engineering.

Silk fibroin, which is derived from the cocoons of insects or spiders, is another natural polymer used for scaffolds and implants due to its biocompatibility and cell support [[205], [206], [207], [208], [209]]. The α-helices and β-sheets structures in the silk, resulting from the repetitive polypeptide chains folding, are hydrophilic and hydrophobic domains, respectively. The β-sheets impede water absorption, while the α-helices enhance water absorption, providing the silk with toughness and elasticity [[205], [206], [207]]. Upon exposure to water, water molecules diffuse into the fibroin chains by breaking existing inter- and intramolecular hydrogen bonds and forming new bonds with polar groups. While the fibroin chains incorporate water molecules into the structure through conformation change, the hydrophobic β-sheets remain intact, creating a stress gradient that drives the shape change. Although silk-based WMPs have shown shape memory behavior, their mechanical properties (e.g., a compressive modulus of 58.8 kPa [206]) make them unsuitable for bone tissue engineering. For this reason, researchers have used magnesium oxide in combination with ethylene glycol diglycidyl ether to design porous WMPs capable of withstanding cyclic compression [206]. The negatively charged silk can form new crosslinks with Mg^2+^ positive charges, improving the compressive modulus. The addition of 10 wt% and 30 wt% magnesium oxide resulted in a two- and four-fold increase in the compressive modulus, respectively. As a result, the enhanced elastic modulus of 250–500 kPa fulfills the mechanical properties required for non-load-bearing bones. The fabricated structures can fully recover within 10 s after immersion in water or blood, as shown in Fig. 15C. In addition to the mechanism mentioned above, the internal stress made by the deformation of the amorphous structure in the silk may also contribute to the full recovery of the body-responsive WMP.

### 3.3 Charge-induced polymer networks

**Fig. 16:**
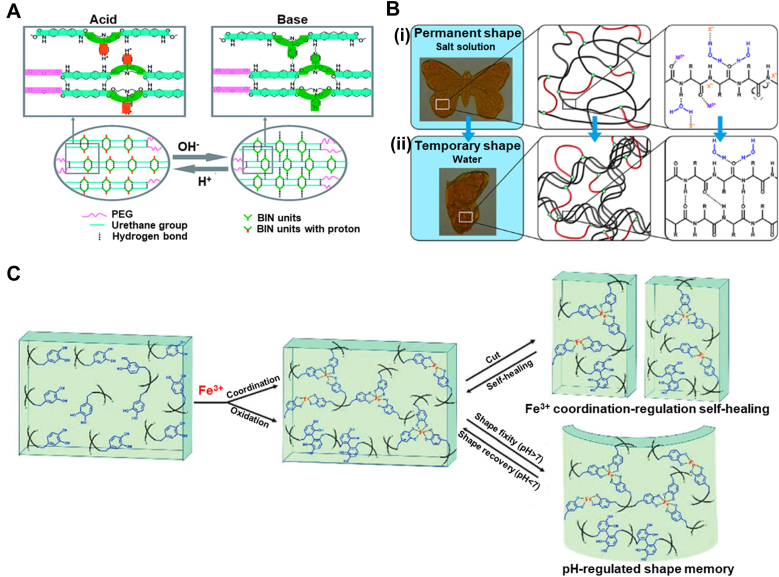
Shape memory behavior of charge-reversible polymer networks triggered by environmental changes. A) The protonation and deprotonation of the pyridine rings in the urethane linkages (formed by the reaction between BIN and PEG via diphenylmethane diisocyanate) disturb the hydrogen bonding under acidic and basic conditions, respectively, enabling the shape memory behavior. Reproduced with permission [28]. Copyright 2014, Royal Society for Chemistry. B) Removal of the salt solution induces a shape change (black lines represent gelatin, while the red lines are the crosslinks). The disruption in hydrogen bonding disrupted the chains' conformation. Reproduced with permission [29]. Copyright 2020, American Chemical Society. C) Illustration of self-healing and shape memory performance of Fe^3+^-catechol coordination. Reproduced with permission [30]. Copyright 2019, Royal Society of Chemistry.

Another category of WMPs comprises networks created from polymers with moieties sensitive to charges (i.e., cationic and anionic polymers) [171,172,210,211]. These networks can manipulate the hydrogen bonding between the polymer chains. Due to this characteristic, charge-induced polymers can exhibit shape transformations driven by pH changes within the body. Specifically, these polymers have great potential at pathological sites, where the gradient of pH is sharper compared to the overall constant physiological pH. Protonation of the amine groups in the polymer structure is one strategy used for shape change through pH [171,[212], [213], [214]]. For example, the urethane network from the reaction of PEG and N, N-bis(2-hydroxyethyl)isonicotinamide (BIN) via diphenylmethane diisocyanate provides the network with pH responsiveness [171]. The presence of the N atom in the pyridine group enables the structure to combine with a proton (H+), forming NH^+^ under acidic conditions. This led to a disruption in the hydrogen bonding between the amine in the urethane linkage and pyridine, which increased the penetration of water molecules and ultimately resulted in a shape change. Upon immersion in an alkaline solution, NH^+^ deprotonation diffused out the water molecules and maintained the structure's shape, as shown in Fig. 16A. This phenomenon was also observed in the existing amine groups in chitosan, which deprotonate in an alkaline solution, forming microcrystalline physical netpoints that function as fixation points for the structure [213,215]. The network can be fixed within 60 s and can recover its shape by immersion in an acidic solution. The pH responsiveness has also been observed within structures having carboxylic acid groups [[216], [217], [218]]. H-bonds can form between the carboxylic acid groups in an acidic environment; however, these bonds break upon immersion in an alkaline solution, leading to a shape change. This strategy can be implemented in TMPs to obtain dual responsiveness for minimally invasive stenting for esophageal [219], colorectal [220], and pancreatic [221,222] cancer due to the acidic shift in pH, allowing for the controlled delivery of chemotherapy drugs [223,224].

The relatively limited mechanical properties of WMPs with charge-induced polymer networks have spurred researchers to explore metal coordination interactions as crosslinking agents [[225], [226], [227]]. These physical netpoints strengthen the network and improve the shape memory performance of the network [226]. For example, adding Fe^3+^ to hyaluronic acid and PVA enhanced the structure's toughness through the introduction of metal ions and carboxylic acid groups. The metal–ligand interaction between the carboxylic acid groups and ions was found to improve the mechanical properties and shape memory performance, as well as reduce the recovery time from more than 60 s–27 s. Due to the dynamic structure of these interactions, the network also showed pH responsiveness to different metal cations [228,229]. For example, incorporating Mg^2+^ into a gelatin-based network has been reported to induce a reversible conformational change in the gelatin structure [230]. As depicted in Fig. 16B(i–ii), the chemically crosslinked gelatin construct possessed a permanent shape in an Mg^2+^ salt solution. Upon changing the media to water, the gelatin formed a helix structure due to the rearrangement of hydrogen bonds between the chains. In a similar strategy, a network was designed through the reaction between vinyl imidazole and acrylonitrile using polyethylene glycol diacrylate (PEGDA) as a crosslinking agent [231]. The imidazole group in the structure showed a complex formation in low concentrations of Zn^2+^_,_ which can be dynamically reversed by soaking the structure in ethylenediaminetetraacetic acid (EDTA). The cell-laden network can be fixed in the zinc solution and can retain its shape in the cell culture media after 2 h. This material system can be used for cell encapsulation and in the biomechanical analysis of cells. In Addition, it is suitable for antibacterial applications due to the presence of zinc and the low recovery rate, which allows Zn^2+^ to be removed from the network [231,232]. In another study, a polydopamine-modified PEG was used to form an Fe^3+^ complex with a catechol group that is susceptible to pH changes [233]. The metal coordination induced a shape change in an acidic environment, and the structure was fixed by reforming coordination in alkaline solutions. Due to the metal coordination bond breakage, the network regained its original shape when immersed in acidic environments. This Fe^3+^-catechol complex can improve the performance of hemostatic dressing due to its resistance to fibrinolysis as well as its antibacterial properties [234], while the dynamic interaction also imparts the network with self-healing properties (Fig. 16C) [233].

## 4 Dual-responsive SMPs

Harnessing the physiological conditions of the human body to trigger SMPs creates significant opportunities for advancing biomedical applications. Current research on temperature-responsive and water-responsive SMPs primarily focuses on tailoring the polymer structures to respond to a single stimulus. However, designing SMPs based upon a single stimulus that meets all essential criteria, such as mechanical integrity, body responsiveness, and precise recovery, is challenging, especially when attempting to replicate the intricate architecture of native tissues. This complexity can hinder the clinical translation of body-responsive SMPs. A strategy to overcome these limitations involves integrating responsiveness to both internal stimuli (body temperature and water) simultaneously. This dual-trigger approach can facilitate the development of SMPs that fulfill mechanical and functional requirements without relying on external stimuli that may pose risks to the surrounding tissues [29,235].

**Fig. 17:**
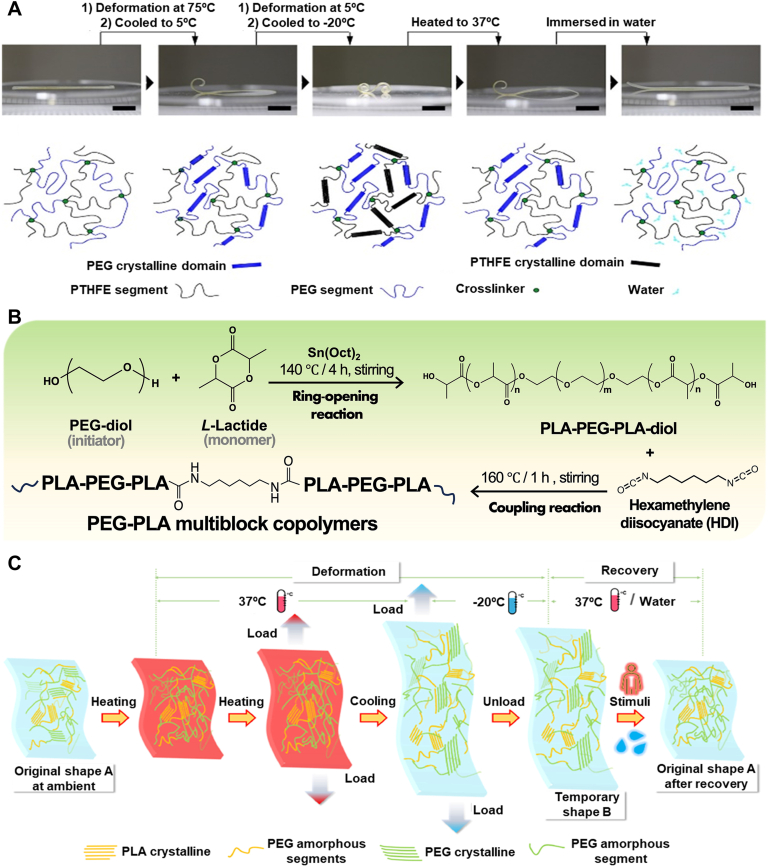
Synthesis and mechanism of a dual-responsive SMP based on PEG. A) Photographs and schematics of shape memory behavior in the PTHF-PEG network, showing two-step programming and recovery due to the crystalline segments of PTHF and PEG (scale bar: 1 cm). Reproduced with permission [31]. Copyright 2021, American Chemical Society. B) Synthetic route of PEG-PLA multiblock copolymer through ring-opening polymerization in the presence of Sn(Oct)_2_ as the catalyst. Reproduced with permission [32]. Copyright 2024, Elsevier. C) Schematic illustration of the underlying mechanism of a PLA-PEG copolymer, illustrating the presence of PLA crystalline domains as well as shape recovery due to the temperature- and water-responsiveness of PEG upon exposure to these stimuli. Reproduced with permission [32]. Copyright 2024, Elsevier.

Incorporating hydrophilic polymers with high *T*_*trans*_ is a straightforward strategy to design dual-responsive SMPs [[235], [236], [237]]. Researchers have designed SMPs by chemically crosslinking poly tetrahydrofuran (PTHF) and hydrophilic PEGDA to fabricate a body-responsive SMP [236]. Fig. 17A shows that the fabricated structure deformed in two stages based on the *T*_*trans*_ values for PEG and PTHF and was then fixed at lower temperatures. Upon heating to 37 °C, the PTHF crystalline segments recovered their initial shape, while the PEG crystals were unable to retrieve their shape until they were immersed in water. This dual-responsive approach enables a controlled drug release profile where the temperature triggers a burst release of the drug. At the same time, the subsequent water-triggered shape change facilitates a sustained release. In a similar study, a physical crosslinking strategy was used by copolymerizing L-lactide and PEG through ring-opening polymerization (Fig. 17B) [235]. The macro diols were coupled via HDI, forming PEG-PLA multiblock copolymers with semicrystalline PLA and PEG. Depending on the MW of each block, a *T*_*trans*_ in the range of 31.9–54.60 °C and a water uptake of 40–328 % were achieved. Specifically, a multiblock copolymer with PEG (4000 g mol^−1^) and PLA (1500 g mol^−1^) showed excellent body responsiveness with an *R*_*r*_ of 99.5 %. As illustrated in Fig. 17C, the network can be programmed at 37 °C using PEG crystallites as switching segments to allow the crystals to reform, while PLA crystals remain intact. Upon exposure to water and heat, the network can retrieve its original shape.

**Fig. 18:**
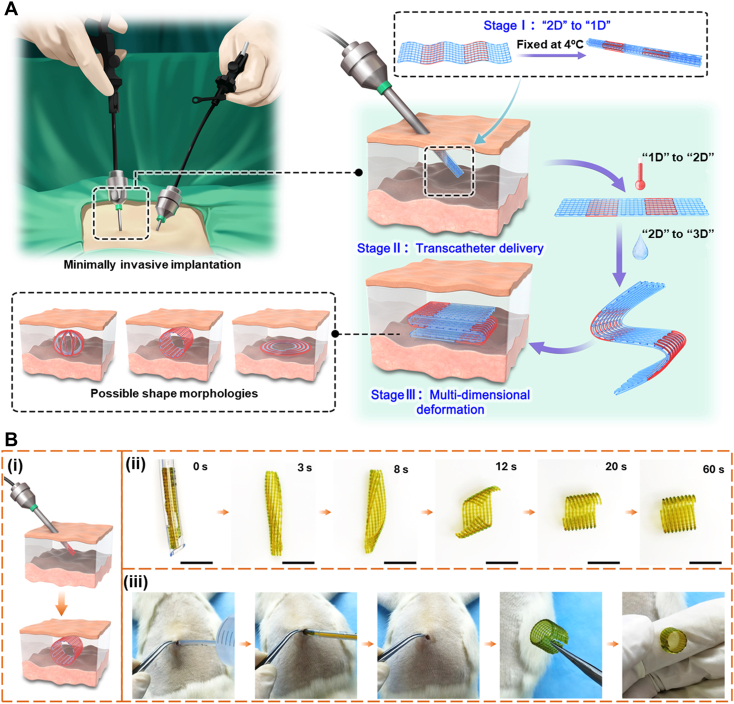
Shape memory performance of a dual-responsive dynamic thermoset PU. A) Dual-responsive shape memory PUs can respond to temperature and water sequentially, transforming a 1D structure to 3D for the implantation of large scaffolds through minimally invasive procedures. Reproduced under terms of the CC-BY license [33]. Copyright 2024, The Authors, published by Springer Nature. B) (i) Schematic of a 3D-printed structure delivered through a catheter to the implantation site, (ii) Photographs of 1D-to-3D shape recovery within 60 s upon exposure to body stimuli. (scale bar: 10 mm), (iii) Shape recovery of a subcutaneously implanted 1D structure in a rat to a 3D structure upon injection of 37 °C water. Reproduced under terms of the CC-BY license [33]. Copyright 2024, The Authors, published by Springer Nature.

Implanting large 3D scaffolds through minimally invasive procedures can be challenging, as the deformed implant may not fit the access route [152,237]. To alleviate this issue, a promising strategy involves delivering a “so-called” one-dimensional (1D) structure that changes into a 3D shape in response to physiological stimuli at the implantation site [152]. This approach relies on dual-responsive SMPs capable of multi-dimensional shape change. Fig. 18A shows that a structure programmed from two-dimensional to 1D at 4 °C was able to recover its two-dimensional shape at body temperature. Water, as the secondary stimulus, then enabled the transformation into a 3D structure. This strategy used a dynamic thermoset PU synthesized from PCL-triol, with PEG as switching segments. Water uptake and structural properties can be tailored by adjusting the ratio of PCL to PEG, which inevitably influences body-responsiveness due to alterations in hydrophobicity. Higher PEG content enhances polymer chains’ mobility and facilitates recovery at body temperature but reduces the crosslink density due to the lower functionality of PEG. Dynamic netpoints, formed via Diels-Alder chemistry, allow the extrusion of fabricated microfilaments for extrusion-based 3D printing. Printed structures can be fixed at 4 °C and recovered at 37 °C, and they can swell depending on the PEG content. Interestingly, PEG with a MW of above 2000 g mol^−1^ demonstrates a swelling-stiffening phenomenon. This feature improved mechanical properties by inducing phase separation between the hydrophilic and hydrophobic segments, preventing water-induced weakening within the body. Fig. 18B illustrates that a 1D scaffold can be transformed into a 3D structure via catheter-based implantation; the digital images revealed a step-wise deformation from 1D to 3D for printed structures exposed to body stimuli. Feasibility was also demonstrated in rats, where the scaffold was subcutaneously delivered. Upon injection of 37 °C water, the scaffold completed its deformation within 3 min, showcasing its potential for precise delivery of stents and scaffolds with reduced trauma. These strategies can be further extended by integrating WMPs and TMPs into advanced multi-material printing platforms to develop novel body-responsive constructs for clinical applications [238]. Notably, such approaches enable the fabrication of therapeutic grippers designed for gastrointestinal luminal delivery that can latch onto mucosa to facilitate localized release [239]. Similarly, such dual-responsive SMPs can be leveraged to create implantable soft actuators (such as twining electrodes) for precise and controllable shape activation, thereby enhancing their functionality [240,241].

## 5 Fabrication technologies

Various fabrication techniques have been explored for creating body-responsive SMPs, including advanced methods such as 4D printing, molding, foaming, electrospinning, and emulsion-based techniques. Each technique offers unique advantages and limitations, affecting the material properties and potential biomedical applications of the SMP construct.

### 5.1 4D printing techniques

**Fig. 19:**
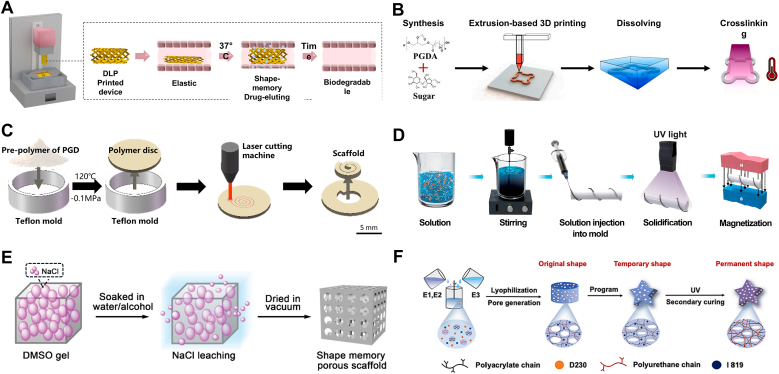
Fabrication techniques for creating SMP constructs. A) Schematic of a body-responsive, elastic, biodegradable stent fabricated using digital light processing 3D printing. Reproduced under the terms of the CC-BY license [1]. Copyright 2023, The Authors, published by Elsevier. B) Fabrication process for porous PGDA structures, where PGDA is synthesized and mixed with sugar to create printable inks. Sugar is dissolved after extrusion 3D printing to create a porous microstructure. Scaffolds are crosslinked using UV light and cured in a vacuum oven. Reproduced with permission [34]. Copyright 2023, Elsevier. C) Schematic of the hybrid fabrication process based on molding and laser cutting for PGD scaffolds with tendril structures. Reproduced under terms of the CC-BY license [8]. Copyright 2023. The Authors, published by Springer Nature. D) Fabrication process for shape memory magnetic microrobots. Reproduced with permission [35]. Copyright 2024, The American Association for the Advancement of Science. E) Creating a PGA shape memory porous scaffold with NaCl particles as the porogen. Reproduced with permission [11]. Copyright 2018, Royal Society of Chemistry. F) Schematic of a two-stage thermo-photo curing strategy involving lyophilization to generate pores, mechanical programming, and UV secondary curing to fix the permanent shape. Reproduced with permission [6]. Copyright 2024, Elsevier.

Among advanced methods, 4D printing, particularly vat photopolymerization (VP) methods, including digital light processing (DLP), has emerged as the leading approach for fabricating shape memory structures. DLP's high resolution and fabrication speed make it suitable for fabricating intricate microarchitectures for tissue engineering applications [238]. For example, an AESO-based ink was developed to fabricate 4D smart scaffolds [89]. Using a bottom-up DLP 3D printing approach, biocompatible scaffolds with subtle surface micropatterns and shape-changing properties were 3D-printed, supporting human mesenchymal stem cells' cardiomyogenic differentiation. Similarly, DLP was employed to 3D print biodegradable elastomers with shape recovery at 37 °C (Fig. 19A) [84]. However, the SMP resins exhibited high viscosity (>1000 cP) due to the long-chain nature of these polymers. The high viscosity can hinder smooth layer recoating and lower print fidelity, restricting the material's compatibility with commercial VP printers [242]. More recently, bioresorbable shape memory elastomers were developed by copolymerizing PGDA and acrylic acid [119]. By precisely formulating the ink composition to achieve low-viscosity resins (<100 cP), the DLP printing approach enabled the high-resolution fabrication of scaffolds with microfeatures and tunable transition temperatures ranging from 39.2 °C. Further research is needed to optimize the nano- and microporosity of these scaffolds for enhanced nutrient and oxygen transfer.

**Fig. 20:**
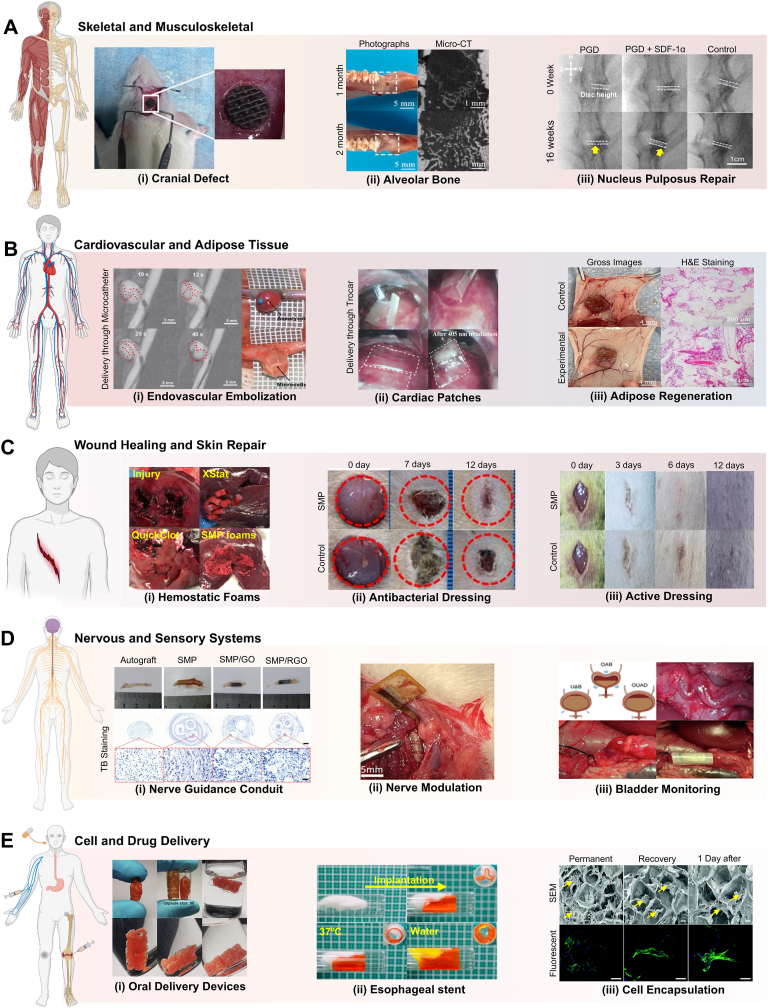
Examples of early-stage conceptual biomedical applications for body-responsive SMPs (TMPs, WMPs, and dual-responsive) across organ systems and therapeutic areas. A) Skeletal and musculoskeletal applications: (i) Scaffold implantation in the cranium of a rat. Reproduced with permission [2]. Copyright 2023, Wiley-VCH. (ii) Photographs and micro-CT scans of alveolar bone repair using SMP scaffold within two months. Reproduced under terms of the CC-BY license [12]. Copyright 2023, The Authors, published by KeAi Chinese Roots Global Impact. (iii) X-ray images of the nucleus cavity of the rabbit at 0- and 16-week post-implantation for PGD, PGD + SDF-1α, and the no-scaffold group as the control. The yellow arrow represents the platinum ring for visualization in implant surgery. The anatomical directions are H (head, F (foot), V (ventra), and D (dorsa). Reproduced under terms of the CC-BY license [8]. Copyright 2023, The Authors, published by Springer Nature. B) Cardiovascular and adipose tissue applications: (i) Endovascular embolization through a microcatheter using rabbit models, where microcoils fill the aneurysm within 40 s at the animal's blood temperature. Gross images showed successful clotting after implantation. Reproduced with permission [5]. Copyright 2023, Wiley-VCH. (ii) Pieces of cardiac patch delivered using a catheter into a canine model. The patch was then reconnected using 405-nm irradiation. Reproduced with permission [17]. Copyright 2023, Royal Society of Chemistry. (iii) Gross and H&E staining images collected at 12 weeks post-implantation of modified low-fouling, experimental, and unmodified scaffolds implanted on mice. Reproduced with permission [36]. Copyright 2023, Elsevier. C) Wound healing and skin repair: (i) Images of grade V liver injury and the treatment via XStat, QuickClot, and SMP foams. Reproduced with permission [4]. Copyright 2022, Elsevier. (ii) Images of infected wounds treated with SMP foams at 0, 7, and 12 days. Reproduced with permission [25]. Copyright 2025, Elsevier. (iii) Wound healing progress in rat models in the presence of active dressing after 12 days. The wound in the control group was wrapped with gauze. Reproduced with permission [24]. Copyright 2024, Springer Nature. D) Nervous and sensory systems: (i) Photographs of harvested nerve guidance conduits implanted in a rat sciatic nerve defect model and toluidine blue (TB) staining of regenerated nerves at 12 weeks after surgery. Reproduced with permission [37]. Copyright 2024, Wiley-VCH. (ii) Image of a wrapped electrode functioning as a nerve modulator around the vagus nerve in a rat model. Reproduced with permission [38]. Copyright 2024, Wiley-VCH. (iii) Bladder disorders such as overactive bladder (OAB), underactive bladder (UAB), and other urinary-affecting disorders (OUAD) need volume monitoring. The diagram shows the implantation area of the monitoring device in the urethral region of a pig model and the device arrangement. Reproduced with permission [39]. Copyright 2024, Elsevier. E) Drug and cell delivery systems: (i) Programmed oral drug delivery device in a gelatin capsule, recovering within 17 min in 37 °C water. Reproduced under terms of the CC-BY license [40]. Copyright 2025, The Authors, published by Elsevier. (ii) Images of an esophageal drug-eluting stent in a glass tube releasing the drug to cotton as a tumor model. Reproduced with permission [32]. Copyright 2024, Elsevier. (iii) scanning electron microscopy (SEM) and fluorescent images of porous SMP cross-sections in their permanent shape, the recovery stage, and one day after recovery (scale bar: 100 μm). Reproduced with permission [11]. Copyright 2018, Royal Society of Chemistry.

**Fig. 21:**
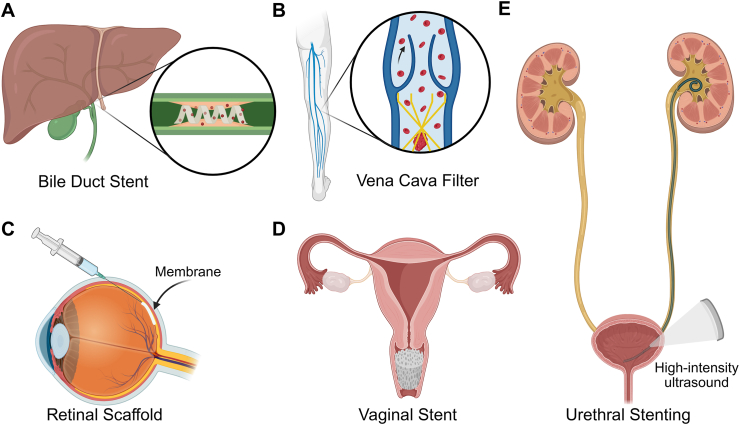
Illustration of other biomedical applications of body-responsive SMPs. A) A drug-eluting stent for treating biliary stricture. B) A deployable inferior vena cava filter intended to capture thrombi within the body. C) Subretinal injection of an ultrathin shape memory membrane serving as a scaffold for tissue engineering. D) A self-fitting vaginal stent that conforms after implantation. E) Retrieval of urethral stenting using high-intensity ultrasound to locally change the shape for less damage during the procedure.

Recent studies have addressed the limitations of 3D printing by incorporating porogen into the material and leaching it out after the printing process [[243], [244], [245]]. Porous scaffolds with shape memory properties were fabricated using DLP-printed acrylated urethane-based PCL [55]. Adding porogen to the resin enabled controlled porosity in the scaffolds, which recovered their shape when heated above the melting temperature of PCL. Although these resins offered low viscosity and suitable reaction kinetics, the need for porogen posed challenges in balancing resolution and porosity. Other 3D printing methods, such as extrusion-based 3D printing, offer broader material versatility, including high-viscosity and particle-laden inks. Extrusion-based printing was utilized to create hierarchically porous structures by incorporating sugar microparticles as sacrificial porogen into a PGDA ink (Fig. 19B) [109]. While this method allows for the processing of a broader range of biomaterials, its lower resolution compared to VP methods can limit its applicability in precision-dependent applications.

The selection of 3D printing techniques significantly influences the quality and clinical translation of SMP-based biomedical devices. VP methods are limited to photocurable materials and typically require precise tuning of polymer composition to achieve efficient photopolymerization. Rapid curing is particularly crucial to avoid over-curing, which can otherwise compromise the print resolution and accuracy. However, because many SMPs are based on long-chain polymers, the molar concentration of the functional groups available for photocrosslinking is often limited, presenting significant challenges in achieving efficient and fast curing. Using biocompatible photoinitiators and photoabsorbers is also crucial for cytocompatibility, supporting the safe use of printed scaffolds in clinical settings. Moreover, the 4D printing largely centers on TMPs, although their synthetic nature raises issues regarding their biological performance [[246], [247], [248], [249]]. Combining temperature-responsiveness with other triggers (e.g., water, pH, or light) can extend to 4D bioprinting. The inclusion of various stimuli enables multi-responsive behaviors, moving the field toward SMPs that can adapt complex functionalities. For instance, a TMP may activate the primary shape change, while a WMP response can be programmed for drug release. Likewise, as WMPs are inherently water-rich, they can be used to accommodate cells during the shape transformation to minimize cellular damage. It is noticeable from the WMP section of our Table 1 that this category of SMPs is unexplored in the field of 4D printing.

**Table 1:** Mechanical and shape memory properties of body-responsive SMPs by stimulus type, switching segment, and fabrication technique. Shape memory data are reported by study-specific test conditions; *R*_*r*_ was measured at 37 °C and/or in water unless noted. Mechanical properties reflect the original study conditions. Unless stated otherwise, the conditions reported for one mechanical property apply to all others. (NR: not reported; RT: room temperature; RH: relative humidity).

Polymer Network	Switching Mechanism		Fabrication	*R*_*f*_ [%]	*R*_*r*_ [%]	Elastic Modulus [MPa]	Tensile Strength [MPa]	Strain at Break [%]	Ref.
*Temperature-responsive Shape Memory Polymers (TMPs)*									
*CHEMICALLY CROSSLINKED*									
PLLA-PTMC (70:30)	Amorphous: 37 °C		4D printing	97	99	1.63 (37 °C)	NR	30	[250]
PLLA-PTMC (80:20)	Amorphous: 32 °C		4D printing	100	98.5	2 (RT)	1.4	125	[84]
AESO	Amorphous: 20 °C		4D printing	92–99	100	NR	NR	NR	[88,89]
Piezoelectric AESO	Amorphous: 37 °C		4D printing	NR	NR	NR	NR	21.4 (RT)	[90]
Acrylated olive oil/acrylic acid	Amorphous: 19.8–25.2 °C		4D printing	∼75–99	NR	0.8–5.1	0.15–1	20–60	[91]
PGSA-PHEMA (55:45)	Crystalline: 37.8 °C		4D printing	97.3	92.9	NR	10.6	∼37	[112]
Acrylated PCL	Crystalline: 37 °C		4D printing	∼95	∼94	NR	NR	45	[55]
Polyaminoester-stearyl acrylate-PVP	Crystalline: 36.6 °C		4D printing	100	98.8	1 (40 °C)	NR	700	[251]
Stearyl acrylate/lauryl acrylate (75:25)	Crystalline: 37.2 °C		4D printing	99.8	99.5	59.4	NR	102.7	[113]
PGDA-Polyacrylic acid	Crystalline: 37 °C		4D printing	90	100	40 (RT)	7 (RT)	170 (RT)	[119]
PGDA	Crystalline: 26.7–36.2 °C		4D printing	40–97.7	78–90.2	3–5 (37 °C)	NR	14–62	[68,118]
	Crystalline: 23.2 °C		4D printing + Thermal curing	100	98	15.4 (20 °C) 3.2 (37 °C)	5.1 1.1	210 35.5	[115]
Porous PGDA	Crystalline: 35.6 °C		4D printing	∼99	∼100	36.8–39.7 (RT)	NR	7	[109]
PCL-PTMC	Crystalline: 22–33 °C		4D printing	NR	NR	NR	NR	NR	[252]
PLA-PEG-Cinnamic acid	Crystalline: 31–37.5 °C		4D printing	97–98.5	90–91	NR	NR	3317–1209	[253]
Acrylate network	Amorphous: 37 °C		4D printing + Molding	85.7	95.7	150	8.76	NR	[100]
PLLA-PTMC (60:40)	Amorphous: 29 °C		Molding	NR	98	3.3 (RT)	15.1	431	[80]
PLLA-PTMC (70:30)	Amorphous: 30 °C		Molding	98.2	95.1	1.7 (40 °C)	4	680	[82]
PLLA-PTMC (50:50)	Amorphous: 22.4 °C		Molding	NR	83	2.1 (RT)	0.57	620	[81]
Acrylated PDLLA-PCL	Amorphous: 27.3–37.8 °C		Molding	87–99.5	77–99.4	NR	NR	NR	[254]
Magneto-acrylate network	Amorphous: 25–37 °C		Molding	92.5	82.8	31	0.68	NR	[101]
Tert-BA/BA	Amorphous: 36 °C		Molding	97.5	91.7	NR	NR	NR	[255]
Sulfobetaine methacrylate – aminoethyl methacrylate isopropyl carbamate (1:6)	Amorphous: 36 °C		Molding	∼97	∼95	21.9	2.42	200	[256]
Stearyl acrylate/lauryl methacrylate (3:7)	Crystalline: 21.1 °C		Molding	NR	NR	NR	NR	NR	[114]
PGD	Crystalline: 32.1 °C		Molding (96 h cure time)	NR	100	1.08	NR	123.2	[105]
	Crystalline: 37 °C		Molding (68 h cure time)	>90	100	70	NR	NR	[117]
	Crystalline: 35 °C		Molding (36 h cure time)	∼97	∼98	NR	NR	NR	[116]
PGD (Glycerol/DDA = 3:1)	Crystalline: 35 °C		Molding (72 h)	100	100	1.1	NR	∼80	[49]
PGS-Stearate	Crystalline: 29 °C		Molding	86	85	NR	NR	NR	[110]
PCL-Isosorbide-castor oil	Crystalline: 30.14–36.28 °C		Molding	94–97.5	92–96	NR	NR	596–1319	[257]
PCL-Glycidyl methacrylate	Crystalline: 35–40 °C		Molding	95.2	97.35 (42 °C)	24.7 (37 °C)	3.65	23.2	[123]
Three-arm methacrylate PCL	Crystalline: 36.2 °C		Molding	99	98 (40 °C)	134 (RT)	NR	NR	[258]
PGS-PPS-Kartogenin	Crystalline: 37 °C		Molding + Leaching	98	97	NR	NR	NR	[111]
PU	Amorphous: 25 °C		Foaming	∼70	100	NR	NR	NR	[48]
Vanillic acid incorporated PU	Amorphous: 25–30 °C		Foaming	NR	100	NR	NR	NR	[259]
PCL/PLLA-PTMC/Gelatin methacrylate	Amorphous: 37 °C		Electrospinning + Molding	NR	NR	5.3 (Wet)	12.7	21.7	[260]
PLLA-PTMC (80:20)	Amorphous: 36.7 °C		Electrospinning	99	97.6 (39 °C)	∼175	∼4	∼75	[261]
PLLA-PTMC (70:30)	Amorphous: 37.8 °C		Solution casting	NR	NR	4	20	∼500	[262]
PEG/PVP (90:10)	Amorphous: 37 °C		Solution casting	NR	83	NR	NR	NR	[98]
Star PCL	Crystalline: 29.1–46.1 °C		Solution casting	100	100	NR	NR	NR	[50]
Diacrylate PCL/Tetra-acrylate PCL	Crystalline: 33–37.8 °C		Solution casting	98–99.5	89.8–90.5	NR	NR	NR	[263]
PGA-g-PCL–PPDL	Crystalline: 37 °C		Solution casting + Particulate leaching	97	87	0.001	NR	70	[54]
PGA-g-PCL	Crystalline: 35 °C		Particulate leaching	97	68	NR	NR	NR	[121]
Acrylate Emulsion	Amorphous: 25–37 °C		Lyophilization	97	95	NR	0.17	85	[102]
PDLLA-grafted-polyacrylic acid/PEG	Amorphous: 39.3 °C		Photolithography + Coating	NR	∼100	∼1.1	5.36	∼769	[264,265]
PCL Dimethacrylate	Crystalline: 39.85		UV-assisted electrospinning	97.7	92.3	35 (RT)	4.8	NR	[266]
Star-PCL foam	Crystalline: 43 °C^a^		Emulsion templating	95	100	NR	NR	NR	[122]
*PHYSICALLY CROSSLINKED*									
PLA/PEG (80:20)	Amorphous: 38.2 °C		4D printing	NR	NR	NR	8.7	18.5	[128]
PLA/PEG	Amorphous: 34 °C		4D printing	97.6	99.1	NR	NR	NR	[267]
PLLA-PTMC/Polyglycolic acid-PTMC	Amorphous: 26.74–29.43 °C		4D printing	NR	NR	NR	∼15–28	∼220–400	[130]
PCL-urethane (PCL diol reacted with isocyanate)	Crystalline: 37 °C		4D printing + Molding	>97	>97	0.3	NR	110	[268]
PLA-POSS-Hydroxyapatite	Amorphous: 37 °C		Molding	99.8	98.7	3.3 (37 °C)	1.7	315	[269]
(PDLLA):HDI:Piperazine (1:1.1:0.1)	Amorphous: 35.85 °C		Molding	95.3	98.6	625 (RT)	17.5	725	[143]
PU (PDLLA-PEG400-PDLLA:HDI-ISO-HDI) (1:1.8:2.78)	Amorphous: 42.2 °C		Molding	99.6 (60 °C)	90.2	1203	29.6	219	[144]
PGCL:(PCL:POSS (70:30))	Amorphous: 14 °C		Molding	NR	90	10.95	NR	>1200	[145]
PU (PCL:isophorone diisocyanate:BDO)	Crystalline: 38–41 °C		Molding	93–95	65–75	7–230	25.3–54.8	950–1570	[270]
PCL-HDI-castor oil/hydroxyapatite	Crystalline: 40 °C		Molding	94	91 (37 °C)	NR	NR	NR	[271,272]
PCL:HDI	Crystalline: 31.4 °C		Molding	NR	81	2.11	NR	>1200	[145]
PLLA-PGCL (80:20)	Amorphous: 40.9 °C		Solution casting	95.6	95.7	NR	32.5	425	[131]
PDLLA-PUU	Amorphous: 38 °C		Solution casting	100	98 (53 °C)	NR	NR	NR	[142]
PCLSe	Crystalline: 36 °C		Solution casting	99.5	94.4 (45 °C)	188 (40 °C)	11.25 (45 °C)	7.04 (45 °C)	[152]
PCL-AT	Crystalline: 33–37 °C		Solution casting	61–78	100	31.2–44.3	NR	646–983	[273]
PCL-AT-IPDI-CCYS	Crystalline: 31 °C		Solution casting	98	90 (40 °C)	17 (RT)	10.5 (RT)	193 (RT)	[274]
PCL-PEG-AT	Crystalline: 27.9–34.7 °C		Solution casting	NR	72–98 (38 °C)	5.2–21.2 (RT, Dry) 2–10.3 (RT, Wet)	9.1–16 4.9–8.3	278–1152 185–908	[275]
PCL-PEG	Crystalline: 15 °C & 49.5 °C		Solution casting	NR	70 (37 °C)	61.94	1.55	38.75	[146]
PUU-PVLCL (10:90)	Crystalline: 35 °C		Solution casting	NR	87 (60 °C)	NR	NR	NR	[151]
PU (HDI:PCL)	Crystalline: 37 °C		Solution casting	94	91	NR	NR	NR	[271,272]
PLGA-PTMC	Amorphous: 41 °C		Salt-leaching	NR	94 (37 °C)	4.2	NR	NR	[135]
PLGA-PCL	Amorphous: 45.1 °C		Salt-leaching	NR	91 (37 °C)	3.1	NR	NR	[135]
PCL-hydroxyapatite-Allyl alcohol	Crystalline: 37 °C		Salt leaching	NR	91 (37 °C)	NR	NR	NR	[276]
PLLA-P(PAz60PSeb40)	Crystalline: 39 °C		Electrospinning	99 (0–50 °C)	96 (0–50 °C)	NR	NR	NR	[155]
PCL-co-PDMS (70:30)	Crystalline: 37.7 °C		Electrospinning	90.14 (0–60 °C)	95.69 (0–60 °C)	4.3	7.93	155.4	[147]
PSf-g-PEGMA (27:73)	Crystalline: 37.1 °C		Spin coating	NR	99	3.54	0.25	31.6	[153]
PLCL/PLGA	Amorphous: 37 °C		Spin coating + Vapor-induced phase separation	97.06	68.67	1.77 (Dry) 1.5 (60 % RH)	NR	∼3.5	[277]
PLCL (75:25)	Amorphous: 30 °C		Thermally-induced phase separation	98.3	96.5	NR	NR	NR	[133]
PUU (MDI:IU:PEG)	Amorphous: 37 °C		Film swelling	NR	≈100 % (60 °C)	NR	1.1	600	[51]
PU [PCLA (20 % CL):BDO:TDI] (1:7:4)	Amorphous: 40.2 °C		Hot Pressing	NR	94 (39 °C)	740	43	440	[278]
Polylactide PU (PLLA:BDO:HDI) (1:5:4)	Amorphous: 33 °C		NR	98	100	603 (20 °C)	45	236	[140]
*Water-responsive Shape Memory Polymers (WMPs)*									
PU/Cellulose nanowhiskers	H-bond + Hydrophilic hydrophobic interaction		Molding	74	55	1000 (Dry, RT) 144 (Wet, RT)	NR	NR	[195]
Chitosan/QCS	H-bond		Molding	NR	100 (PBS)	NR	NR	NR	[199]
PHEMA-Gelatin	Protonation through (NH_4_)_2_SO_4_		Molding	NR	≈100 (salt solution)	0–45 (Based on ammonium sulfate)	3–7.5	200–700	[210]
Polyacrylamide/sodium alginate	Coordination with Fe^3+^		Molding	∼83	∼100 (RT)	NR	0.12	850	[225]
PMATC^b^	Protonation of carboxylic acid groups and coordination with Na^+^		Molding	>95	≈90	NR	0.07–0.3 (RT)	250–500	[218]
Carbon nanotubes doped silk sericin	H-bond		Casting	≈98	∼100 (RT, PBS)	NR	NR	NR	[279]
Vinyl imidazole-acrylonitrile	Metal coordination with Zn^2+^		Casting	100 (ZnNO_4_ solution)	100 (EDTA solution)	0.6–37.8 (Wet)	0.15–4	23–45	[231]
PEG-PCL/Pluronic	Crystal solvation		Solvent casting	>95	60 (RT)	NR	NR	NR	[173]
PEG/PCL/CNC	Crystal solvation		Solution casting	100	86	NR	6.28 (RT)	161.28	[177]
PCL-Siloxane-PEG	H-bond + Crystal solvation		Solution casting	100	59.3 (RT)	46.2 (RT)	5.2	794	[6]
PEO/PEG-co-α Cyclodextrin	H-bond + Crystal solvation		Solvent casting	NR	60 % (contraction)	20 (RT)	NR	700	[178]
PEG-grafted PU	H-bond + Hydrophilic hydrophobic interaction		Solution casting	NR	63 (40 °C)	NR	21 (Wet, RT)	327	[174]
P(3HB-co-4HB)^c^	H-bond + Crystal solvation		Solution casting	NR	∼3 mm d	NR	5–20 (RT)	10–18	[280]
PGS/CNC	H-bond + Hydrophilic hydrophobic interaction		Solution molding	80	82	≈1.7 (RT)	12	400	[193]
Hydroxyethyl cellulose	H-bond + Plasticization		Solvent casting	NR	100 (RT)	NR	91.9 (RT) (40 % RH)	9.3 (RT) (40 % RH)	[165]
Chitosan/Cellulose nanofibrils	H-bond + Plasticization		Solvent casting	NR	100 (RT)	5	65 (24 h soaking)	21 (24 h soaking)	[196]
Chitosan/Glycerol	H-bond + Plasticization		Solvent casting	NR	89.4 (RT)	NR	NR	NR	[197]
Un-crosslinked Chitosan	H-bond + Crystal solvation		Solvent casting	>97 (RT)	>70 (RT)	0.03 (Dry, RT)	NR	NR	[198]
Genipin-crosslinked Chitosan	H-bond + Crystal solvation		Solvent casting	∼99 (RT)	∼99 (RT)	0.04 (Dry, RT)	NR	NR	[198]
Tea-polyphenol-treated collagen	H-bond + Plasticization		Solution casting	93.9	88.5	NR	11.5 (Wet)	25	[203]
Silk fibroin	H-bond + Hydrophobic-hydrophilic		Solution casting	NR	100 (RT)	NR	NR	NR	[205]
Silk Fibroin	H-bond + Hydrophobic-hydrophilic		Solution casting	NR	100	∼7.5 (Wet)	NR	NR	[208]
PVA/Silk fibroin	H-bond + Plasticization		Solvent casting	NR	100 (RT)	1.12 (Wet)	2.66 (wet)	457 (wet)	[186]
PVA/GO	H-bond disruption of GO-PVA		Solvent cast	NR	100	NR	≈100–150 (60 % RH)	NR	[184]
SiO2-coated PVA	H-Bond/Hydrophobic-hydrophilic		Solvent casting	NR	100 (RT)	NR	NR	NR	[183]
PVA/PEDOT	H-bond + Hydrophilic hydrophobic interaction		Solvent casting	NR	NR	NR	45 (30 % RH)	3.9	[185]
PVA/PCL/Chitosan	H-bond + Hydrophobic hydrophilic		Solvent casting	NR	NR	10.3–131.7 (RT)	1.8–3.6	745–777	[182]
PU (PEG–MDI–BIN(32 %))	pH/protonation of the pyridine ring		Solution casting	96 (pH = 10)	100 (pH = 1.3)	NR	NR	NR	[171]
Polyurea-methylene-bis(4-cyclohexyl isocyanate)	H-bond + Hydrophilic hydrophobic interaction		Solution casting	NR	90 (RT, 65 % RH)	NR	51 (RT)	372	[164]
PEG-modified dopamine	Metal coordination		Solution casing	100 (pH = 12)	98 (pH = 3)	0.0012–0.0241 (pH varies, RT)	0.006–0.045	1000–1600	[233]
Acrylamide grafted DNA	pH/protonated cytosine–guanine–cytosine triplex (pH = 5)		Solution casting	NR	NR	NR	NR	NR	[214]
CD and diethylenetriamine-modified alginate	pH/complex formation between CD and amines		Solution casting	94.8 (pH = 11.5)	95.7 (pH = 7.0)	5.07 (RT) (pH = 11.5)	6.65 (RT) (pH = 11.5)	NR	[212]
Polyacrylamide/chitosan	Protonation of amines and metal coordination with Na^+^		Solution casting	100	95	NR	0.1 (RT)	900–2500	[215]
Polyacrylamide/phenylboronic acid grafted alginate	Boric acid-alginate interaction and coordination with Ca^2+^		Solution casting	NR	100 (pH = 9–10)	NR	0.03–0.08 (RT)	20002500	[228]
PU (PEG-MDI-dimethyl propionic acid)	carboxylic group deprotonation		Solution casting	81 (pH = 9)	80 (pH = 2)	NR	0.4–1.49 (RT)	20–95	[216]
MPTC-NaSS-MAA^e^	carboxyl group protonation		Solution casting	NR	≈650°^f^	NR	225	600	[217]
Hyaluronic acid sodium/PVA	Coordination with Fe^3+^		Solution casting	93–95	96.6	NR	26.1–43.2	∼8–40	[226]
P(AM-AA-DMAEMA)^g^	Coordination with Fe^3+^ and Na^+^		Solution casting	98–100	93–98	NR	NR	NR	[227]
Gelatin methacrylate	Coordination with Mg^2+^		Solution casting	60–95 (Different salts and crosslinkers)	∼100	NR	NR	NR	[230]
Poly (isopropyl acrylamide) peptide	Protonation and peptide self-assembly		Solution casting	NR	642°	NR	NR	NR	[229]
TPU/CNF (76:24)	H-bond + Plasticization		Electrospinning + Solvent casting	70 (RT)	95 (RT)	NR	4.5–9 (cyclic) (RT)	NR	[194]
PCL/PEG	H-bond Crystal solvation		Electrospinning	91	83 (RT)	0.2 (RT)	2.4	742	[169]
PLGA	H-bond + Plasticization		Electrospinning	99	90	NR	NR	NR	[189]
Polydopamine-coated polyurea loaded with Ag nanoparticles	H-bond + Hydrophilic hydrophobic interaction		Electrospinning	NR	70–85 (RT)	NR	7–9.4 (Depends on water content)	570–790 (Depends on water content)	[192]
Silk Fibroin (Artificially made)	H-bond + Hydrophobic-hydrophilic		Fiber spinning (Data for each fiber)	∼73 (Average-Cyclic)	∼98 (75 % RH)	695.5 (RT)	40	NR	[207]
PBF	H-bond + Plasticization		Salt leaching	100 (RT)	100 (RT)	0.006–0.023 (Wet)	≈0.001–0.005 (Wet)	40	[190]
PFOT	H-bond + Hydrophilic hydrophobic interaction		Salt leaching	100	95	0.0198 (Wet, RT)	0.46	30	[191]
PLGA/PEG	Crystal solvation		Filament extrusion + Coating	100	88	1500 (RT)	35	NR	[176]
Collagen skin	H-bond + Plasticization		Calfskin biopsy	100 (RT)	100 (RT)	80 (Wet, RT)	14	150	[201]
Collagen/PU	H-bond + Plasticization		In-situ crosslinking	100	60	280 (Wet)	15	30	[202]
TPU	H-bond		Selective laser sintering	68 % (72 h wetting)	95 % (72 h wetting)	4–12 (Depending on wetting time)	0.75–2 (72 h wetting)	120–160	[168]
MPTC-DMAEMA-NaSS^h^	pH/protonation		One-step copolymerization + Casting	70 (0.5M HCl)	85 (0.5M NaOH)	N/A	0.05 (Wet)	≈630	[172]
Chitosan/PVA	Protonation of amine groups		Hot pressing + Co-precipitation	NR	94–96 (pH = 1, 3, 7)	NR	60–100 (RT)	5–110 (RT)	[213]
Silk Fibroin/MgO	H-bond + Hydrophobic-hydrophilic		Freeze-Thawing	NR	100	0.1 (Compressive, RT)	NR	∼65–75	[206]
*Dual-responsive Shape Memory Polymers*									
PEG-PCL	Water – Temperature (37 °C)	Crystal solvation + Crystalline	4D printing	∼90–95	∼85–90	1–1100 (Dry) 2–600 (Wet)(RT)	4–43 2–30	1–800 5–160	[237]
PTHF-PEG	Water – Temperature (37 °C)	Crystal solvation + Crystalline	Molding	75	NR	∼2.5 (RT)	NR	500	[236]
PLA-PEG-PLA	Water – Temperature (37 °C)	Crystal solvation + Crystalline	Solution casting	98.2	99.5	72.1 (Dry) 1.1 (Wet)	6.5 1	834 142.8	[235]

a This scaffold was used at body temperature (37 °C) to fix irregular bone damage sites.

b Starch-graft-poly(2-(methacryloyl)ethyl trimethyl ammonium chloride).

c 3-and 4-hydroxybutyric acid copolymer**/**PEG/ethiodized poppyseed oil/triamcinolone acetonide (30:5:1:10).

d The sample was made into a spiral shape with an initial diameter of 3.2–3.5 mm. The reported number is the increase in diameter after 28 days in the bile duct of a miniature pig.

e 3-(methacryloylamino) propyl trimethylammonium chloride-sodium *p*-styrene sulfonate-methacrylic acid.

f The degree of recovery from the twisted sample in 200 s.

g Poly(acrylamide-acrylic acid-2-(N,N-dimethyl amino)ethyl methacrylate).

h 3-(methacryloylamino) propyl trimethylammonium chloride-2-(dimethyl amino)ethyl methacrylate-sodium *p*-styrene sulfonate hydrate.

The advent of more accessible VP technologies, such as liquid crystal display (LCD) 3D printers, has further democratized high-resolution 3D printing [281,282]. LCD printers are typically more affordable and user-friendly, making them accessible to a broader range of researchers and clinicians aiming to design and produce SMP devices. Advancements in VP 3D printers now include heated vats, which allow the use of SMP inks with higher viscosities that become less viscous at elevated temperatures. Heated vats also enable the processing of SMP resins with longer chain structures or higher MW, which are often desirable for their strengthened mechanical properties. Moreover, new LCD printers offer larger printing areas, bringing them closer to mass-production fabrication methods such as molding techniques. This scalability addresses the traditional limitations of 3D printing (e.g., limited build volume) and opens possibilities for producing larger SMP-based structures or multiple units simultaneously. Combining high resolution with increased scalability further positions SMP-based 4D printing as a practical and efficient technology for clinical translation [112,113]. An additional advantage of 4D printing of SMP structures is its automation and the reduced requirement for specialized training. The automation inherent in 4D printing reduces the potential for human error and variability, enhancing the reproducibility and reliability of the fabricated devices.

Emerging VP techniques, including dual-wavelength printing [283,284] and grayscale printing [285], further enhance material control. Dual-wavelength VP enables selective crosslinking by using different wavelengths of light, allowing for multi-material integration and functionally graded structures essential for complex scaffolds. Grayscale printing complements this by varying light intensity within the printed layers, creating mechanical and degradation gradients within a single build. Together, these approaches broaden the scope of 4D printing in developing customizable, body-responsive SMPs tailored for biomedical applications. In addition to these techniques, researchers have recently developed novel VP methods that could be utilized for the 3D printing of SMPs. Layerless VP techniques, such as tomographic volumetric 3D printing [286,287] and Xolography 3D printing [288,289], are emerging methods capable of fabricating complex structures within a vat filled with a viscous material in less than 1 min. However, these methods are currently limited to printing structures that are generally smaller than 1 cm^3^, making them potentially suitable for medical devices such as stents and delivery devices. Furthermore, extensive research has focused on reducing the separation forces during printing by employing techniques such as oxygen-permeable Teflon films in continuous liquid interface production (CLIP) [290,291], mobile liquid interface printing in high-area rapid printing (HARP) [292], and air-liquid boundaries in dynamic interface printing [293]. Adopting these advanced VP methods for SMPs could significantly reduce fabrication times and broaden the range of materials employed, thereby enhancing the applicability of 3D-printed SMPs in fabricating cell-laden constructs.

### 5.2 Molding technologies

Beyond 3D printing, molding techniques have been extensively employed in fabricating body-responsive SMPs. Molding allows the fabrication of bulk materials with precisely controlled properties and is not remarkably limited by the viscosity of the SMP, offering greater flexibility in material design. The adaptability of molding makes it particularly advantageous for developing implants and devices responsive to physiological stimuli, as it enables fine-tuning material characteristics essential for in vivo applications.

PGD was molded into elastomeric devices via thermal curing under vacuum at elevated temperatures, demonstrating one of the earliest biomedical applications of PGD as a biodegradable thermoset elastomer [105]. Their work highlighted how the degree of thermal curing directly influences PGD's mechanical properties, enabling tunable stiffness and elasticity to match soft tissue mechanics. A minimally invasive PGD scaffold with transition temperatures between 27.8 °C and 36.6 °C was recently developed for nucleus pulposus regeneration by adjusting synthesis parameters to optimize [49]. They employed a hybrid fabrication method by molding the material into a disc shape and then using a laser cutter to pattern the final scaffold (Fig. 19C). These studies exemplify how thermal curing molding techniques can produce SMPs with tailored thermal responsiveness suitable for specific biomedical functions. Extending the application of molding, the percutaneous delivery of PGD devices in porcine pulmonary arteries was explored by molding PGD into geometries suitable for catheter deployment [116]. The devices expanded within the arteries and maintained patency over 12 weeks. By employing a different approach, thermal and magnetic dual-responsive microrobots were developed via molding (Fig. 19D) [101]. The microrobots were molded into linear forms by combining an organogel matrix with magnetic particles. Upon exposure to body temperature, they rapidly transformed into helices, navigating vascular pathways in response to magnetic fields. This innovative use of molding demonstrates the capability to produce devices with complex functionalities, combining thermal responsiveness with magnetic actuation. It highlights the adaptability of molding techniques to incorporate multifunctional properties into the SMPs. However, controlling the microrobots with magnetic fields introduces complexity and may limit accessibility due to the requirement for specialized equipment and precise control mechanisms.

Traditional molding techniques often face limitations in producing complex geometries, potentially hindering applications that require intricate, patient-specific designs. This poses challenges for personalized medicine, where customization of implants and devices is crucial for optimal patient outcomes. Molding processes are generally more labor-intensive and require significant hands-on skills, making them less accessible and more time-consuming than automated 4D printing methods.

### 5.3 Other fabrication methods

To overcome the limitations associated with both 4D printing and traditional molding techniques, advanced fabrication methods such as foaming [48], electrospinning [155], melt blending [31], and emulsion [102] techniques have been explored by different research groups. These methods enable the fabrication of SMPs with complex architectures and tailored functionalities, enhancing cell infiltration, tissue integration, and therapeutic outcomes.

Foaming techniques allow the production of SMPs that conform to irregular wound geometries. Fabrication of hemostatic shape memory polymer foams based on this technique yielded improvement in survival rates in lethal traumatic hemorrhage models [48,259]. The fabricated foams expanded rapidly upon exposure to body temperature and moisture, conforming to wound cavities and aiding hemostasis. Precise control over the foam's expansion rate is crucial to prevent potential tissue damage. Additionally, ensuring consistent performance across varying wound environments remains challenging. Utilizing the particulate leaching method, a PGA-based shape memory porous scaffold was developed by crosslinking PGA with PCL-diols (Fig. 19E) [54,121]. However, the limited control over pore size distribution and interconnectivity inherent in particle leaching could affect cell infiltration and nutrient diffusion. Moreover, significant stem cell death and detachment were observed during the shape memory activation process, suggesting that the mechanical stresses during deformation may impede tissue regeneration. While cells were able to proliferate after shape recovery, further optimization is needed to control the mechanical properties and pore architecture to enhance cell viability and function.

Electrospinning is commonly used for fabricating fibrous structures resembling the extracellular matrix, promoting cell attachment and proliferation [260,266]. Electrospun scaffolds were developed from a biobased triblock copolymer, modulating the shape memory behavior through thermal annealing [155]. They adjusted the polymer's crystalline domains by annealing the electrospun scaffolds at different temperatures, thereby tuning the shape recovery temperature from approximately 25 °C–50 °C. This technique offers the capability to create large-diameter conduits with a large palette of polymers that can be implemented in the fabrication [189,294,295]. The SMP nanofibrous structure has been extensively used in designing nerve guidance conduits. Although electrospinning offers advantages in creating nanofibrous architectures, the variability in the fiber diameter and pore size may affect cell infiltration and tissue integration. Moreover, the lack of structural complexity compared to 3D-printed constructs may limit the application of this technique for load-bearing or geometrically demanding scenarios. Scaling up production while maintaining consistency also remains a challenge for creating electrospun SMPs.

Emulsion techniques have been utilized to create porous SMPs with geometrically adaptive properties. Porous SMPs were fabricated using an emulsion lyophilization technique combined with a two-stage curing process, allowing post-fabrication customization and overcoming the limitations of traditional mold-based methods (Fig. 19F) [102]. This approach enabled the formation of highly interconnected porous networks, which are advantageous for nutrient diffusion and tissue integration in cell-laden implants. However, the mechanical properties of fabricated SMPs may not suffice for load-bearing applications, and the complexity of the fabrication process may hinder scalability. Further optimization of the emulsion parameters and curing kinetics may be necessary to improve reproducibility and mechanical strength across larger device formats. In a recent study, highly porous PCL-based SMPs were fabricated using a high internal phase emulsion technique [122]. The fabricated vaginal stents created using this technique showed 70 % porosity with an average pore size of 30 μm. Such porosity makes the structure highly compressible without mechanical failure, facilitating the design of body-responsive self-expanding devices for minimally invasive delivery.

The choice of fabrication techniques plays a critical role in determining the quality and clinical viability of body-responsive SMPs. Among these methods, 4D printing technologies, notably VP, offer unmatched resolution, automation, and design complexity, making them ideal for applications requiring intricate microscale features and rapid iteration. 4D printing is advantageous because of its reduced training requirement and faster design-to-iteration cycles, and its automated nature lowers the barrier to entry. It minimizes reliance on specialized skills, contrasting sharply with techniques like molding or electrospinning, which often require extensive hands-on expertise. The digital workflow in 4D printing facilitates rapid prototyping and easy design modifications without new molds or complex retooling. This agility is instrumental in personalized medicine, where patient-specific devices need to be developed quickly and precisely. However, 4D printing also presents challenges, such as the need for low-viscosity, photocurable SMP resins and potential limitations in material compatibility.

While molding techniques provide greater flexibility in material choice and control over properties, they often require intensive training and manual skills, which can slow down the development and may introduce variability. Molding is advantageous for bulk fabrication and is not as limited by polymer viscosity, making it suitable for many SMP chemistries. However, it can be less efficient for fabricating highly complex or customized geometries and is generally more labor-intensive than automated printing. In contrast, advanced 4D printing methods automate much of the process, improving consistency and reproducibility.

Other fabrication methods, such as foaming, electrospinning, and emulsion techniques, expand the potential of SMPs by enabling tailored functionalities. Electrospinning, for example, is well-suited for creating fibrous structures that mimic the extracellular matrix, which can promote cell attachment and proliferation, but scaling up and maintaining uniform fiber morphology can be challenging. Foaming allows for the fabrication of highly compressible and expandable SMPs, beneficial for minimally invasive delivery, though precise control over expansion and uniformity remains a challenge. Emulsion techniques can yield highly porous scaffolds, improving nutrient diffusion and tissue integration, but may present difficulties in achieving mechanical strength and process scalability. These approaches often demand specialized equipment and expertise, potentially limiting their accessibility [49]. Overall, each fabrication technique offers specific strengths and limitations, and the optimal choice depends on the intended biomedical application, required device complexity, and desired material properties. Novel molding techniques, such as micro-molding or micro-injection molding with biodegradable SMPs, may also support the fabrication of complex geometries while preserving material properties. As the trend toward accessible and automated fabrication methods like 4D printing continues, advancements in materials science and technology promise to overcome limitations, enabling personalized biomedical devices with enhanced functionality and safety. Table 1 summarizes the body-responsive SMPs discussed in this review, organized by stimulus type and fabrication technique.

## 6 Clinical applications for body-responsive SMPs

Given their capability to be activated under physiological conditions, body-responsive SMPs have been studied for various biomedical applications, including bone repair, vascular intervention, cardiac repair, and wound healing [37,47,61]. Their versatility and potential for minimally invasive procedures make this category of SMPs well-suited for translation into clinical settings. In this section, different applications of body-responsive SMPs are reviewed, and their potential for future applications is discussed.

### 6.1 Skeletal and musculoskeletal

#### 6.1.1 Bone repair

Bone regeneration in irregular critical-size defects caused by infection, trauma, or tumor remains a significant challenge for surgeons and researchers [206,271,276]. Autografting is the gold standard in clinics for bone repair, yet nerve damage, new fractures, and persistent pain often accompany this approach. In open surgery, reshaping irregular bone defects for treatment can result in severe postoperative pain. A practical approach to address these challenges is to use the ability of body-responsive SMPs to conform to complex geometries. The primary advantage of body-responsive shape memory scaffolds over conventional scaffolds lies in their ability to be predesigned and deformed into a desired geometry, followed by shape recovery at the injury site. Several studies demonstrated the potential of SMP bone scaffolds embedded with biological cues, such as growth factors and bone minerals, to improve osteoconductive properties [168,261,269,272,276]. In addition, the intrinsic piezoelectric properties of bone modulate the cellular behavior, making the electrically active scaffolds an intriguing candidate for improved bone healing [90,274]. Piezoelectric ceramics have shown promise in bone regeneration, but their poor processability and brittleness limit their applications [90]. Incorporating them into body-responsive SMPs enables effective electrochemical energy conversion while addressing the limitations of ceramics, which allows conformal fitting into irregular defects, enhancing scaffold osseointegration. This result was achieved by chemically crosslinking barium titanate nanoparticles, functionalized with dopamine and Ag, into AESO as a body-responsive TMP. The printed scaffold exhibited enhanced cell attachment and proliferation following ultrasound stimulation, which polarized the scaffold and improved its piezoelectric activity. The implanted scaffold in a rat cranial defect (Fig. 20A(i**)**) significantly outperformed the AESO and non-polarized scaffolds, showing a higher rate of new bone regeneration. The polarized scaffold enabled continuous electrical stimulation in vivo, achieving rapid cranial bone regeneration within 8 weeks by maintaining structural integrity (<10 % degradation at 60 days). This optimal degradation profile sustained mechanical support throughout the critical 8-week bone healing period while permitting progressive neotissue formation, fulfilling both the temporal and functional requirements for successful cranial defect repair [50,206].

The use of body-responsive SMPs has been limited to non-load-bearing bone regeneration due to their inherent mechanical deficiencies, and particularly their tendency to undergo biodegradation under physiological conditions [206,269,271]. One straightforward strategy is to design scaffolds with slightly higher *T*_*trans*_, which are known as *self-fitting* scaffolds [50,54,271]. The marginally higher *T*_*trans*_ helps the structure preserve its molecular orientation to achieve better mechanical properties. Although these scaffolds have shown better mechanical properties, they suffer from the long deployment time within the body. Therefore, external heating modalities, such as near-infrared radiation or ultrasound, must be used to increase the temperature locally to their transition temperature. Another promising approach to compensate for the mechanical degradation is to incorporate body-responsive switching segments into another segment that is resistant to physiological conditions. Using this technique, PGA-g-PCL segments with a *T*_*trans*_ of 37 °C were used as the switching segments in poly(ω-pentadecalactone) crystalline domains with a *T*_*trans*_ of 60–80 °C [54,125]. This strategy addressed the need for implants in alveolar bone regeneration, where the loading is constant over time. The molded SMP was also coated with dopamine and Ag to introduce antibacterial properties. A body-responsive scaffold that was used to fill the alveolar socket of rabbit models (Fig. 20A(ii)) showed successful bone regeneration as compared to a mandibular bone without a scaffold for two months post-implantation. Micro-CT scans clearly showed bone regeneration after the surgery. This approach can be further expanded to aid in repairing defects in load-bearing bones, such as the femur, tibia, and spinal vertebrae, by coupling with advanced fabrication techniques such as 4D printing [296,297].

#### 6.1.2 Cartilage repair

Articular cartilage defects are one of the most widespread diseases globally [51,111]. Cartilage has an intrinsic deficiency in self-regeneration and lacks blood vessels, nerves, and a lymphatic system, making treatment of defects remarkably challenging. While there has been considerable progress in cell therapy for cartilage repair using injectable hydrogels, the fabrication of hydrogels with superior mechanical performance remains a challenge, hindering their effectiveness for minimally invasive procedures, such as arthroscopy. Cell-free body-responsive SMPs, however, show excellent potential due to their better mechanical properties and stability [51]. In vivo studies on rat femur condyles show no noticeable difference between normal cartilage and the cartilage defect having a body-responsive scaffold embedded with tannic acid and Kartogenin. Although the study demonstrated body-responsiveness in the prepared scaffolds, this response was not utilized in the in vivo experiments, highlighting the need for further validation. Furthermore, based on the nature of cartilage function and the intensive research conducted on injectable hydrogels [298,299], the use of WMPs reinforced with hydrophobic crystalline domains seems to be a promising approach for cartilage repair. Cell-derived factor delivery using SMPs could also be considered as a potential alternative. In a recent study, body-responsive TMP structures made of PGD linked to chemokine stromal cell-derived factor 1α (SDF-1α) were used to regenerate the disc nucleus pulposus [49,300]. The fabricated scaffold was delivered to the rabbits as a rod and remodeled into a tendril structure, filling the disc cavity stimulated by body temperature. The in vivo studies showed that the scaffold maintained the disc's height and triggered the stem cells to migrate and regenerate the tissue through the controlled release of SDF-1α from the scaffold over 16 weeks. As shown by the X-ray in Fig. 20A(iii), the PGD + SDF-1α scaffold maintained about 83 % of the disc height during the 16-week window of the study, while the nucleus pulposus injury was reduced to 53 % as compared to an intact disc. PGD had a similar trend up to eight weeks, but the height gradually decreased from 78 % to 65 % over a 16-week period due to the degradation of the scaffold from weeks 8 to 6. While this new treatment for disc regeneration is considered a significant step forward in cartilage repair, more long-term studies on scaffold stability are needed, given the long-term disc regeneration window.

#### 6.1.3 Artificial muscles

Artificial muscle biofabrication is an emerging field of research utilized in conjunction with biorobots and implants for applications in tissue regeneration [[301], [302], [303]]. Recent research has been focused on the engineering of porous and fibrillar constructs that can mimic the structure of native muscles [304]. Most skeletal muscles in the body are arranged in pairs with antagonistic actions, and the muscles exhibit different mechanical properties during contraction and stretching [301,305]. For this reason, a hydration and dehydration process for WMPs is considered a biomimicry function for the design of artificial muscles [218,305]. For example, fibers made of PVA, acrylic acid, acrylamide, and tunicate cellulose nanocrystals showed different elastic moduli in hydrated and dehydrated states, indicating their potential for use as muscle-mimicking constructs [305]. In a similar study, PCL was used as a block copolymer with perfluoropolyether (PFPE) to fabricate a TMP that mimics the mechanical properties of muscle tissues [306]. The structure showed excellent biological properties that were similar to previously reported results on conductive PCL scaffolds for skeletal muscle repair [273,306]. The dynamic responsiveness of these SMP scaffolds would enable prosthetic devices that can control precise motion while promoting skeletal muscle repair. Dual-responsive SMP scaffolds are great candidates for accommodating artificial muscle functions, where thermally responsive segments enable cyclic stretching and contraction [306], while the water-responsiveness allows modulation of mechanical properties through controlled hydration [305].

### 6.2 Cardiovascular and adipose tissue

#### 6.2.1 Vascular embolization

Vascular embolization is a minimally invasive procedure that is used to intentionally block blood flow in vessels to clinically treat conditions such as aneurysms, tumors, hemorrhages, and vascular malformations [307,308]. Body-responsive SMPs play a transformative role in vascular embolization therapies through minimally invasive surgeries, as evidenced by the success of Shape Memory Medical, which has developed SMPs that are currently being tested in clinical trials [[309], [310], [311]]. Besides, researchers have investigated the adaptability of body-responsive SMPs for patient-specific treatments, as conventional designs are not compatible with all geometries of vessel abnormalities [100,119]. Body-responsive SMPs have been investigated using 4D-curable organogels for the fabrication of microcoils to treat aneurysms [100]. This strategy enables the researchers to create patient-specific embolization plugs using molding and 3D printing technologies. As shown in Fig. 20B(i), microcoils delivered via a microcatheter successfully filled an aneurysm model in a rabbit within 40 s after exposure to the animal's blood temperature (39.5 °C). The stereomicroscope images collected 1 month after implantation showed that the microcoils caused thrombosis, which stopped blood flow to the aneurysm. Although the reported results showed excellent adaptability for creating patient-specific devices, the fabrication process could benefit from a shift to 4D printing techniques. As the current approach is time-consuming and material-intensive, it involves using both 3D printing and injection molding. Another application for SMP microcoils is to adapt them for treating hard-to-reach vessels, such as cortical arteries. Incorporating magnetic particles into TMPs has enabled researchers to use magnetic actuation systems to transfer the microcoils to the targeted regions [101].

#### 6.2.2 Vascular grafts

Vascular transplantation is the conventional practice for treating cardiovascular diseases that are mainly initiated by endothelial cell dysfunction [182,260]. However, specific issues, such as a shortage of donors and the rejection of donor tissues by the immune system, hinder the development of this technique. Body-responsive SMPs in this scenario are promising alternatives owing to their ability to conform to a tubular shape [123,182,260,250]. This strategy facilitates endothelialization on a two-dimensional planar surface, which can transform into a 3D circular scaffold on the intended lumens. Despite excellent in vitro results [208,283,290], current literature lacks sufficient promising in vivo data to consider SMP vascular grafts in minimally invasive procedures within clinical settings. However, body-responsive cell-free patches fabricated using PGD and PCLUSe showed remarkable performance in vitro and in vivo [116,152]. In a recent study, PGD patches were delivered percutaneously through the catheter into the porcine pulmonary artery [116]. The implanted scaffolds in pigs showed significant degradation after three months while supporting cell growth without a substantial inflammatory response. Complete tissue coverage was observed in 5 out of 8 tested models after three months, while the rest had partial tissue coverage. In another study, the successful delivery of PCLUSe self-healable scaffolds was demonstrated [152]. Rolled SMP segments of a large patch were delivered through a 10-mm trocar to a dog (Fig. 20B(ii)). The segments successfully opened at body temperature, and a 405-nm irradiation was used to patch the spliced segments together. Although a low inflammatory response was shown in small animal models, this study lacked biological studies on the canine model. Further improvement regarding tissue regeneration can be achieved by using cell-laden patches.

#### 6.2.3 Adipose repair

Adipose tissue defects typically present considerable challenges in reconstructive and plastic surgery; in this context, adipose tissue engineering aims to generate native-like fat as soft tissue substitutes [312,313]. Damage to adipose tissue, whether due to disease, trauma, or surgical procedures, often necessitates soft tissue reconstruction, particularly in areas like the breast and other subcutaneous regions [313]. Multiple research studies have focused on adipose-derived stem cell aggregates given their potential for adipogenic differentiation and secretion of angiogenic growth factors. Despite the growing research on porous body-responsive SMPs [109,117,191,253], these scaffolds have yet to be thoroughly explored for adipose tissue regeneration. For the first time, researchers designed a low-fouling coated TMP scaffold for vascularized adipose repair to reduce the risk of infection [125]. The scaffold, which was discussed in Section 2.1.2, was fabricated from PGA-g-PCL/PPDLA and modified with PEG-conjugated dopamine coating to achieve a low-fouling surface. The coating was found to enhance cell infiltration and induce minimal immune response, as evidenced by analysis of subcutaneous implantation in mice. In another in vivo study, scaffolds seeded with adipose-derived stem cells were implanted through injection into the dorsum of nude mice and heated to 43 °C using infrared radiation to recover to the original shape. After 12 weeks, the study groups remained unchanged due to their slow degradation, while gross images exhibited vessels around the scaffolds (as shown in Fig. 20B(iii)). Hematoxylin and eosin (H&E) staining also revealed blood vessel formation in both groups. A large vacuole in the H&E image and the ring-like morphology for the experimental group confirm the formation of adipose tissue. These results, for the first time, demonstrate the potential of low-fouling TMPs for adipose tissue engineering. A possible improvement to this work is to adjust *T*_*trans*_, which was at 43 °C, to match body temperature. Based on previous studies, polyesters, their copolymers, and PCL-based polyurethanes show potential for use as body-responsive SMPs for fat and soft tissue engineering [118,237,252].

### 6.3 Wound healing and skin repair

The urgent need for effective hemorrhage control has spurred the development of innovative wound-healing approaches [234]. SMP foams have emerged as a promising hemostatic agent in this regard. They exhibit biocompatibility, rapid clotting, and the ability to conform to the wound site's geometry [48,97,98]. Owing to their low stiffness, these foams can be delivered to complex wound sites without causing further tissue damage. Fig. 20C(i) shows a photographic comparison of SMP foams with the commercial hemostatic dressings XStat® and QuickClot® used for a Grade V porcine liver model [48]. The SMP foams decreased the active bleeding time from approximately 29 min and 27 min for XStat® and QuickClot®, respectively, to 21 min. Treatment with SMP foams also resulted in about 11 % lower mean blood loss. Collectively, SMP foams increased the 6-h survival rate by 37 % and 50 % compared to XStat® and QuickClot®. A recent study showed that incorporating vanillic acid into SMP foams stabilizes bleeding through its antimicrobial and pro-coagulant properties [259]. However, further studies should be carried out with a larger number of animal models to better understand the clotting mechanism. In a related study, researchers designed sponges based on quaternized chitosan and chitosan with antibacterial properties and observed a promoted wound healing cascade [199]. The sponges achieved blood clotting in a liver injury model about 20 s faster than those using commercial gelatin sponges. In the full-thickness skin defect model shown in Fig. 20C(ii), the use of body-responsive SMP sponges resulted in 90 % faster wound healing after 12 days compared to the group without any dressing. Due to their physical crosslinking, SMP sponges showed rapid dissolution on the surface, resulting in more convenient dressing detachment for acute wounds. Given their antibacterial properties and rapid clotting, these sponges also have great potential for deep wounds. The excessive exudate in the wound site has led to the design of water-responsive patches for skin repair [192,202,275]. In a recent study using this approach, a polyurea fibrous membrane was employed as an active dressing for wound repair [192]. As shown in Fig. 20C(iii), the SMP structure exhibited 53 % healing after 3 days. The healing rate in the first few days following trauma is critical as this period involves an inflammatory phase that directly influences tissue proliferation, granulation, and the restoration of skin continuity. After 12 days, no significant difference was noted between the groups. Another similar study used PU and collagen as a WMP dressing for active wound repair [202]. This strategy can be coupled with body-responsive TMPs to design programmed bandage-like scaffolds to facilitate wound healing. Such constructs can be incorporated into phase-specific drug release, given their dual-responsiveness. Delivering antimicrobial agents upon application and releasing regenerative factors over time may potentially promote tissue repair and re-epithelialization.

### 6.4 Nervous and sensory systems

#### 6.4.1 Neural repair

Peripheral nerve injuries pose a significant clinical challenge, as they have a high disability rate in patients and a complex regeneration process [294,295]. Autografts are regarded as the gold standard for repair, yet their application is limited by donor tissue scarcity, risk of infection at the harvest site, and high treatment expenses. Such challenges have prompted researchers to develop artificial nerve conduits for nerve repair. SMP implants are gaining attention in neural tissue engineering because they comply with minimally invasive procedures, provide structural support, and promote nerve regeneration [189,294,295,279,314]. One of the earliest biomedical applications of SMPs involved a smart tubular conduit capable of gradual shortening while the nerve regenerates [189]. However, single tubular conduits cannot mimic the anisotropic structure of the nerve and may lead to nerve misorientation [189,294]. To address this limitation, multichannel SMP conduits have demonstrated superior axonal guidance and functional recovery by mimicking nerve fascicles [294,295]. Although body-responsive SMPs are mainly recognized for their potential in minimally invasive procedures, their application in fabricating nerve conduits is also promising due to their ability to conform to tubular geometries. Seeding cells on planar SMP constructs and changing their shape to multichannel conduits is a more straightforward approach than the direct fabrication of cell-seeded multichannel conduits. Based on this design, planar sheets were electrospun with PLLA-PTMC and gelatin to incorporate shape memory property and hydrophilicity into the conduits simultaneously. Graphene oxide (GO) and reduced graphene oxide (RGO), embedded into the SMP constructs to enhance electrical conductivity, were found to accelerate nerve regeneration [295]. The harvested nerve conduits from rats at 12 weeks post-surgery are shown in Fig. 20D(i). Nerve cross-sections were evaluated for axonal regeneration, toluidine blue staining revealed a high density of Schwann cells in all groups, with the SMP RGO showing a similar density to the autograft group and significantly better density than the other groups. Although the inflammatory response during subcutaneous implantation decreased after 4 weeks post-surgery, this initial response may still pose challenges for nerve regeneration, highlighting the need for more studies on the effect of this SMP in neural repair.

#### 6.4.2 Bioelectronic devices

Body-responsive SMPs are key components in developing bioelectronic interfaces and flexible devices due to their adaptability and configurability [208,265,315]. The use of SMPs facilitates the creation of flexible microelectrode arrays, nerve modulators, and monitoring devices [178,208,265,268,315]. For example, microelectrode arrays made of SMPs can self-fold for minimally invasive implantation [265]. The device can accommodate large strains and geometrical curvatures, enabling it to be used as an ocular prosthesis to improve visual field and acuity [265]. Furthermore, the adaptability and recovery of body-responsive SMPs show promise for the design of twining electrodes for neural stimulation and recording [178,208,268]. Recently, researchers utilized silk fibroin to design adaptable bioelectronic implants [208]. The device was designed based on pristine silk fibroin, a WMP membrane that can be recovered into a twining electrode shape in 10 min. Fig. 20D(ii) depicts a photograph of an electrode wrapped around a rat's vagus nerve. The shape memory electrode maintained its integrity over a month after implantation in rats, with no significant damage observed. The electrode successfully stimulated the vagus nerve, which in turn resulted in better heart rate modulation. These silk-based electrodes are limited by their short lifespan, as they fully degrade in the protease K solution in 24 h. This shortcoming can be addressed by incorporating silk into synthetic SMPs to tune the degradation. In a similar study, researchers designed tissue interfaces based on SMPs to fabricate a triboelectric nanogenerator used in the real-time monitoring of ureteral peristalsis to assess bladder volume [315]. The implantable device was used to diagnose urological disorders such as overactive bladder, underactive bladder, and other urinary-affected disorders. The device was implanted in a pig ureter (Fig. 20D(iii)), and it collected signals to monitor the bladder volume. Owing to the use of SMPs, this method is less invasive and poses a lower risk of infection than traditional methods such as catheterization. Body-responsive SMPs also offer unique advantages for incorporation within wearable devices, such as pressure [316] and movement sensors [317,318], enabling the design of substrates with tunable stiffness and reconfigurable shapes, improving skin conformity and user comfort [114,319]. The temperature- and water-responsiveness can support miniaturized, shape-adaptive electronics that render adjustability to physiological conditions. Body-responsive SMPs, therefore, can enhance the performance of wearable health monitoring systems.

### 6.5 Drug and cell delivery

The compressibility of body-responsive SMPs into compact forms has driven interest in their use for miniaturized drug delivery systems [128,235,236,251,257,320]. These structures can enhance drug adsorption by increasing the local concentration of a drug at the target site. Typically, gastrointestinal drug delivery devices need to be compressed into a capsule to prevent drug release in the stomach, as biomacromolecules are susceptible to degradation under strongly acidic conditions [251,320]. As shown in Fig. 20E(i), the 4D-printed SMP was compressed in a gelatin capsule and was fully recovered 17 min after the capsule was dissolved. The SMP was fully degraded within 6 h in simulated intestinal fluid. Incorporating in vitro and in vivo drug release studies would further strengthen the potential of such SMPs for translational applications.

Recent studies have worked toward replacing conventional balloon-assisted stents with drug-eluting body-responsive SMP stents [115,235,236,251,257]. These stents can release drugs upon delivery to the targeted site, enabling dual functionality. The drug-eluting SMP stents could become a practical approach for treating esophageal cancer [235,236]. Compared to conventional metal alloys, SMPs can be degraded, which would eliminate the need for removal after treatment. A novel dual-responsive SMP using PEG-PLA copolymer was designed for this application [235]. As shown in Fig. 20E(ii), the SMP stent can be implanted radially in a glass tube and then expanded using temperature and water as sequential stimuli, as the cotton in the tube resembles the tumor in the esophagus. The stent can be placed in close contact with the area restricted by the cotton and then release the drug into the cotton, as indicated by the area shown in yellow. Future studies can explore more biological experiments to validate the clinical use of these stents.

The shape-changing capability of body-responsive SMPs positions them as promising candidates for cell carriers in minimally invasive procedures [7,121,231]. Porous SMPs are particularly advantageous, as their architecture mimics a cell-friendly microenvironment. However, a critical concern is the cell viability during shape-programming (e.g., mechanical deformation) and recovery. In a study, it was reported that programming and recovering steps in cell-seeded constructs resulted in a drop of 32 % in cell viability due to the applied shear stress [121]. Notably, despite the dramatic decline of cell viability, the viable cells continued to proliferate. As shown in scanning electron microscopy images in Fig. 20E(iii), the cells (marked with yellow arrows) spread and adhere to the porous structure. Although most pseudopods were observed to retract during the shape recovery phase, they re-emerged after one day of incubation with no change in cell proliferation, as shown in the fluorescent microscopy images in Fig. 20E(iii). The integration of cell-laden SMP and bioprinting techniques further enables the biofabrication of intricate shape-changing constructs for minimally invasive delivery. Additionally, body-responsive SMPs also exhibited the ability to conform to specific topological surface patterns to control cell differentiation [258,263,264]. Such patterning can be utilized in conjunction with SMP designs for nerve regeneration, cardiac repair, and tissue modeling, where spatial cues may govern cell phenotype and enhance the regeneration process [7,146,262].

### 6.6 Other applications

Recent advancements in body-responsive SMPs have driven the progress of innovative medical devices with improved functionality while maintaining biocompatibility and biodegradability. For example, in a recent study, a drug-releasing SMP stent was designed to treat biliary strictures (Fig. 21A) [280]. Experiments on miniature pigs proved the therapeutic effectiveness of the stents in maintaining an unobstructed biliary duct and effectively inhibiting tissue hyperplasia. Similarly, a body-responsive SMP was utilized for 4D printing of an inferior vena cava filter capable of preventing pulmonary embolism by trapping thrombi, as illustrated in Fig. 21B [112]. This advancement offers a clinically feasible approach for the design of such filters. Beyond these applications, researchers further expanded the applications of SMPs by designing ultrathin membranes for retinal tissue engineering, highlighting the potential use of SMPs in ophthalmology for the first time [277]. This ultrathin membrane can be delivered through a catheter or syringe, as shown in Fig. 21C. SMPs were also used as a self-fitting vaginal stent (Fig. 21D) developed to prevent vaginal stenosis following pelvic radiation and reconstruction [122]. The shape change in SMP stents enabled convenient insertion and the maintenance of vaginal patency for secretion drainage. Additionally, remote-controlled SMPs have been explored for urethral stenting, demonstrated in Fig. 21E [151]. High-intensity focused ultrasound was found to enable precise, non-invasive shape recovery of deep tissue stents without inducing hyperthermic damage for stent retrieval, as demonstrated in a live canine bladder model. These advancements collectively illustrate the versatility of body-responsive SMPs beyond conventional applications, showcasing a diverse and evolving field of study. Using SMPs in the biomedical field can potentially revolutionize treatments or introduce new approaches through minimally invasive procedures. Table 2 summarizes the applications of body-responsive SMPs with their biological properties.

**Table 2:** Application and biological performance of body-responsive SMPs. The table is organized by the applications of referenced SMPs throughout the manuscript, along with the corresponding tissue. The degradation is reported in mass loss percentage.

Application	Trigger	Polymer Network	Deployment Time	In vitro Degradation	In vivo Degradation	Cell/Animal Studies	Ref.
*Skeletal and musculoskeletal*							
Cranial defect	Water/Blood	Silk fibroin/MgO	<1 s (Water) <10 s (Blood)	60–70 % weight loss within 28 days in simulated body fluid.	Partial degradation after 8 weeks in the rat cranial defect.	Indirect cytotoxicity on mouse embryo osteoblasts.	[206]
Alveolar bone	Body Temperature	PGA-g-PCL – PPDLDA	NR	NR	∼32 % weight loss subcutaneously within 3 months in rats.	Direct cytotoxicity with bone marrow stem cells.	[54]
Bone grafts	Body Temperature	PLA-POSS-Hydroxyapatite	NR	Complete degradation in PBS after 4 months.	NR	Proliferation and osteogenesis study via culturing bone marrow-derived stromal cells.	[269]
Long bone defect	Body Temperature	PLGA-PTMC	660 s	NR	NR	Regular human osteoblast cell activity.	[135]
Femoral defect	Body Temperature	PCL-HDI-castor oil-hydroxyapatite	60 s	NR	No remarkable degradation 12 weeks after surgery in rabbits.	Cell proliferation and infiltration with mouse preosteoblasts.	[271,272]
Cranial defect	Body Temperature	AESO	300 s (37 °C) 180 (45 °C using IR)	Weight loss <2 % within 3 weeks in lipase solution.	Weight loss of <5 % within 60 days in rats	Preosteoblasts co-cultured with fabricated scaffolds.	[90]
Bone (Potential)	Body Temperature	AESO	NR	NA	NA	Co-culturing with human mesenchymal stem cells (hMSCs).	[88]
Cranial defect	Body Temperature	PCL/AT:IPDI:CCYS	60 s	∼30 % and ∼60 % weight loss in 8 weeks in PBS and glutathione media, respectively.	NR	Human adipose-derived stem cells were cultured on scaffolds.	[274]
Bone (Potential)	Body Temperature	PCL Dimethacrylate	NR	NR	NR	Cytocompatibility with mouse fibroblasts	[266]
Mandibular bone defect	Body Temperature	PCL-hydroxyapatite	60 s	Depending upon the pore size, 5–12 % within 12 weeks in PBS.	NR	The cytotoxicity test was performed on rabbit bone marrow stem cells.	[276]
Barrier membranes for guided bone regeneration	Body Temperature	PLLA-PTMC	12 s (39 °C)	NR	NR	The cytotoxicity test was performed with primary osteoblasts isolated from newborn mice.	[261]
Bone repair	Body Temperature	PCL	NR	Complete degradation in 0.1M NaOH media within 15 days.	NR	NR	[50]
Bone repair	Water	TPU	NR	NR	NR	Human osteosarcoma cells cultured on scaffolds.	[168]
Cartilage repair	Body Temperature	PUU (MDI:IU: PEG)	30 s	NR	NR	MTT assay with bone marrow mesenchymal stem cells.	[51]
Nucleus pulposus repair	Body Temperature	PGD	10 s	NR	About 90 % weight loss after 9 weeks in rats.	Cytotoxicity was tested with mouse fibroblasts.	[49]
Cartilage repair	Body Temperature	PGS-PPS-Kartogenin	NR	About 60 % weight loss in PBS within 12 weeks.	NR	Cytotoxicity was tested with bone marrow mesenchymal stem cells	[111]
Skeletal muscle	Body Temperature	PCL-AT	15 s	10–50 % degradation after 36 h in lipase media.	NR	Cytotoxicity with an immortalized myoblast cell line.	[273]
Tendon repair	Body Temperature	PCL-PEG	NR	About 5 % weight loss in PBS within 8 weeks.	NR	Human umbilical cord mesenchymal stem cells were used to study in vitro tenogenic differentiation. The rat Achilles tendon model was used for in vivo studies.	[146]
Volumetric muscle loss repair	Body Temperature	PCL-PFPE	NR	NR	NR	Co-culture with C2C12 myoblasts and in vivo studies in a mouse model.	[306]
Artificial muscle	pH	PMATC	NR	NR	NR	MTT assay with NIH 3T3 cells.	[218]
Artificial muscle	Water	PVA-Acrylamide-Ammonium Persulfate-Cellulose nanocrystals	NR	NR	NR	NR	[305]
*Cardiovascular and adipose tissue*							
Endovascular embolization	Body Temperature	Acrylate network	20–30 s	NR	NR	Cytotoxicity with red blood cells isolated from a rabbit's blood. In vivo studies on rabbits.	[100]
Vascular/aneurysm embolism	Body Temperature	Magneto-acrylate network	2 s	NR	NR	In vivo studies on rabbits.	[101]
Left-atrial appendage occluder	Body Temperature	PGDA-Polyacrylic acid	∼10 s	15 % and 45 % weight loss in PBS and NaOH solution, respectively, in 6 months.	The degradation in the mouse aorta was ∼12 % after 21 days.	Cytotoxicity with pluripotent mesenchymal progenitor.	[119]
Embolic sponges	Body Temperature	Acrylate emulsions	10 s	NR	NR	Cytotoxicity with mouse fibroblast and in vivo studies in Bama miniature pig.	[102]
Aneurysm occlusion/Vascular stents (Potential)	Body Temperature	PGDA	NR	NR	NR	Cytotoxicity with mouse fibroblast cells.	[109]
Vascular grafts	Body Temperature	PLLA-PTMC	NR	NR	NR	Cytotoxicity with human umbilical vein endothelial cells (HUVEC).	[250]
Vascular grafts	Body Temperature	PCL-Glycidyl methacrylate	NR	Almost intact after 28 days in PBS	NR	Cytotoxicity with HUVEC cells and in vivo studies on pigs	[123]
Cardiovascular repair patch	Body Temperature	PGD	NR	NR	Less than 30 % weight loss within 3 months in the porcine artery.	The in vivo studies carried out in Yorkshire pigs	[116]
Myocardial infarction	Body Temperature	PCLUSe	NR	40 % mass loss in a 100 mM hydrogen peroxide solution.	NR	Co-culturing with embryonic rat heart tissue for 3 days.	[152]
Cardiac repair	Body Temperature	AESO	60 s	NR	NR	hMSCs were used for attachment study and their differentiation into cardiomyocytes due to the micropatterns.	[89]
Vascular graft	Water	PVA/PCL/Chitosan	11.3–14.3 s	NR	NR	Co-culturing HUVEC and smooth muscle cells.	[182]
Vascular graft	Body Temperature	PCL/PLLA-PTMC/Gelatin methacrylate	∼8 s	NR	NR	Cytotoxicity with HUVEC cells.	[260]
Vascular graft	Body Temperature	PGDA	∼8 s	NR	NR	In vivo implantation into a mouse aorta	[115]
Soft tissue defects/vascular scaffolds/fibrous ring/cartilage	Water Body Temperature	PEG-PCL	∼60 s	NR	NR	Cytotoxicity with mouse fibroblasts. In vivo studies in rats.	[237]
Adipose tissue	Temperature (43 °C)^a^	PGA-g-PCL-PPDL	NR	NR	27 % weight loss after 3-month implantation in mice.	Cytotoxicity with adipose-derived stem cells. In vivo studies in mice.	[125]
Fat/soft tissue repair	Body Temperature	PLA-PEG-Cinnamic acid	110–175s	∼50 % weight loss after 12 weeks in PBS.	NR	Cytotoxicity with fibroblasts and osteoblasts. In vivo studies in rats.	[253]
Fat/soft tissue repair	Body Temperature	Acrylated PCL	NR	Complete degradation within 7 days in 5M NaOH solution	NR	Cytotoxicity with human adipose tissue-derived stem cells.	[55]
Fat/soft tissue repair	Body Temperature	PGDA	4.9–19.7 [rad.min^−1^]	20 % weight loss within 30 days in 0.1 mM NaOH solution.	NR	Cytotoxicity with NIH 3T3 cells.	[68,118]
Fat/soft tissue repair	Water	PFOT	150 s	∼24 % weight loss after 4 weeks in PBS solution.	NR	Cytotoxicity with embryonic rat heart tissue.	[191]
*Wound Healing and Skin Repair*							
Active wound healing	Water	Polydopamine-coated polyurea loaded with Ag nanoparticles	6 s	NR	NR	Wound healing in mouse models.	[192]
Hemostatic dressing	Water	CS/QCS	21–42 s	NR	No significant changes after 3 weeks in rats.	Study the blood cells' binding.	[199]
Hemostatic dressing	Body Temperature	PU	300 s	NR	NR	Cytotoxicity with NIH 3T3 cells.	[48]
Hemostatic dressing	Body Temperature	Vanillic acid incorporated PU	120 s	NR	NR	Cytotoxicity with NIH 3T3 cells.	[259]
Wound dressing	Body Temperature	PCL-PEG-AT	5–133 s	Almost complete degradation within 30 days in PBS with lipase.	NR	Cytotoxicity with L929 fibroblasts. In vivo studies in rats.	[275]
Wound dressing/artificial skin	Water	Collagen/PU	NR	5–50 % weight loss within 26 days. Depending on the ratio of collagen and PU.	NR	Cytotoxicity with hMSCs.	
Hemostatic agent	Body Temperature	PEG/PVP (90:10)	240 s	NR	NR	NR	[98]
Self-healing tissue scaffolds/wound dressing (Potential)	Water/Metal Coordination	PEG-modified dopamine	NR	NR	NR	Cytotoxicity with endothelial cells.	[233]
Wound closure device	Body Temperature	PLLA-PTMC (60:40)	NR	NR	NR	Human annulus fibrosus cells' collagen and fibronectin production.	[82]
Sutures (Potential)	Water	PEG/PCL/CNC	5 min	NR	NR	Cytotoxicity with osteoblast cells.	[177]
*Nervous and Sensory System*							
Neural brain repair	Water	Carbon nanotubes doped silk sericin	10 s	NR	Complete degradation within 12 weeks in murine brains.	Cytotoxicity with bone marrow MSCs isolated from wild-type mice.	[279]
Tubular nerve conduit	Water	PLGA	10 h^b^	NR	NR	Proliferation of Schwann cells.	[189]
Peripheral nerve regeneration	Body Temperature	PLLA-PTMC-Gelatin-GO	30 s	No mass loss after 60 days in PBS	NR	Cytotoxicity of Schwann and rat medullary pheochromocytoma (PC12) cells. In vivo studies on rats' nerve regeneration.	[295]
Peripheral nerve regeneration	Body Temperature	PLLA-PTMC	25 s	NR	NR	Cytotoxicity with Schwann and PC12 cells. In vivo studies on rats' nerve regeneration	[294]
Neural tube defect	Body Temperature	PLA-PCL	10 s	30 % weight loss in simulated human amniotic fluid after 100 days.	NR	Cytotoxicity with amniotic fluid-derived mesenchymal stem cells	[314]
Twining electrode as a nerve modulator	Water	Silk Fibroin^d^	15 min	80 % weight loss within 12 h in protease K solution (0.1 mg ml^−1^)	NR	In vivo studies on the right vagus nerve of rats.	[208]
Triboelectric nanogenerator for bladder monitoring	Body Temperature	PLLA-PTMC^d^	NR	NR	NR	Cytotoxicity with L292 fibroblast.	[315]
Nerve stimulator	Water	PEO/PEG-co-α Cyclodextrin	2 s	NR	NR	Cytotoxicity with normal human dermal fibroblast cells	[178]
Peripheral nerve stimulator electrode	Body Temperature	PCL-urethane (PCL diol reacted with isocyanate)^d^	NR	NR	NR	In vivo studies and implantation of electrodes in the vagus nerve of rabbits.	[268]
Retina stimulator for visual restoration	Body Temperature	PDLLA-grafted-polyacrylic acid/PEG	15 s	NR	NR	NR	[264,265]
Soft actuators/wearable electronics (Potential)	Body Temperature	Stearyl acrylate-Lauryl acrylate (75:25)	NR	NR	NR	NR	[113]
*Drug and cell delivery*							
Oral drug delivery device	Body Temperature	Polyaminoester-stearyl acrylate - PVP	17 min	Degradation in gastric, intestinal, and PBS solutions was complete after 3, 6, and 12 h, respectively.	NR	Cytotoxicity with Caco-2 cells as epithelial cells mimicking the small intestine gastrointestinal system.	[251]
Oral drug delivery device	Water + Body Temperature	PVA	10 min	NR	NR	NR	[320]
Drug delivery carriers	Body pH	PU (PEG-MDI-BIN(32 %))	NR	NR	NR	Cytotoxicity with osteoblasts.	[171]
Drug-eluting stent	Body Temperature	PLLA-PTMC (80:20)	90 s	Weight loss of <40 % within 10 weeks in PBS.	NR	Cytotoxicity with HeLa cells.	[84]
Drug-eluting stent	Water + Body Temperature	PTHFE-PEG	NR	NR	NR	NR	[236]
Drug-eluting stent	Water + Body Temperature	PLA-PEG-PLA	NR	No degradation within 24 h in PBS (pH = 2) and 45 % weight loss in PBS(pH = 7).	NR	NR	[235]
Intestinal Stent	Body Temperature	PLA/PEG (80:20)	NR	∼15 % degradation within 90 days in PBS.	NR	Cytotoxicity with Intestinal cells (DLD-1) and in vivo studies in mice.	[128]
Stent	Body Temperature	PCL-Isosorbide-castor oil	18s	NR	NR	Cytotoxicity with C2C12 skeletal muscle cells.	[257]
Cell encapsulation	Body Temperature	PGA-g-PCL	5 s (Wet) >600 s (Dry)	Weight loss of 41.6 % within 6 months in PBS.	∼95 % weight loss within 6 months in mice.	Cytotoxicity and differentiation with human adipose-derived stem cells.	[121]
Tubular Tissue scaffold/Cell encapsulation	Metal coordination with Zn^2+^	Vinyl imidazole-acrylonitrile	NR	NR	NR	Cytotoxicity with L929 Cells	[231]
3D-cell scaffold for blood-brain barrier model	Body Temperature	PLLA-PTMC	NR	NR	NR	Studied the morphological and functional properties of the brain barrier model	[262]
Cell substrate (Potential cell carrier)	Body Temperature	PDLLA-grafted-polyacrylic acid/PEG	NR	NR	NR	NR	[264]
Cell substrate (Potential cell carrier)	Body Temperature	Three-arm methacrylate PCL	NR	NR	NR	Cytotoxicity with hMSCs.	[258]
Cell substrate (Potential cell carrier)	Body Temperature	Diacrylate PCL/Tetra-acrylate PCL	2 h	NR	NR	Cytotoxicity with NIH 3T3 cells.	[263]
*Other Applications*							
Retinal tissue engineering	Body Temperature	PLCL/PLGA	60 s	NR	NR	Cytotoxicity with retinal pigment epithelial	[277]
Vaginal stent	Body Temperature^e^	Start-PCL	NR	Complete degradation in 4 weeks in PBS media with pH = 7.4 and 4.5.	NR	NR	[122]
Inferior vena cava filter	Body Temperature	PGSA-HEMA (55:45)	20 s	11.5 % weight loss within 8 weeks in PBS solution.	NR	Cytotoxicity with HUVEC cells.	[112]
Bile duct stent	Water	P(3HB-co-4HB)	96 h (PBS) 96 h (Bile) 52 h (Alcohol)	15–40 % mass loss in PBS, bile, and HCl within 6 months. The estimated complete degradation is 977, 425, and 1083 days, respectively.	NR	Cytotoxicity with NIH 3T3 fibroblast cells and mouse extrahepatic bile duct epithelial cells.	[280]

a The SMP was heated using infrared radiation.

b The time needed for the gradual shortening of the nerve conduit.

d Body-responsive SMPs have been used in a multi-layer structure and are responsible for changing the shape of the devices.

e Self-fitting vaginal stent used at body temperature with a *T_trans_* of 43 °C.

The developed body-responsive SMPs have shown great potential for medical use; however, the need for comprehensive biocompatibility studies is crucial. Considering Table 2, many studies lack in vivo evaluation of inherent SMPs' biocompatibility, and concurrent assessment of in vivo shape recovery behavior is particularly limited. There were only a limited number of studies [49,100,102,116,208,268,315] that assessed shape recovery and biocompatibility together. Additionally, the differences between cell types in in vitro studies hinder a comprehensive comparison.

As most of these polymers have been synthesized using organic solvents and catalysts, the complete removal of their residues is essential to ensure biocompatibility. This could be eliminated if a solvent- and catalyst-free procedure is used. The other issue is the unreacted moieties, such as acrylates, that have been widely used in UV-crosslinkable SMPs. Although the complete washing steps can eliminate this risk, using methacrylate groups can be a more feasible option [321]. Moreover, the thio-ene network offers an alternative way, often with better biocompatibility in cell- and protein-encapsulation networks that are less explored in SMPs [322,323]. The isocyanate precursors in designing degradable polyurethane-based SMPs are another risk, specifically the aromatic isocyanates such as MDI [324]. The degradation products containing these precursors contain aromatic amines, which are potent carcinogens. Literature recommends avoiding aromatic isocyanates in favor of aliphatic isocyanates. To design SMPs based on a distinct composition of polymers, the incorporation of FDA-approved polymers, such as PCL and PLGA, might be a feasible trajectory to be used in conjunction with non-approved polymers to pave the way toward clinical uses. Furthermore, using FDA-approved polymers combined with natural polymers can inspire confidence in practical, safe solutions for clinical translation due to their inherent biocompatibility.

Alongside the material considerations discussed, the maturity of the SMP technologies for translation can be assessed through the Technology Readiness Level (TRL) framework [325,326]. Most studies summarized in Table 2 can fall within TRL 3–5, representing stages where prototypes are developed and tested in relevant biological models, either in vivo or ex vivo. None of the studies can be classified at TRL 6 based on the presented data. Since TRL 6 is generally considered sufficient to begin early commercialization, meaning proven feasibility in a single human case. These studies indicate potential but require additional and thorough evaluation, which might be under consideration or investigation at the time of writing this review. The following section summarizes the FDA-approved products and ongoing clinical trials involving SMP-based devices that have progressed to TRL 7–9.

### 6.7 Clinical translation

Evidenced by in vitro and in vivo studies, there is an increasing body of work that supports the interest in utilizing SMPs for medical applications in clinical settings [49,100,151,295]. Despite these early-stage innovative studies on SMPs, there is limited research focused on clinical trials that bridge the bench-to-bedside gap and facilitate the market transition. Clinical practices have been led by a small number of companies. As noted before, the first successful commercialization of an SMP-based device was Morphix®, which is FDA-cleared as a permanent implant [327,328]. Following this innovation, Eclipse was introduced as another fixation device for bone based on an FDA-approved polyether ether ketone, both from Envovis, formerly MedShape. It is worth noting that both systems require the simultaneous use of mechanical and thermal actuation. DYNACORD, however, is a water-responsive SMP device triggered by body fluid [40,329]. This suture is composed of outer sheaths made of braided fibers made of ultra-high molecular weight polyethylene and polyester, and a unique core made of silicone and NaCl. When the suture is used during the surgery, it will be exposed to bodily fluids, which will dissolve the salt particles within the silicone core. As the salt elutes, the core becomes porous and hydrated with fluids. This hydration causes the core to swell radially, which in turn causes the braided outer sheath to contract axially due to Poisson's ratio of the material. For years, these devices were the only SMP medical products until Shape Memory Medical Inc. developed bioresorbable polyurethane foam for vascular applications, as explained in Section 2.1.1 [44,45]. This new platform includes peripheral embolization plugs, cerebral aneurysm coils, and investigational devices for aortic aneurysm sac management, all of which are in clinical trials (Table 3). These ongoing pivotal trials will shape the future of SMP-based therapeutics and shift the field toward clinical validation.

**Table 3:** Shape Memory Medical Inc. clinical trials on body-responsive SMP foams for vascular interventions, as of July 2025. Data collected from Clinicaltrials.gov.

Trial ID	Name	Device(s)	Medical Condition	Phase/Type	Status
NCT04044443	EMBO-PMS	IMPEDE®/IMPEDE-FX®	Peripheral vascular embolization	Post-market Registry	Recruiting
NCT04227054	AAA-SHAPE NZ	IMPEDE®/IMPEDE-FX RapidFill®	Abdominal aortic aneurysm	Safety Study	Active, not recruiting
NCT04751578	AAA-SHAPE_NLD	IMPEDE®/IMPEDE-FX RapidFill®	Abdominal aortic aneurysm	Feasibility Study	Unknown
NCT06029660	AAA-SHAPE	IMPEDE-FX RapidFill®	Endovascular aneurysm repair	Pivotal study	Recruiting
NCT03988062	APEX-FIH	TrelliX® Embolic Coil	Cerebral aneurysm	First-in-Human	Unknown
NCT06550986	FLAGSHIP_NZL	IMPEDE-FX RapidFill®	Aortic dissection	First-in-Human	Not yet recruiting
NCT06740721	FLAGSHIP_FRA	IMPEDE-FX RapidFill®	Aortic dissection	First-in-Human	Not yet recruiting

## 7 Concluding remarks and future perspectives

**Fig. 22:**
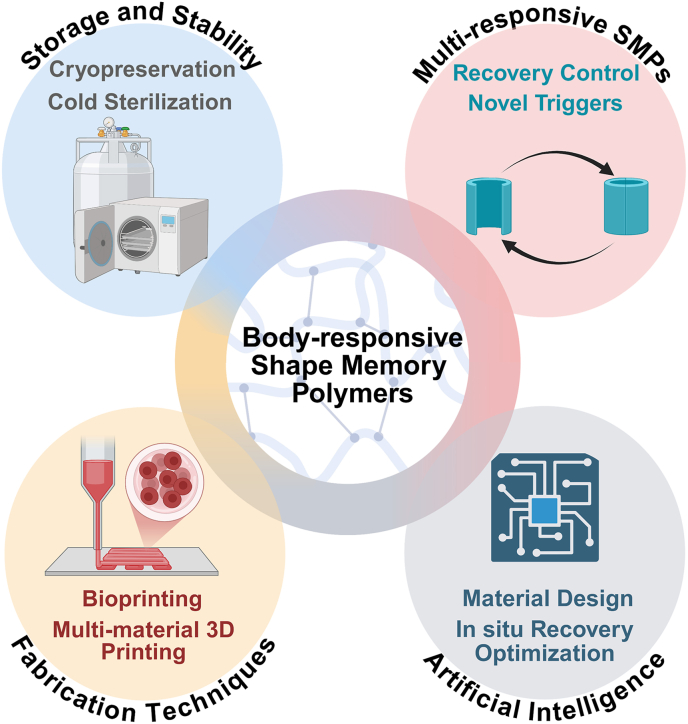
Future perspectives on potential advancements in the design and applications of body-responsive SMPs.

The emergence of body-responsive SMPs represents a significant advancement in biomedical engineering, offering dynamic and minimally invasive solutions for tissue repair and medical device development. Similar to how shape memory alloys have transformed surgical procedures and medical applications, the role of SMPs in tissue repair strategies and the development of novel treatments is revolutionary. Over the past few years, much research has been conducted to develop body-responsive SMPs for biomedical applications. However, this class of polymers is still in the early stages of research in the biomedical field, calling for continuous innovation in the years to come. Fig. 22 outlines key aspects of future perspectives for advancing body-responsive SMPs.

One significant challenge in utilizing body-responsive SMPs is the need to fine-tune their recovery behavior and mechanical properties within physiological environments. Under the body's aqueous conditions, molecular interactions within the SMP network may weaken, leading to a loss of structural integrity. This challenge could be addressed by combining different stimuli to design multi-responsive SMPs. Each stimulus can assist in a single aspect of the design, mimicking the complex native tissues. Beyond water- or temperature-responsiveness, we can integrate the emerging “cell-responsive” SMPs [[330], [331], [332]] into existing body-responsive mechanisms. This strategy helps to fully utilize the cells' biochemical cues, such as enzyme secretion or contraction forces, to induce targeted shape changes. Integrating all of these stimuli into one medical construct could spur research toward more in-situ tissue regeneration using body-responsive SMPs as we factor in cell response. Moreover, non-invasive external stimuli such as visible light [333,334] or high-intensity focused ultrasound [151,335] can be used for more precise control over the recovery of SMPs and their implantation in clinical settings. These strategies may pave the way toward the next generation of body-responsive SMPs with two distinct capabilities: 1) optimized recovery time, and 2) minimal induced damage to the surrounding tissues via recovery forces.

Artificial intelligence and machine learning have the potential to aid in the design of body-responsive or multi-responsive SMPs to control their shape-change behavior. This toolbox can equip researchers to predict in situ activation [336,337] and can inform the design of advanced SMPs [338,339] tailored for the target biomedical application. Moving toward clinical translation, multi-material printing could also be explored to introduce composite SMPs where each component contributes to a distinct response. This approach could lead to sophisticated structures capable of mimicking the complexity of native tissues using existing polymer libraries or employing new polymer designs. However, a critical limitation in current research is the lack of bioprintable SMPs, as they are mainly based on synthetic polymers. Synthetic polymers in this context fail to provide a cell-friendly environment. This challenge could be addressed by incorporating hydrophilic or natural polymers into the structure to enhance cellular interactions and biocompatibility. The design of novel hydrophilic polymers could further advance the field, bridging the gap between materials innovation and biological integration.

A major challenge hindering the clinical translation of body-responsive SMPs is their storage and preparation before clinical use. Considering the sensitivity of SMPs to heat and humidity, sterilization presents a significant hurdle, as the SMPs must be delivered through their programmed shape for seamless deployment. High-temperature sterilization techniques may compromise SMP's integrity. An alternative approach would be employing cold sterilization methods, such as gas sterilization using nitrogen dioxide. In cases where cell-laden SMPs are used for in situ tissue regeneration, cryopreservation is a practical strategy, as deep-freezing minimizes premature shape recovery. A critical concern with this strategy lies in the potential for the structural integrity of SMPs to deteriorate during deep-freezing, which warrants studies to ensure the long-term stability and functionality of the SMPs for biomedical applications.

Ultimately, the successful integration of body-responsive SMPs into real-world therapeutic applications hinges on material innovation, biocompatibility, behavioral characterization, and advanced fabrication. Continued interdisciplinary research can realize the full potential of body-responsive SMPs.

## Ethics approval and consent to participate

This document is not required for review manuscripts.

## Declaration of competing interest

The authors declare that they have no known competing financial interests or personal relationships that could have appeared to influence the work reported in this paper.
